# Using molecular simulation to understand the skin barrier

**DOI:** 10.1016/j.plipres.2022.101184

**Published:** 2022-08-19

**Authors:** Parashara Shamaprasad, Chloe O. Frame, Timothy C. Moore, Alexander Yang, Christopher R. Iacovella, Joke A. Bouwstra, Annette L. Bunge, Clare McCabe

**Affiliations:** a Department of Chemical and Biomolecular Engineering, Vanderbilt University, Nashville, TN 37235-1604, United States of America; b Multiscale Modeling and Simulation (MuMS) Center, Vanderbilt University, Nashville, TN 37235-1604, United States of America; c Division of BioTherapeutics, LACDR, Leiden University, 2333 CC Leiden, the Netherlands; d Department of Chemical and Biological Engineering, Colorado School of Mines, Golden, CO 80401, United States of America; e School of Engineering and Physical Science, Heriot-Watt University, Edinburgh, United Kingdom

**Keywords:** Stratum corneum lipids, Molecular dynamics simulation, Atomistic simulation, Coarse-grained models, Skin barrier function, Percutaneous permeability

## Abstract

Skin’s effectiveness as a barrier to permeation of water and other chemicals rests almost entirely in the outermost layer of the epidermis, the stratum corneum (SC), which consists of layers of corneocytes surrounded by highly organized lipid lamellae. As the only continuous path through the SC, transdermal permeation necessarily involves diffusion through these lipid layers. The role of the SC as a protective barrier is supported by its exceptional lipid composition consisting of ceramides (CERs), cholesterol (CHOL), and free fatty acids (FFAs) and the complete absence of phospholipids, which are present in most biological membranes. Molecular simulation, which provides molecular level detail of lipid configurations that can be connected with barrier function, has become a popular tool for studying SC lipid systems. We review this ever-increasing body of literature with the goals of (1) enabling the experimental skin community to understand, interpret and use the information generated from the simulations, (2) providing simulation experts with a solid background in the chemistry of SC lipids including the composition, structure and organization, and barrier function, and (3) presenting a state of the art picture of the field of SC lipid simulations, highlighting the difficulties and best practices for studying these systems, to encourage the generation of robust reproducible studies in the future. This review describes molecular simulation methodology and then critically examines results derived from simulations using atomistic and then coarse-grained models.

## 1. Introduction

Skin’s effetiveness as a barrier to permeation of water and other chemicals rests almost entirely in the outermost layer of the epidermis, the stratum corneum (SC), which consists of layers of terminally differentiated keratinocytes—corneocytes—surrounded by highly organized lipid lamellae that fill the extracellular space. As the only continuous path through the SC, transdermal permeation necessarily involves diffusion through these lipid layers whether transport through the corneocytes is or is not significant [1-4]. An important function of the SC is to act as a barrier to water loss from the body, which is supported by its exceptional lipid composition of primarily ceramides (CERs), cholesterol (CHOL), and free fatty acids (FFAs), and, unique from other biological membranes, the complete absence of phospho-lipids [5-7]. Unlike phospholipid bilayers, the water content of the SC lipid lamellae is low, only one or two molecules per lipid, and independent of whether the SC is or is not hydrated [8-11]. Hydration causes no measurable swelling of the lipid lamellae and changes phase behavior minimally if at all (see [10] and the references therein).

Lipids in normal, healthy SC are organized into two coexisting lamellar phases with repeat distances of approximately 6 nm (the short periodicity phase, SPP) and 13 nm (the long periodicity phase, LPP), with the LPP thought to be essential to skin barrier function [12-14]. The lateral packing of these phases is predominantly orthorhombic in human SC and hexagonal in pig SC [15-18], which are distinctive from the liquid crystalline packing in most other biological membranes. When properly equilibrated, CERs isolated from the SC of humans and pigs mixed with CHOL and FFAs in the appropriate amounts self-assemble into lamellar structures that exhibit the same phase behavior and organization as observed in intact SC [13,19,20]. Numerous investigations of extracted and reconstituted lipid mixtures have provided significant insights into the effects of lipid composition on lamellar and lateral organization and its relationship with barrier function.

More recently, the availability of synthetic CERs with known headgroups and chain lengths has allowed experimental inquiry into the role of individual lipid classes (CER, FFA and CHOL), and several CER subclasses, in the formation of molecular structures and a competent skin barrier. These studies have provided information on the lamellar phases, lateral organization, and, for some mixtures, barrier function (e.g., [21-25]), and also the locations of some lipid species within the SPP and LPP [8,26-29]. Despite a growing body of data and recent advances in experimental techniques, a clear understanding of the molecular-level structure of the SC lipid matrix and how it varies with changes in composition remains an elusive goal. Several models of molecular arrangements of SC lipid mixtures have been proposed (e.g., [8,13,27,30-33]), but the complications of performing and interpreting experimental results makes identifying the correct models difficult.

**Fig. 1.**
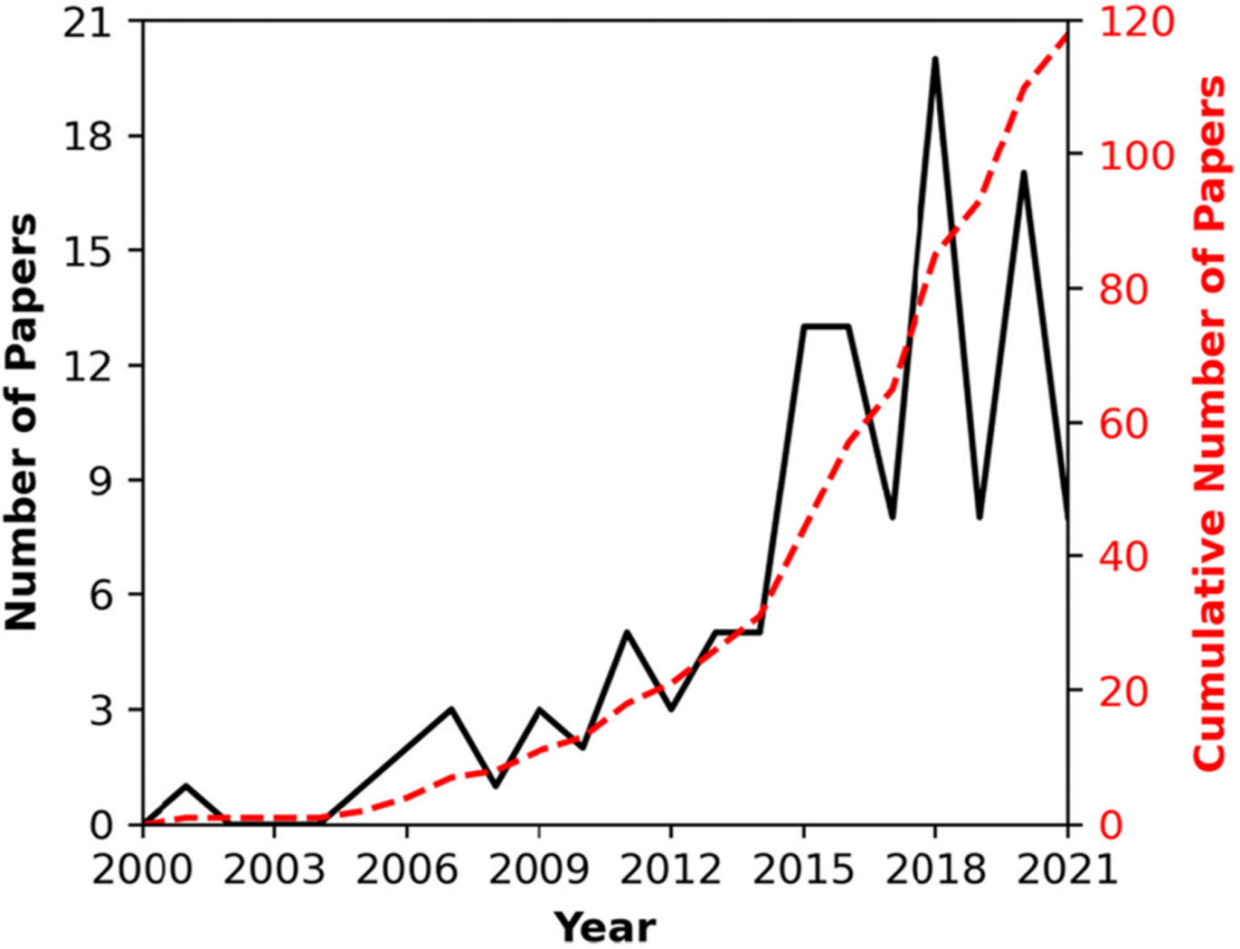
Number of original research papers published since 2000 that describe simulations of SC lipids in the context of skin (i.e., a simulation that includes CHOL may not be included).

Over the past twenty years, molecular simulation, which provides molecular level detail of lipid configurations that can be connected with barrier function, has become an increasingly popular tool for studying SC lipid systems. This trend is illustrated in Fig. 1, which shows the number of SC simulation papers published per year since the first publication in 2001. In this article, we review this ever-increasing body of molecular simulation studies of SC lipid systems. Our goals are to 1) summarize findings for researchers in this field, 2) provide a centralized summary of what simulations can and cannot do for those who do not specialize in molecular dynamics (MD) simulations, and 3) identify opportunities for future direction. As context for the molecular simulations, we begin with a brief summary of SC lipid composition, structure and organization. Molecular simulation methodology is then briefly described, with comments on specific aspects relevant to performing simulations of SC lipids, and the quantities that can (and cannot!) be obtained from simulations. An overview of the results presented to date from atomistic simulation studies are then discussed, with successes and problems highlighted. We then review coarse-grained models and simulations of those models, which are necessary to extend molecular simulations to the large systems and long-timescales (100 ns - μs) required to study SC lipid systems and their self-assembly. Finally, we summarize the current state of SC lipid simulations and look to the future.

## 2. Stratum corneum lipids

### 2.1. Composition, organization, and phase behavior

Barrier lipids other than CER, CHOL and FFA generally account for <5 wt% of the SC lipids, with cholesterol sulfate being the most abundant [34-37]. The relative amounts of CER, CHOL and FFA in human SC exhibit high inter-individual variability. A summary of data from many different groups shows CER content by weight is typically 45–65%, and mixed with 15–25% CHOL and 15% - 25% FFA [38]. These weight fractions, derived from thin layer chromatography (TLC), correspond to CER:CHOL:FFA molar ratios between 1:1:1 and 1:0.5:0.5 (based on average molecular weights of 387 for cholesterol, 368 for lignoceric acid, and 700 for CER). Recent lipid analyses performed using liquid chromatography–mass spectrometry (LC/MS, which identifies the thousands of different lipid species, allowing more precise accounting of mole fractions) show CER:FFA molar ratios (CHOL was not quantified) for native human skin ranging from <1 to >2 [39-41]. Differences in donors, body region sampled, and analytical methods all contribute to the observed variations. Evidently, therefore, formation of the SPP and LPP lamellar phases is relatively insensitive to deviations by these amounts from the equimolar composition of CER, CHOL and FFA used in many experiments. Consistent with this, studies with isolated CERs as well as synthetic CER mixtures show that the phase behavior remains essentially unchanged over a wide range of CER:CHOL:FFA molar ratios [13,20,42,43]. Insensitivity to CER:CHOL:FFA composition may be due in part to formation of a separate phase of crystalline CHOL when its solubility in the SPP and LPP lamellar phases is exceeded, which perhaps occurs at CHOL:CER molar ratios as low as 0.5 in an equimolar ratio of CER and FFA and varies with CER composition, the amount of FFA, and the presence or absence of CER EOS [22,44,45]. A phase separated CER-rich phase has also been observed in synthetic lipid mixtures with molar ratios of 1:0.5:0.5 [22]. CER:CHOL:FFA molar ratios that exceed the solubility of a lipid constituent are unlikely to exhibit phase separation on the timescales or size of a typical molecular simulation.

The FFA chains in healthy SC are generally long (ranging from 16 to 34 carbons, although predominantly 22, 24 and 26 carbons) and saturated [39,40,46]. Here, as elsewhere, the FFAs are notated as CX:Y, where X is the number of carbon atoms and Y is the number of unsaturated C─C bonds; a fully saturated FFA is designated as simply FFA CX.

**Fig. 2.**
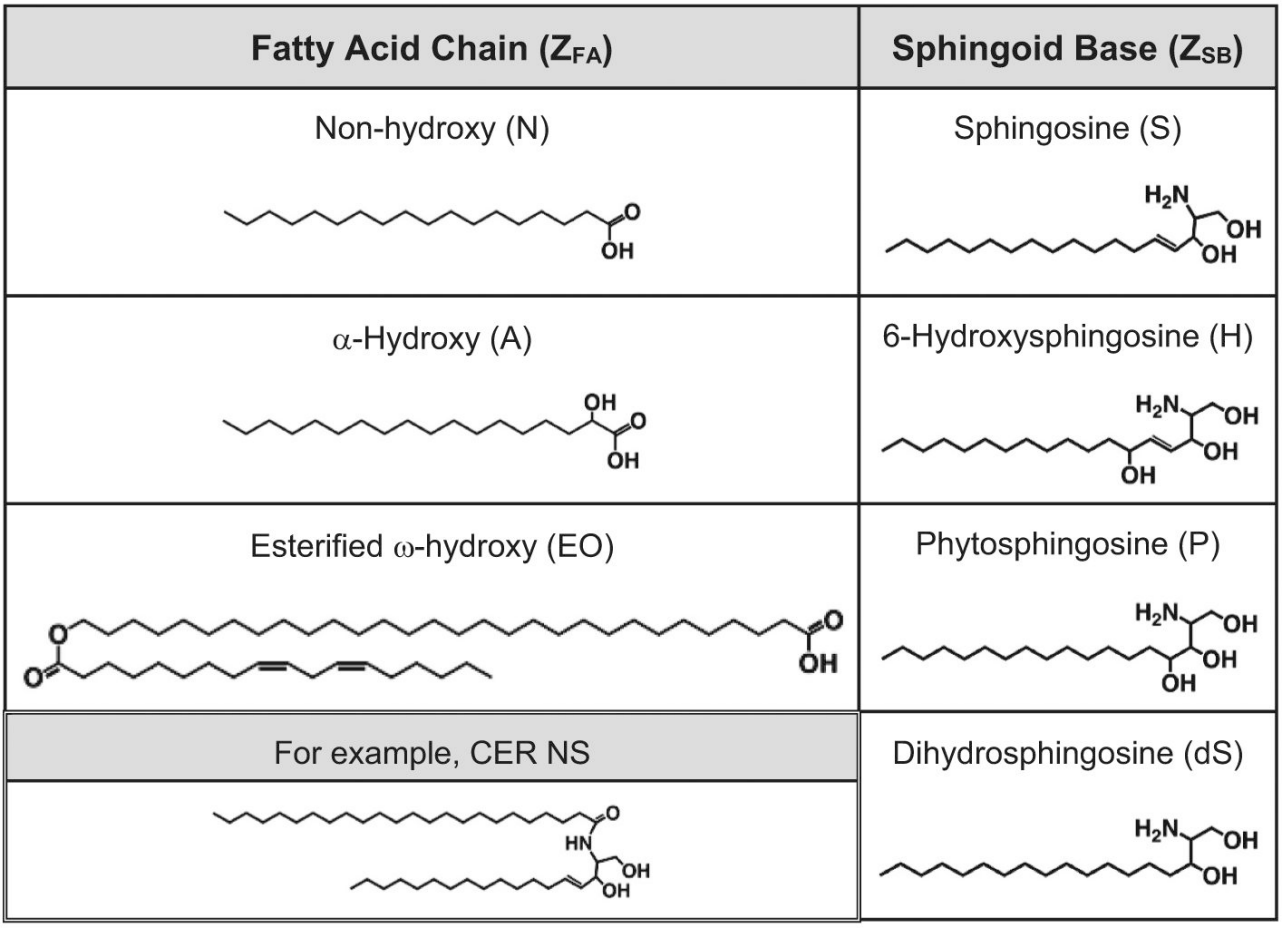
Structure and nomenclature for the 12 most prevalent CER subclasses found in human SC, designated as CER Z_FA_Z_SB_ where Z_FA_ and Z_SB_ represent the one or two letter abbreviations for the fatty acid and sphingoid base, respectively. The complete structure for CER NS is presented as an example.

CERs consist of a sphingoid base connected to a fatty acid by an amide bond. CER subclasses found in SC are commonly identified using the CER Z_FA_Z_SB_ notation [47], which designates by one or two letters the constituent fatty acid (Z_FA_) and sphingoid base (Z_SB_) as illustrated in Fig. 2 for the 12 subclasses that are most prevalent in human SC. When considering all CER subclasses, five fatty acids containing three different headgroups have been observed in human SC (see Fig. S1 in the Supplementary Information). The Z_FA_ designation for the non-hydroxy, alpha-hydroxy, and beta-hydroxy headgroups connected to a usually fully saturated straight hydrocarbon chain are N, A and B, respectively. The omega-hydroxy fatty acid (designated O) has a non-hydroxy head-group and a hydroxy group on the terminal carbon of the fatty acid chain (i.e., the φ position). The O fatty acid occurs in CERs on its own as well as ester linked in the φ position to linoleic acid, which is designated as EO [48,49]. In healthy SC, the fatty acid chains of the CERs are generally saturated and long, usually 16–32 carbon atoms [50] except for the O fatty acid chain, which is even longer (up to 38 carbon atoms); mainly 30–32 carbon atoms are observed for the O fatty acid and 24–28 carbon atoms for the others [51-53]. Five sphingoid bases have been observed in human SC (Z_SB_ designation listed in parentheses): sphingosine (S), phytosphingosine (P), 6-hydroxysphingosine (H), dihydrosphingosine (dS), and 4,14-sphingadiene (SD); Fig. S1. These exhibit slight variations in the aliphatic carbon chain length (16–20 carbon atoms), although C18 is most common [48-50]. In this paper, the number of carbons (X) in the fatty acid chain is specified as CX. Thus, CER NS C24 denotes a non-hydroxy fatty acid 24 carbons in length (i.e., lignoceric acid) linked to a sphingosine base (assumed to be 18 carbons in length unless designated otherwise). Tails of CERs with a C16 fatty acid linked to a C18 sphingosine base are approximately equal in length. In synthetic SC lipid mixtures, the O fatty acid chain in EOS is typically 30 carbons and usually specified.

In total 24 different subclasses of CERs have been identified in human SC (Table S5) [49]. These represent 21 of the 25 possible combinations of 5 fatty acids with 5 sphingoid bases, plus three other CERs (Fig. S1). One of these three, CER NT, consists of a new sphingoid base with four hydroxy groups (designated as T) seen thus far only in combination with the N fatty acid. The other two (identified as CER ENS and CER EAS) have a third tail produced by ester linking (E) a second fatty acid chain to the primary hydroxyl of the sphingosine in CER NS and CER AS [48,49,52,54]. Given the chemical heterogeneity of the CERs, including isomers, as well as the polydispersity in tail lengths and varying degrees of unsaturation in both the CERs and FFAs, more than a 1000 individual lipid components make up the human SC lipid matrix [51-53,55].

**Fig. 3.**
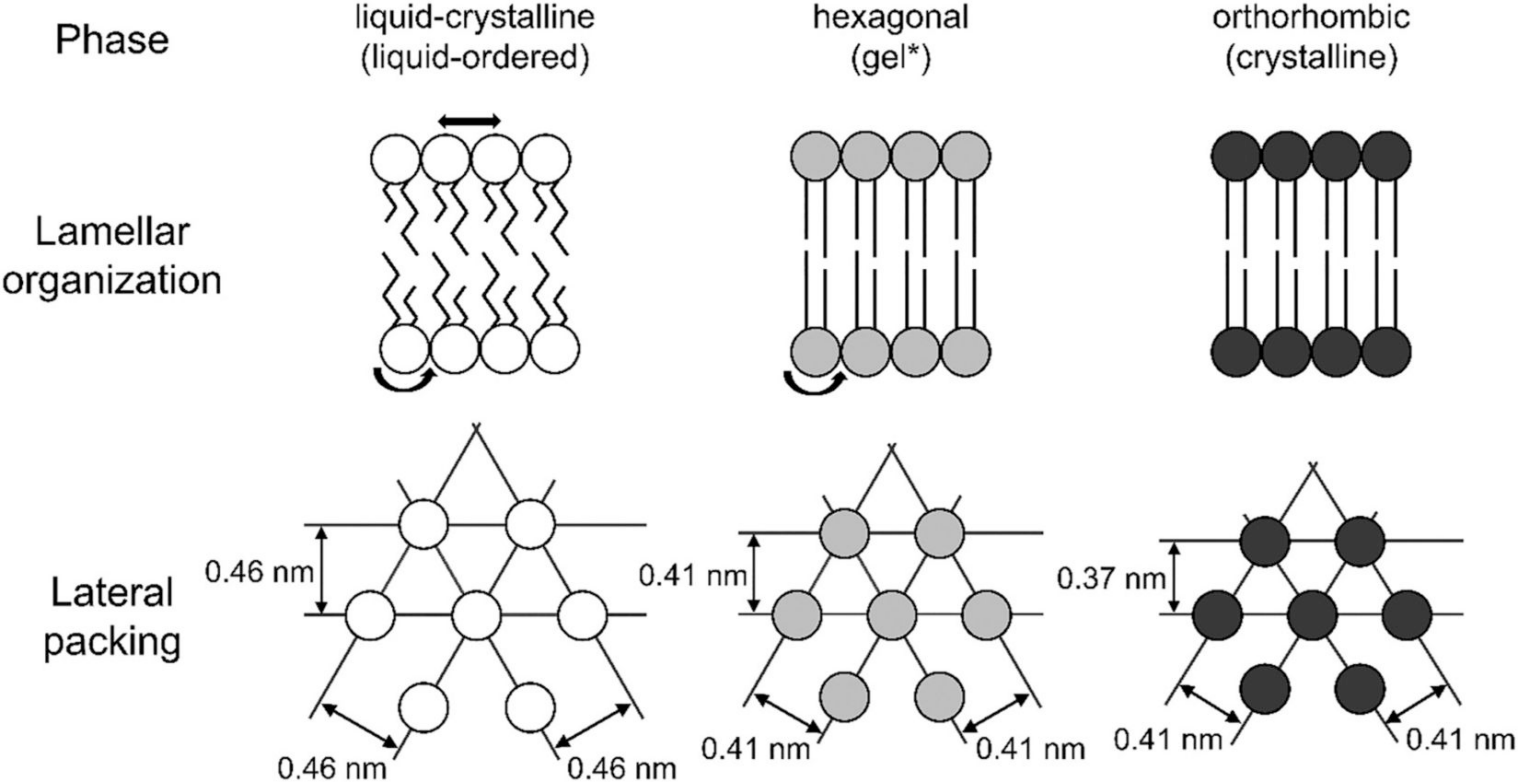
Schematic illustrating the organization and packing of the lamellar phases observed in the SC (redrawn from Pilgram et al. [67])). The phases are classified as orthorhombic or crystalline, hexagonal or gel, and liquid-crystalline or liquid-ordered. These terminologies are used interchangeably in the literature. Lipids in the liquid crystalline phase display lateral and rotational movements. In the hexagonal packing, hydrocarbon chains can rotate freely around their axes, whereas lipids in the orthorhombic packing are in solid state and packed more closely in one direction. *In this paper the word gel is also used in some contexts to describe a phase with limited mobility, which could be either orthorhombic or hexagonal.

SC lipid compositional profiles from individuals with skin disease, and also from 3D-cultured human skin models (sometimes called human skin equivalents), exhibit differences from healthy human SC. Recent studies show associations with altered expressions or activity of lipid biosynthesis enzymes [39,40,51,56-60]. Diseased or cultured SC lipids frequently exhibit reduced chain lengths in the FFAs and CERs, and increased fractions of monounsaturation in the FFA and CER [39,40,51,57,61]. Often these different lipid compositions lead to an impaired barrier function that is correlated with deviations in the lamellar and/or lateral organization of the lipids [24,25,56,62,63]. Compared with healthy human SC, reduced lamellar repeat distances, lower levels of the LPP, and a shift from a predominately dense orthorhombic lateral packing in healthy human SC to less densely packed hexagonal or liquid crystalline phases (Fig. 3) are common in diseased skin or human skin equivalents [18,56,62,64-66].

Initial investigations of SC lipid mixtures used CERs isolated from pig skin [19,20,68]. which were easily obtained in sufficient quantities. Pig skin was also widely used as a suitable surrogate for human skin in in-vitro permeation studies [69]. Experiments with equimolar CER: CHOL:FFA mixtures prepared with isolated porcine CER or isolated human CER exhibited a similar lipid organization [16,20]. Based on this and the available CER subclasses, subsequent studies using a fully synthetic lipid mixture, called the SC substitute (SCS) [70]. were based on CER and FFA compositions (Tables 1 and 2) observed in pig SC (with a substitution of CER NP C16 for CER AS C16, which was not available [71]) prepared in an equimolar ratio of CER, CHOL, and FFA. Porcine CER and the SCS based on synthetic porcine CERs differ from that in human SC (Table 1). In pig SC, the subclasses with the H sphingoid base (AH, NH and EOH) are missing, the most abundant CER is NS instead of the more hydrophilic NP (in human SC), EOS is the only member of the EO-subclass (EOP and EOdS are strongly reduced if present at all), and shorter CER chain lengths are more evident, in particular CER AS C16 [16,51,68].

Despite these differences, many studies of the SCS have demonstrated that it closely mimics phase behavior and lateral organization of human SC lipids [8,70,72,73]. A recent study examined a variation of the SCS in which the CERs are those that are most prevalent in human SC, but without any CERs from the 6-hydroxysphingosine (H) subclasses because synthetic versions of these are not available [25]. This ‘human’ SCS (H-SCS; see Table 1) showed almost no difference from the SCS. Both the SCS and H-SCS exhibit orthorhombic packing and the SPP and LPP lamellar phases, although the repeat distance of the LPP in the H-SCS was slightly increased due perhaps to the shorter CER chain lengths in the SCS [25]; similar results were observed in a slightly different human lipid model mixture although the LPP was less abundantly present (see Table 1 for compositions) [74]. A separate CHOL phase is observed in the pig and human variations of the SCS systems. Because the human SC model mixtures contain even larger amounts of the CER subclasses that hydrogen bond with themselves (see discussions in Sections 3.2.1 and 3.3.1), phase separation of CHOL or other components is likely to be more important in H-SCS systems.

The presence of the ultra-long EO-type (also called acyl) CERs (EOS, EOH, EOP and EOdS) are crucial for formation of the LPP, and the LPP is not observed when the EO subclass is absent [8,12,13,19,74]. Also, increasing the CER EOS levels causes an increase in the fraction of lipids forming the LPP with a corresponding decrease in the SPP [12,74,75]. Consistent with this, when the SCS is prepared without CER EOS, only the SPP forms [8], and when CER EOS is 40% of the total CER in the SCS, only the LPP forms [44]. It is possible, thereby, to separately study the SPP and LPP in systems with a similar organization and structure to the complete SCS by removing or adding more CER EOS [44]. although the amount of CER EOS required to form only the LPP can change with CER composition. Systems similar to the SCS but containing fewer CERs and/or FFAs can mimic behaviors of the SCS. For example, the SPP forms without the LPP in equimolar mixtures of CER NS, CHOL, and the 5-component FFA mixture (FFA5) of the SCS (Table 2) [27,33,76-79] or just FFA C24 [78,80], and the LPP forms without the SPP in these same systems when CER EOS is added in large enough amounts (e.g., CER EOS:CER NS is 30:70 or 40:60 mol%) [24,26,28,75,77,80].

In addition to the SCS and simplified SCS models, a rich body of experimental data exists for other mixtures containing only a few of the lipids in human SC; see the recent review [48]. Many of the simpler lipid models exhibit phases other than the SPP and LPP. For example, in addition to the LPP, equimolar mixtures of CER (40 mol% EOS mixed with either NS, NP, AS or AP), CHOL and FFA5 all include phase separated CHOL, as well as other phases in the NP and AP systems [24]. Likewise, equimolar mixtures of a single CER (NS, NdS, NP or NH) with CHOL and either FFA5 or FFA C24 plus 5 wt% cholesterol sulfate all exhibit phase separated CHOL; CERs NS and NdS also form a clearly defined SPP and one other phase for CER NdS, whereas CERs NP and NH exhibit other phases instead [81,82]. Some other examples of simple experimental systems include the equimolar mixture of CER NS C24 with CHOL and either FFA C16 or C24 [83], and 2:1 and 1:2 molar ratios of CER AP with either CERs NP or NS in a system with a 1:0.7:1 molar ratio of CER:CHOL:FFA C24 [84,85]. Important insights into the structure of the SC lipids have been derived from studying these simplified lipid systems. For example, using selectively deuterated lipids in the SCS or a simplified SCS, several recent investigations have located specific lipids or parts of lipids (e.g., the lipid head or tail) within the lamellar phases [8,26,27,29,33,28,86,106]. It appears from these studies that the CHOL headgroup tends to sit away from the unit cell boundary of the SPP and a significant fraction of the CERs are in an extended conformation [26,33,28,86,106].

### 2.2. Permeability in the stratum corneum and synthetic stratum corneum lipid membranes

Several experimental studies have investigated how the organization and structure of various lipid compositions affect barrier function by measuring permeation of model compounds (e.g., benzoic acid, derivatives of 4-aminobenzoic acid (PABA), hydrocortisone, theophyilline, indomethacin, urea, caffeine, diclofenac sodium) in diffusion cell experiments, transepidermal water loss (TEWL), or electrical impedance through membranes of SC lipids deposited onto porous polymer membranes. The results depend on the thickness of the lipid membrane (i.e., the lipid mass deposited per area on the substrate), which can be chosen to produce measurements that are comparable to those determined in diffusion cell experiments of excised skin samples [70].

Although the permeability measured through SC lipid membranes and excised skin can be similar, there are important differences in the chemical transport mechanisms of the two systems. If, as expected, the SC lipid membranes form lamellae that are oriented parallel to the support, then chemicals permeate perpendicular to the lamellae through a repeating series of lipid headgroups and lipophilic tails. In contrast, in excised skin chemical transport across the SC can include permeation through the corneocytes in addition to the lipid lamellae surrounding the corneocytes. If, as many have assumed, permeability through the corneocytes is small (i.e., nearly zero), then a chemical will move across the SC almost entirely through the lipid pathway, traveling parallel to the plane of the lipid lamellae. But if, as some others have speculated, corneocyte permeability is not almost zero [1,2,96,97]. then chemicals permeate perpendicular to the SC surface through the corneocytes in series with the lipid matrix surrounding the corneocytes (i.e., the transcellular pathway) in addition to the lipid pathway. If the corneocytes are highly permeable compared with the lipid lamellae, then transport across the SC might be estimated as exclusively perpendicular (transcellular) permeation through just the SC lipid lamellae because the corneocytes offer almost no resistance to penetration. It is only for this scenario of highly permeable corneocytes that the chemical transport mechanisms through the SC and SC lipid membranes would match.

Like the lipid matrix within the SC, the water content of the deposited SC lipid membranes is typically low—far too small to form coherent water layers between the lipid lamellae [8-10,98-100]. A typical sheet of isolated human SC contains approximately 15 corneocyte layers each separated from its neighbor by a lipid matrix layer of about 0.1 μm thickness. Thus, chemicals permeating through isolated SC traverse a total lipid matrix thickness of roughly 1.4 μm [1], which is about an order of magnitude thinner than the thickness of a SC lipid membrane with an equivalent permeability; for example, the thickness of the SCS lipid membrane had to be ~12 μm to match the permeability of ethyl-PABA through isolated SC [21]. A further consideration is that a macroscopic measurement like chemical permeability might be more sensitive to defects and non-uniformity in the deposition of multiple SC lipid layers than are the spectroscopic and biophysical methods used to characterize the structure and organization of SC lipid membranes.

For membranes prepared with different compositions of SC lipids, permeability increases have been associated with reduced lateral packing density (i.e., a lower proportion of lipids form an orthorhombic packing). This has been observed in equimolar mixtures of CER, CHOL and FFA with (1) shorter FFA chains [25,101], (2) monounsaturation of the FFA chains [102], (3) a larger distribution in the chain lengths of the CER [78,103] or the FFA [23], CERs with short acyl chains [63,78,81], and the presence of unnatural isomers of several CERs (NS, NdS, AS, AdS and AP) [79,104]. Systems with different composition but similar lateral packing density can exhibit similar barrier function, as observed, for example, in the comparison of equimolar mixtures of CER, CHOL, and FFA prepared with a combination of five CERs without CER EOS or with CER NS C24 alone [23]. However, this is not a general result, especially when comparing systems with different CER subclasses and headgroup architecture. For example, the increase in headgroup interactions that occurs when CER NS is replaced by CER AP in an LPP-only mixture (0.4:0.6:1:1 CER EOS: CER NS:CHOL:FFA) significantly reduced ethyl-PABA permeability even though the fraction of lipids in orthorhombic packing decreases [24]. In another example, equimolar systems of CER, CHOL and FFA in which CER EOS is 70% or more of a binary mixture with CER NS C24 exhibit increased levels of the orthorhombic phase but a reduced barrier function, apparently because these LPP-only systems also contain larger disordered lipid domains in the central layer in or close to the headgroup regions [75]. At lower more biologically relevant amounts of EO-type CERs, permeability is generally smaller in a system with an LPP compared to one with a similar composition but too little EO-type CERs to form an LPP [21,74,103].

In other variations of the SCS synthetic porcine CER mixture, benzoic acid permeation from water increased with the addition of CHOL sulfate, but was unaffected by elevated total CER or when oleate replaced lineolate on the CER EOS acyl chain [22]. When CHOL in the SCS was doubled, the amount of phase separated CHOL increased and benzoic acid permeability decreased [22]. However, reducing the amount of phase separated CHOL by decreasing its content incrementally from a mole ratio of 1 to 0.4 in an equimolar mixture of FFA5 (Table 2) and CERs isolated from human SC combined with 5 wt% cholesterol sulfate improved the barrier as determined by permeability of theophylline (MW = 180 and the logarithm of the octanol-water partition coefficient, logP, equal to approximately zero), whereas for indomethacin (MW = 358, logP ~4.3) permeability did not decrease with decreasing CHOL composition except at the 0.4 mol ratio, which was the largest CHOL concentration without phase separated CHOL [45]. The apparent contradiction of the two studies might reflect differences of the membrane systems in each study including the possibility that permeability reductions are only observed when the amount of separated CHOL is large. Other experimental differences might have affected the results. For example, theophylline and indomethacin were applied to the membranes in a 60:40 v:v propylene glycokwater solution, which might alter the lipid barrier. Other studies have showed 60% propylene glycol did not affect membranes prepared with a single CER (CER NS C16 or C24 in dissolution tests [78]. and CER NS C24, CER NP C24, or CER NdS C24 in permeability tests [81]) in an equimolar mixture of CHOL and FFA C24 or FFA5 with 5% cholesterol sulfate that formed only an SPP-like lamellar phase. But these observations on simple synthetic lipid compositions that formed only an SPP-like lamellar phase might not apply to membranes in the CHOL composition study, which contained isolated CERs (including many CER subclasses and a range of chain lengths) and co-existing LPP and SPP phases.

### 2.3. Molecular models of stratum corneum lipids

**Fig. 4.**
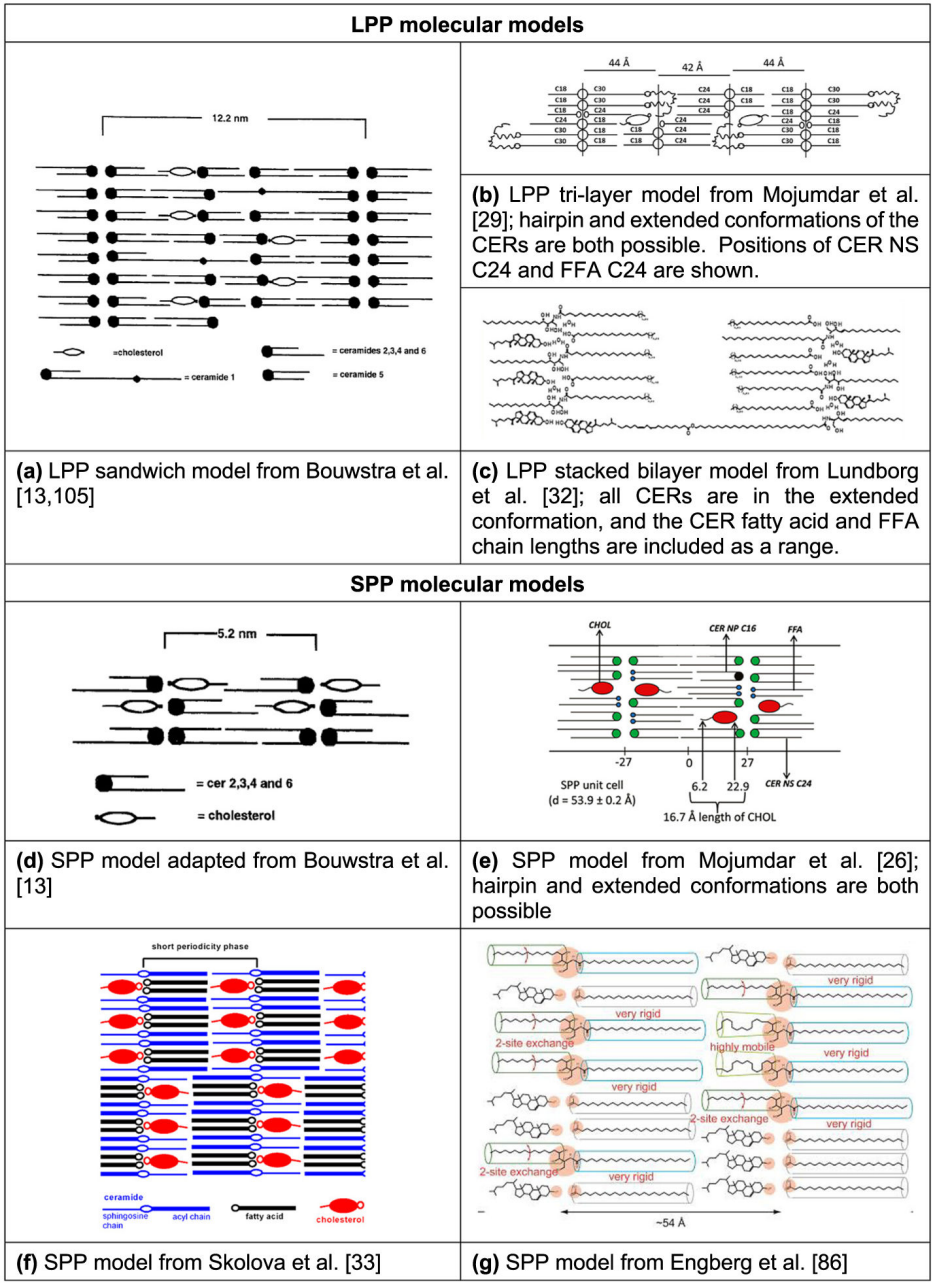
Proposed two-dimensional molecular model arrangements of the unit cell for the LPP (a-c) and SPP (d-g) in the SC lipid matrix. In (a) and (c) ceramides 1, 2, 3, 4 and 6 correspond to CERs EOS, NS, NP, AS, and AP, respectively. (All figures have been reprinted with permission from the appropriate journal publisher. (f) is reprinted with permission from “Different Phase Behavior and Packing of Ceramides with Long (C16) and Very Long (C24) Acyls in Model Membranes: Infrared Spectroscopy Using Deuterated Lipids” by Školová B, et al., 2014, *J Phys Chem B 118*, p. 10468. Copyright 2014 from American Chemical Society.)

Based upon the SC lipid composition and biophysical and nanoscale information of lipid organization, many different models of molecular organization have been proposed to describe the LPP and SPP of the SC lipid matrix, some of which are presented in Fig. 4. One of the first LPP models, the sandwich model (Fig. 4a), assumed a tri-layer arrangement, designed to match electron density profiles obtained by X-ray diffraction [13,105]. In this model the CERs are all in the hairpin configuration and a more fluid central layer, containing mainly CHOL and the unsaturated linoleic acid tail of the CER EOS, is sandwiched between more densely packed and less mobile layers containing long saturated hydrocarbon chains [13,105]. More recently, an improvement of the tri-layer model (Fig. 4b) was proposed based on a refined electron density model for the LPP and the locations of four lipids (linoleic acid tail of CER EOS, CHOL, acyl tail of CER NS C24 and FFA C24) within the LPP-only version of the SCS [27,29,72]. In this model the central layer contains the lineolate tail of the CER EOS as well as the acyl chain of CER NS and FFA, but no CHOL, which is only present in the outer layers. CER EOS links the central layer to the outer layer, where its ester bond is located in the outer layer at a short distance from the central layer boundary at the same position as the CHOL headgroup. In addition, observations from FTIR and neutron diffraction show that the CER NS C24 in the central layer is primarily in the extended conformation [28,106]. In contrast with the symmetry of these models, authors of the stacked bilayer model of the LPP (Fig. 4c) proposed an asymmetric lipid arrangement, in which the fatty acid chains of the fully extended CERs are associated with the FFAs and the sphingoid base chain with most of the CHOL [32,107]. In this arrangement, chosen because the electron microscopy patterns generated from pre-assembled simulation models can match those observed experimentally, the fully extended CER EOS crosses the entire bilayer. However, neutron and X-ray diffraction studies show no evidence for the proposed asymmetry of this model.

An early model of the SPP (Fig. 4d) adopted a symmetric bilayer organization of two opposite interdigitating CERs or an interdigitating CHOL and CER that matched the experimentally observed distance between two regions of high electron density [13]. Since then, locations of CHOL, FFA and CER NS C24 within the SPP of the SPP-only SCS have been derived in a series of neutron diffraction studies combined with selective deuterium substitution of these lipids [8,26]. Consistent with these observations in the SCS containing five CER subclasses, Mojumdar et al. [26] (Fig. 4e) proposed an SPP model in which CER NS is arranged symmetrically within the unit cell, the CHOL headgroup position is slightly inward from the unit cell boundary with its tail located about 0.6 nm from the unit cell center, and the longer chains of the FFA and CERs straddle the center of the unit cell. Whether CER NS is in a linear or hairpin conformation could not be concluded from these data. Based on observations from Fourier transform infrared spectroscopy (FTIR) of CER NS C24 mixed with CHOL and either FFA C24 or the FFA5 mixture (Table 2), Skolova et al. [33] assumed in their SPP model (Fig. 4f), as hypothesized by Iwai et al. [31] and also by the LPP stacked bilayer model [31,32], an asymmetric arrangement in which the CERs are fully extended and their acyl chains associate with only the FFAs and the sphingoid chains with only CHOL. Skolova et al. hypothesized further that these asymmetric layers must form regular alternating domains so that the average neutron scattering distance profiles could be consistent with the symmetry observed in experiments [33]. A variation of this model from Engberg et al. (Fig. 4g) [86], based on FTIR and NMR observations of the CER NS C24, CHOL and FFA C24 mixture, allows a portion of the sphingosine chains to be dynamic. To avoid packing defects from mismatches in the lengths of the mobile and rigid sphingosine chains, they hypothesize the formation of separate clusters of mobile and rigid chains.

Conclusive experimental validation of any of these, or other, models is extremely difficult, especially since the various techniques used in the many studies provide different views of a complex system. For example, FTIR measurements can be used to identify lateral packing, conformational ordering, mixing behavior, and the presence of hydrogen bonding, while X-ray diffraction provides information on the lamellar phases as well as lateral packing. However, with both methods no direct information can be obtained on the location of a specific lipid type within the SPP or LPP. This can be obtained by neutron diffraction, but with these techniques “mean arrangements” will be obtained. In addition, differences in lipid composition (single versus a mixture of CER subclasses for example) might affect the results. Also, ensuring equilibrated systems is a complication of all experiments with lipid mixtures in solid phases that must be considered. Furthermore, the choice of the spraying method, spraying surface, distance between the nozzle and spraying surface, the gas flow rate, and the temperature of annealing are crucial for the LPP formation and suboptimal choices for these variables (e.g., causing droplet creation) may result in less efficient development of the LPP and the formation of additional phases [108,109]. For example, suboptimal conditions in these parameters may be why systems with similar lipid compositions form an LPP with no SPP in one study [80] but no LPP at all in another [88]. Discerning molecular organizations and configurations in these mixed lipid systems is thus challenging, requiring careful techniques and creative methodologies that are used in combination. Computational testing of hypothesized molecular arrangements through molecular simulations, especially when combined with related experimental work, offers an opportunity to confirm and further explore lipid organization, pushing forward our understanding of structure, phase behavior and barrier function of SC lipids.

### 2.4. Lamellar structures of simulated stratum corneum lipids

**Fig. 5.**
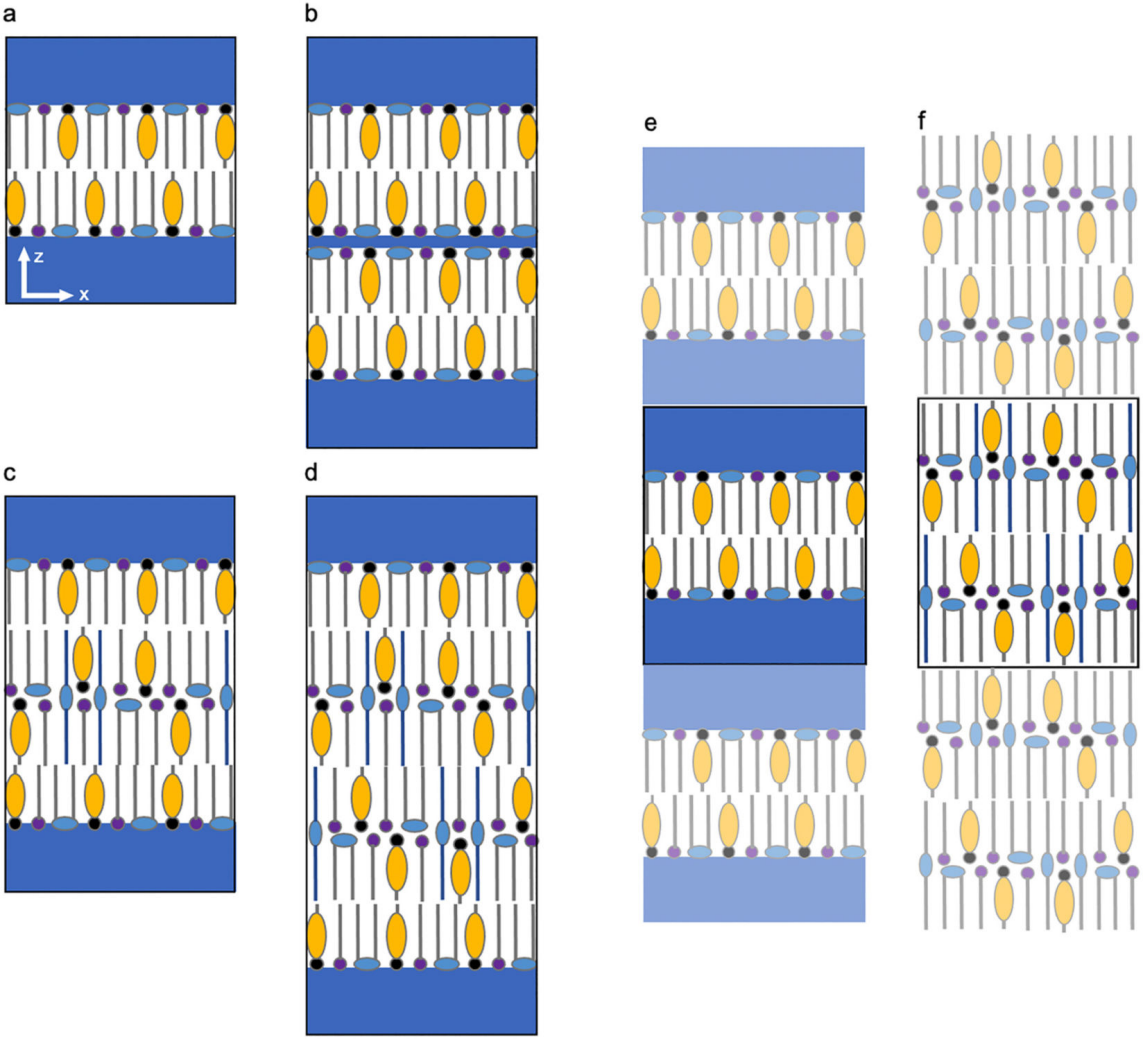
Schematic illustrations of (a) a hydrated bilayer (HBL); (b) a two hydrated bilayer (4-leaflet) stack (2-HBL), which includes water slabs in contact with headgroups of the two outer leaflets and intermembrane water between the bilayers; (c) a 4-leaflet hydrated multilayer stack (4-HML), which includes a water slab on the headgroups of the outer leaflets but no intermembrane water between the bilayers; (d) a 6-leaflet hydrated multilayer stack (6-HML); (e) a simulation box containing one hydrated bilayer (HBL) with periodic images above and below, and (f) a simulation box containing a 4-leaflet dehydrated multilayer stack (4-DML) with periodic images above and below. In (e) and (f) the simulation box is highlighted and the periodic images are faded.

Simulation results depend on the pre-assembled or self-assembled lamellar structure of lipids in the simulation box. In this review we use a standardized nomenclature based on the leaflet, defined as the plane of molecules that forms one half of a bilayer, which span the simulation box in the *x* and *y* directions. Thus, a bilayer (Fig 5a) has two leaflets, a double *stacked* bilayer has four leaflets (Figs. 5b and c), and so on. The headgroups of *hydrated* bilayers (Fig. 5a) are in contact with a water slab typically containing 5 to 40 water molecules per lipid. *Hydrated bilayer stacks* also include intermembrane water between the bilayers (Fig. 5b); the amount of intermembrane water could be the same or less than in the water slab on the headgroups of the outer leaflets. *Hydrated multilayers* include a water slab on the two outer leaflets but no intermembrane water; for example, Figs. 5c and d show four-leaflet and six-leaflet stacks with water only on the headgroups of the outer leaflets. A *dehydrated multilayer stack* contains no water (Fig. 5f); these can be constructed with either the lipid tails (as in Fig. 5f) or the headgroups pointing out of a two-leaflet (bilayer) stack.

In molecular simulation, periodic conditions are usually applied to the boundaries of the simulation box to approximate a system that is infinitely repeating in either two (*xy*) or three (*xyz*) Cartesian coordinates such that an object passing through one side of the simulation box appears on the opposite side of the box with the same velocity (as illustrated in Figs. 5e and f). In the case of a hydrated lipid bilayer, periodic boundary conditions means that the simulation box represents an infinite stack of infinitely wide repeating bilayers separated by a slab of water (Fig. 5e). The hydrophilic interface between the lipid bilayers and water forces the CERs into a hairpin conformation, where both tails point in the same direction into the bilayer, except when the number of water molecules per lipid is small (probably not >2–3). An extended or splayed CER conformation, where the tails of a CER molecule point in opposite directions, requires a stack of two or more leaflets with no water between the headgroups (i.e., Figs. 5c, d and f) or only a small amount of water between headgroups (i.e., Fig. 5b or Fig. 5e with a very thin water slab above and below each bilayer). As examples of this latter situation, approximately 35% of the CERs were in the extended conformation in the interior bilayers of both a four- and a six-leaflet stack containing CER NS C24:CHOL:FFA C24 at a 1:0.5:1 molar ratio that self-assembled (using a coarse-grained model described in Section 4.2.2) with <3 intermembrane water molecules per lipid [110].

## 3. Atomistic molecular simulation of stratum corneum lipids

The main goal of molecular simulation is to examine how molecular-level interactions give rise to the properties of physical systems. This is achieved by generating a set—or ensemble—of molecular configurations for a given system and calculating properties from these configurations. These properties can then be connected to macroscopic observations through statistical mechanics. The ensemble can be constructed such that it samples from a given thermodynamic ensemble. For example, since molecular simulations of SC lipid systems aim to compare properties with experimental systems at laboratory conditions, simulations are generally designed to sample from the isothermal–isobaric ensemble (commonly referred to as the NPT ensemble for the constant number of molecules (N), pressure (P) and temperature (T)).

Two general types of molecular simulation methods are used to generate molecular configurations. Monte Carlo (MC) methods generate configurations by stochastic displacements of atoms, which are either accepted or rejected based on criteria designed to ensure that the desired ensemble is sampled. As configurations are generated stochastically, MC simulations have no connection to time, and hence dynamic properties such as diffusion cannot be calculated. In contrast, molecular dynamics (MD) methods employ classical mechanics to numerically calculate the trajectory of the systems through time based on the forces between the atoms. As MD naturally contains an associated timescale, dynamic properties can be calculated. In the past, MC had been infeasible for dense systems such as lipid bilayers because no MC moves would be accepted due to overlapping atoms. However, methods such as configw [111] and hybrid MC [112]. as well as more complex MC moves have made the simulation of lipid bilayer systems possible using MC methods [113]. The main barriers for adoption of these new MC methods is the difficulty in implementing these algorithms and the lack of available open-source and easy-to-use software for such simulations. Therefore, MD remains the predominant method for studying SC lipid systems and lipid membranes more generally.

In a MD simulation, the system is initialized with a given molecular configuration, and the forces on the atoms are calculated based on the interactions between the atoms; these interactions are encoded in a “force field.” Based on these forces, the positions of the atoms are updated by numerically integrating the equations of motion. Timesteps are generally on the order of 1–2 fs and are limited by the fastest motions in the system (e.g., bond vibrations involving a hydrogen atom). The first step of the simulation is often referred to as “equilibration,” in which the goal is to allow the system to relax (and, if appropriate, move away) from its initial molecular configuration and reach a steady state where the system is no longer changing. Steady state is often determined by examining thermodynamic quantities, such as potential energy as a function of time, and/or by examining structural measurements, such as in-plane density or lipid-lipid correlation numbers. Although this stage is referred to as “equilibration,” achieving a steady state only indicates that a local energy minima has been reached and does not necessarily imply thermodynamic equilibrium has been achieved; the steady state achieved may depend on the initial configuration and exact procedures/parameters used during this equilibration stage [114-116]. After equilibration, the “production” portion of the simulation is performed, generally for 1–100 × 10^6^ timesteps (one to a few hundred ns of simulation time), and the configurations and thermodynamic properties are saved at specified time intervals. These configurations are referred to as the simulation trajectory. The simulation trajectory is then usually post-processed to calculate the properties of interest.

### 3.1. Force fields for simulating stratum corneum lipids

Typically, the force field consists of bonded interactions (between atoms that are covalently bonded) and non-bonded interactions (between all atoms). The bonded components include the bond stretching, angle bending, and torsional forces. Bonded forces are often described by simple harmonic spring equations, which mimic the atomic vibrations of covalently bonded atoms. The non-bonded components include long-range electrostatic forces and short-range van der Waals forces. Electrostatic forces are usually calculated using Coulomb’s law and the magnitude of these forces dictated by atomic partial charges. Van der Waals forces are typically represented using a variation of the Lennard-Jones equation in which the sigma and epsilon values (representing respectively the distance at which the particle-particle potential energy is zero and the depth of the potential energy well) are empirically fitted to match ab initio (quantum mechanical) calculations or experimental data.

Of the available “generic” force fields, by which we mean open source force fields applicable to a wide range of chemistries, the CHARMM [117] and GROMOS [118] force fields are perhaps the most commonly used in the study of lipid systems. The CHARMM force field is fully atomistic, meaning that each atom is explicitly represented in the simulation. Whereas, the GROMOS force field uses a “united atom” model, in which CH_n_ groups are treated as a single interaction site. While each force field has its own strengths and weaknesses, the majority of the atomistic SC lipid simulations to date have been performed with versions of either the CHARMM or the GROMOS force field, often with small tweaks to the parameters to better match ab initio calculations or experimental data for the specific lipid molecules of interest. As such, when modifications are made to a force field, the full set of parameters used in the publication needs to be reported in order for others to be able to reproduce the results. The GAFF (atomistic) and OPLS-UA (united atom) force fields have also been used in simulations of CER systems. Bonded and van der Waals parameters for the GROMOS, GAFF, and OPLS-UA force fields are based on atomic hybridization states and, unlike the CHARMM force field, are not molecule-specific. Generally, the only modifications (if any) made for new molecules using the GROMOS, GAFF, or OPLS-UA force fields are recalculating partial charges because bonded interactions are generally already established. For the CHARMM force field, where parameters are often specific to individual molecules, additional bonded parameters are often also calculated for new molecules.

Table 3 lists the various atomistic and united atom force fields that have been used to simulate SC lipids and their lamellae. The earliest simulations of CERs were by Pandit and Scott who used a modified version of the GROMOS96 43A1 (a specific version of GROMOS) force field in which new partial charges for the atoms in CER NS C16 were derived from ab initio calculations [119]. This modified force field is referred to as GROMOS-Scott. However, the GROMOS force field was originally optimized for short alkanes and known to produce inaccurate densities and heats of vaporization when applied to long lipid chains [120,121]. Berger et al. [121] added corrections to the van der Waals parameters of the GROMOS force field to account for long aliphatic lipid tails, creating the GROMOS-Berger force field. Notman et al. [122] subsequently applied the GROMOS-Berger force field to study a CER NS bilayer system by using bonded parameters from a previously validated dipalmitoylphosphatidylcholine (DPPC) model [123], and using partial charges from a previously validated serine side chain model [124] for the hydroxyl and amide functional groups. Although the actual parameters are not reported in the publication [122], the procedure for determining the parameters is well documented. The force field used in Notman et al. [122], identified here as GROMOS-Notman, has served as a starting point for numerous other studies. However, because Notman et al. [122] did not report their parameters, the exact implementation of the force field in studies that utilized GROMOS-Notman may have been different. Das et al. in 2013 determined new partial charges in order to apply the GROMOS-Notman force field to CERs NP and EOS [125]. These new charges, which were reported with their publication, are designated as the GROMOS-Das force field. New CER AP parameters for the GROMOS-Notman force field were used in simulations described by Schmitt et al. [85] but the actual parameters, the necessary details of how the parameters were obtained, or their validation, were not reported, making the results unreproducible. More recently, Badhe et al. used the GROMOS-Notman force field to simulate bilayers of CERs NP, NdS, NH, AH, AP, AS and AdS all with C24 fatty acid chains [126]. Unfortunately, once again, the actual parameters, as well as details of the charge assignment, parameter optimization, and validation were not provided.

Anishkin et al. were the first to use the CHARMM force field to simulate CERs in their study of pores in CER NS C16 and POPC (1-palmitoyl-2-oleoyl-sn-glycero-3-phosphocholine) bilayers in 2006 [127]. Although the force field parameters are reported in their publication, details of the force field derivation were not provided [127]. Imai et al. used the CHARMM27 (where 27 designates the CHARMM version) force field to simulate CERs in their study of pure CER NS bilayers in 2010 [128]. However, CHARMM27 does not contain the parameters necessary to describe CERs (specifically the bonded parameters involving the amide bond as well as partial charges for atoms in the headgroup). These parameters can be taken from parameters used to describe peptides, but this requires choices to be made that are not described; therefore, once again, the work cannot be reproduced because the specific model parameters used were not reported. Engelbrecht et al. used the CHARMM27 force field to model a bilayer of CER AP and a novel branched CER EOS molecule mixed with FFA C22 and CHOL [129]. In this work the authors reported that the CER headgroup parameters were taken from existing parameters for sphingomyelin and serine molecules and were thus not optimized to study CERs. However, the actual parameters used were again not provided [129].

Guo et al. [130] were the first to optimize the CHARMM force field for CERs by parameterizing bonded parameters and charges for CER NS and CER NP headgroups to be used in conjunction with the CHARMM36 force field (identified below as CHARMM36-Guo) [117]. In addition, Guo et al. compared results from the CHARMM36-Guo force field to those from the GROMOS-Notman force field for pure CER NS bilayers, finding that the former can better reproduce experimental calorimetric data. In separate work by Venable et al. [131], new parameters for CERs and sphingomyelin were added to the CHARMM36 force field. Specifically, bonded parameters for CER NS and CER AP were taken from the CHARMM36 force field, with the exception of dihedrals involving the amide group, which were fit to match ab initio calculations of fragments of the CER NS and CER AP headgroups. Partial charges for sphingomyelin were calculated from ab initio calculations of a sphingomyelin headgroup; however, the derivation of the partial charges for CERs was not explained. Wang and Klauda were the first to use these new parameters to simulate CER NS and CER AP systems [131,132]. The CER NS bonded parameters and partial charges used by Wang and Klauda (henceforth referred to as CHARMM36-Wang) are different from the CHARMM36-Guo parameters due to small differences in the ab initio calculation methods used to obtain the parameters. However, it is difficult to determine whether differences in the structural parameters between these force fields (listed in Tables 4-6) are indeed due to differing force field parameters or just differing equilibration methods. The CHARMM36-Wang parameters for CER NS and CER AP are available online on the CHARMM-GUI website (http://www.charmm-gui.org) [133]. In more recent work, Wang and Klauda developed CER EOS force field parameters to simulate pure [134] and mixed CER EOS systems [135]. Although the method for parameterizing the ester linkage is not provided, the parameters are reported on the CHARMM-GUI website. Finally, Lundborg et al. reoptimized headgroup atom types, partial charges and bonded parameters for CER NP based on the CHARMM36-Wang force field to reproduce ab initio calculations [32]. These new parameters (designated CHARMM36-Lundborg) were able to reproduce the experimentally observed V-shaped crystalline structure of CER NP, which was not possible using the CHARMM36-Wang parameters [32,136]. Lundborg et al. also simulated systems containing CER EOS, details of the force field parameter derivation for CER EOS were not provided. The CHARMM36-Lundborg parameters are reported by Lundborg et al. [32].

We now consider the properties of SC lipid systems that have been calculated from atomistic simulations using these force fields in the literature. Many properties such as repeat distances, area per lipid, neutron scattering and electron density profiles, carbon-deuterium order parameter, lateral compressibility, thermotropic phase behavior, and permeability can be compared directly with experimentally measured values (with some caveats as discussed below). In addition, we highlight properties that may be difficult or impossible to obtain from experiments, such as tilt angle, detailed hydrogen bonding information (direct quantification of the number of hydrogen, atoms involved and lifetimes), and exact molecular positions and arrangements.

### 3.2. Calculations of general structural and mechanical properties

Because molecular simulation yields molecular-level resolution, calculating structural properties from a simulation trajectory is straightforward. Several general structural properties are ubiquitous in the SC simulation literature, and the lipid membrane simulation literature in general. These include the area per lipid (APL), area per lipid tail (APT), density profiles, bilayer thickness, tail tilt angle, tail interdigitation, and several lipid tail order parameters.

#### 3.2.1. Area per lipid

The APL is a measure of the in-plane density of the lipids, and hence may be used as a metric to determine a phase transition, where the dense, well-ordered state of the membrane will have a smaller APL than the fluid phase. APL is generally taken to be the cross-sectional area of the simulation box divided by the number of lipids in each leaflet. Because CERs have two tails and FFAs only one, pure CER bilayers display a larger APL than mixed CER-FFA or pure FFA bilayers. Thus, conclusions about the “free area” available in a bilayer may be clouded by compositional variations when comparing the APLs of multicomponent bilayers. For this reason, an area per tail (APT), defined as the average area occupied by each tail in the plane normal to the long axis of the lipid tails [139], has been reported, e.g. [115], which is calculated by multiplying the APL by cosθ, where θ is the tilt angle, and then dividing by the average number of tails per lipid [139]. Because cosθ≤1, APT has a smaller value than simply normalizing APL by the average number of lipid tails. However, in SC lipid systems, which generally exhibit small tilt angles with minimal compositional dependence [115], the cosθ adjustment is often insignificant. APT values have been reported without the cosθ term, e.g. [138,140], and thus authors should specify how APT values are calculated.

Because CHOL has a larger cross-sectional area per tail than either CERs or FFAs, APT is only suitable for evaluating lipid packing in SC lipid mixtures without CHOL. To address this issue, Shamaprasad et al. [110] proposed a new metric, the normalized lipid area (NLA), in which the APL is divided by the *effective* number of hydrocarbon tails per lipid, where FFAs have one, CERs have two, and CHOL has 1.9 (estimated from the ratio of the experimental cross sectional areas for CHOL and hydrocarbon chain; 38 Å^2^ and 20 Å^2^ [141]). Although NLA could, like the calculation of APT, be multiplied by cosθ, here we choose to define it within the plane normal to the membrane interface (i.e., without cosθ) because cosθ is likely to be close to one. Also, because tilt angle is not always reported, this definition of NLA allows for a consistent comparison among all the studies reported herein.

In principle, Voronoi tessellation analyses [142,144] can be used to calculate directly the in-plane area occupied by individual lipid components in a mixture of lipid components, allowing comparisons of packing between systems with different lipid compositions. However, such measurements will not be accurate if they do not account for the two tails in CERs and the shape/size of CHOL, as discussed above in the context of APL and APT. For example, Podewitz et al. [145,146] report APL values for the individual CER NS C24, CHOL and FFA C24 components in their mixtures, calculated using the Voronoi method. In their analyses, each lipid molecule was represented as a single bead chosen from the respective coarse-grained model, and thus, the reported values underestimate the APL for CER and CHOL and overestimate the APL of FFA: similar APL values are found for each of the three lipids molecules. Del Regno and Notman [147] also reported the APL of individual components in mixtures of CER NS C24, CHOL and FFA C24 calculated with a Voronoi analysis that used a representative atom in the head-group to locate each lipid; their results (provided in the supplementary information of their paper) are similar to those from Podewitz et al. [145,146]. In a variation of Del Regno and Notman’s Voronoi analysis, Wang and Klauda [135,143,148] assigned a representative atom to each lipid tail. As a result, they calculated APL values for CERs EOS, NS, NP and AP in mixtures with CHOL and FFA that are consistently about twice the values calculated for both CHOL and for FFA.

Table 4 summarizes APL values reported in published simulation studies for bilayers of pure CER NS C24. Variability is large, ranging from 37.7 to 46.0 Å^2^. For comparison Langmuir monolayers of pure CER NS C24 at an air-water interface were reported to have an APL of ~42 Å^2^ [149,150]. However, experimental monolayers may behave differently than gel-phase multilayer systems. (Note that here and elsewhere in this review, the word gel is sometimes used to describe a phase with limited mobility without distinguishing whether it is orthorhombic or hexagonal; see Fig. 3). On average, CHARMM-based force fields produce a higher APL (42.0 Å^2^) compared with GROMOS-based force fields (40.7 Å^2^). Within CHARMM-based force fields, the CHARMM36-Guo force field yields lower APL values compared with the CHARMM36-Wang force field [130,134]. Discrepancies in the values from calculations using the same force field are likely caused by varying equilibration processes and run times (if an equilibrated state has not been achieved, then the results of a simulation may not be independent of the simulation time). For example, some authors use an annealing process to accelerate the equilibration process [115], while others attempt to equilibrate the gel-phase bilayer at constant temperature in the system using longer (>500 ns) simulation times [134]. The challenges of equilibrating gel-phase SC bilayers are discussed below (see Section 3.4).

The APL from simulations of pure CER NS C16 bilayers (Table 5) are found to be similar to those for CER NS C24; this is most clearly evident in studies that examined both C16 and C24 (e.g., Moore [115,151] and Wang [134,143] in Tables 4 and 5). Paloncyova et al. also considered pure bilayers of CER NS with acyl tail lengths shorter than C16 and found that CERs with very short tails (<C6) exhibit smaller APLs compared to those with longer tails up to C12; CERs with acyl tail lengths of C12 and longer had roughly the same APL [152]. Thus, APL is a good measure of headgroup packing, and is independent of the tail length for CERs with the most commonly observed acyl tail lengths (i.e., C16-C26). For comparison, Langmuir monolayers of pure CER NS C16 were reported to have APLs of ~39–10 Å^2^ [149,150].

Limited studies have compared the APL of pure bilayers composed of different CERs. In simulations using the CHARMM36-Guo force field, Guo et al. observed that the APL of CER NP C16 (42.1 Å^2^) which is essentially the same as that for CER NS C16 (42.4 Å^2^) [130]. which indicates that CER NP, despite having an extra hydroxyl, packs similar to CER NS. This was attributed to a stronger hydrogen bond network, which increases the cohesiveness of the CER NP headgroups. Increased hydrogen bonding was also observed in FTIR experiments of pure CER NP compared to CER NS [153]. In contrast, Wang and Klauda observed in simulations of CER AP C24 and CER NS C24 using the CHARMM36-Wang force field that CER AP has a larger APL than CER NS [132]. In this case, steric hindrance caused by the additional hydroxyls in the CER AP headgroup has a larger effect on packing than the increased opportunity for hydrogen bonding. In a more comprehensive study, Badhe et al. used the GROMOS-Notman force field to simulate CER AS, NdS, AdS, NP, AP, NH, and AH [126]; surprisingly results for CER NS were not reported. The CERs with an α-hydroxy fatty acid chain (i.e., CERs AS, AP, AH, and AdS) were found to have slightly larger APLs on average compared to CERs with a non-hydroxy fatty acid chain (i.e., CERs NP, NdS, and NH); ~39.7 Å^2^ and ~ 38.3 Å^2^, respectively. In addition, CERs with dihydrosphingosine (NdS and AdS) on average were found to have similar APLs to those with phytosphingosine (NP and AP) bases (i.e., 38.2 Å^2^ and 38.5 Å^2^ respectively), whereas CERs with 6-hydroxy sphingosine (NH and AH) bases have larger APLs on average (40.4 Å^2^) compared to CERs with dihydrosphingosine and phytosphingosine bases, which suggests that the addition of hydroxyl groups lower in the sphingosine chain more strongly affects the packing density.

Because variability in APL values from various studies can be large, it is best to compare APL or NLA values for systems with different lipid composition within a study, or between studies, that used the same force field and computational protocol. For example, the studies from Moore [115] and Wang [143] for pure CER NS C24 (Table 4) and for equimolar mixtures of CER NS:CHOL:FFA C24 (Tables 6), which is often used as a simplified model of more realistic compositions of SPP forming systems meet this requirement. In these studies, the addition of equal numbers of CHOL and FFA C24 molecules to CER NS C24 significantly decreased the APL (by 7 Å^2^ in Moore and 10 Å^2^ in Wang) with only small changes in NLA (0.1 Å^2^ increase in Moore and 1.3 Å^2^ decrease in Wang), suggesting that the packing densities of the mixture and pure CER systems were similar.

Moore et al. [115] and Wang and Klauda [134,143,148] each generated simulation results of bilayers with other lipid compositions (i. e., CER NS C16, CER NS C24, and CER AP C24 alone or mixed with different amounts of CHOL and FFA C24) that can be compared; see Tables S6 and S7 in the Supplementary Information. It is evident from the CER NS results that adding CHOL to CER bilayers causes a minimal change in the APL [115], which is expected given that CER and CHOL have similar cross-sectional areas [164]. Also, as expected, the addition of FFA, which has a single tail, to either pure CER or CER-CHOL mixtures decreases the APL significantly with almost no effect on the NLA (Tables S6 and S7). As observed for pure CERs, changes in the CER NS acyl tail length from C16 to C24 in mixtures with CHOL or with both CHOL and FFA C24 have no effect on the APL (Tables S6 and S7). Consistent with these results, Paloncyova et al. [152] observed in a study of equimolar mixtures of CER NS:CHOL:FFA C24 with CER acyl tail length varying from C2 to C24 that, like their study of pure CER bilayers, APL remained relatively constant when the CER acyl tail was longer than C16. Interestingly, the larger APL value observed for pure CER AP C24 compared with pure CER NS C24 disappears when each CER is mixed with CHOL and FFA C24 at mole ratios of 1:0.5:0.5 (34.5 Å^2^ for both) and 1:1:1 (32.6 Å^2^ and 32.8 Å^2^ for AP and NS, respectively) [148] (Table S6), suggesting that CHOL and FFA mitigate the steric hindrance caused by the additional hydroxyls in the CER AP headgroups. However, when considering simulations of SC lipid mixtures containing CHOL, it is important to remember that in experiments with equimolar mixtures of CERs, CHOL, and FFAs, CHOL often phase separates from the SPP [8,44]. Because phase separation in the lipid domain is difficult to observe in molecular simulations of gel phase membranes, due to the relatively small length and timescales of simulated systems, simulations of equimolar mixtures of CERs, CHOL and FFAs may include more CHOL than the experimental composition of the SPP.

#### 3.2.2. Density profiles

**Fig. 6.**
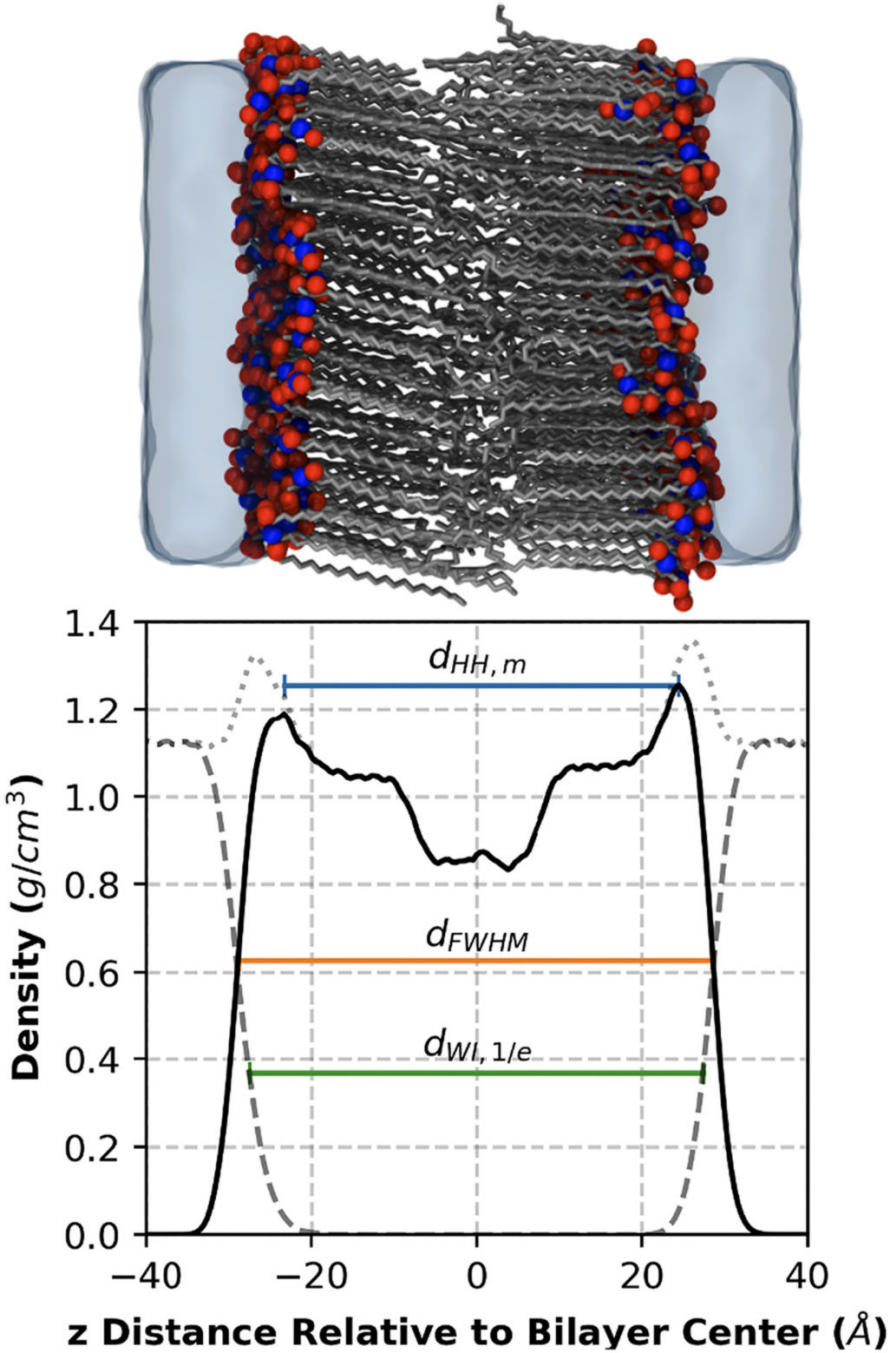
Total (dotted line), lipid (solid line), and water (dashed line) mass density profiles for a pure CER NS C24 bilayer simulation from Moore et al. [115] (snapshot on top) showing the bilayer thickness calculated using the *d_HH,m_* (48.5 Å, calculated from the lipid mass density profile), *d_FWHM_* (57.3 Å) and *d*_*W*,1/*e*_ (55.0 Å) methods. Values obtained using methods that do not use density profiles are *d_V_* = 56.0 Å, *d_REF_* (O & N) = 51.9 Å, and *d_REF_* (O) = 52.0 Å.

Density profiles are another structural property that is easily calculated from a simulation trajectory and relevant to the lamellar organization of a bilayer. Density profiles are constructed by creating a histogram of the atomic positions in the direction normal to the plane of the bilayer, where each point in the profile is normalized by the volume of the bin it represents. The atomic positions can be weighted by select atomic properties to give certain profiles. For example, in mass density profiles the position of each atom is weighted by its mass; see Fig. 6 for mass density profiles of all atoms (total), lipids, and water in a pure CER NS C24 bilayer.

Weighting by neutron scattering length yields a neutron scattering length density (NSLD) profile [115,129], whereas weighting by the number of electrons per atom gives an electron density profile. Electron and NSLD profiles are typically only calculated to compare with experimental data. These profiles weight polar moieties, such as those found in the lipid headgroup region, more than in the mass density profiles, which results in sharper peaks at the headgroup region. The localization of specific species and/or groups (e.g., CER headgroup) can be represented by component-specific density profiles. For example, in mixed lipid systems, comparisons of mass density profiles for all lipids and for CHOL have revealed that CHOL tends to sit away from the bilayer–water interface [115,154,161], consistent with experimental NSLD profiles [26]. Mass density profiles have also revealed that the region at the middle of the bilayers, occupied by the lipid tails, is less dense than just inside the lipid headgroups [115,154,161], and that the behavior in this region is dictated by the lipid composition. Both Moore et al. [115] and Wang and Klauda [143] calculated the NSLD profile of an equimolar CER NS:CHOL:FFA C24 bilayer, and found good agreement with the profile from a similar experimental system [26], providing evidence that the simulated systems are good representations of the SPP found in model SC membranes [115].

#### 3.2.3. Bilayer thickness

Bilayer thickness is an intuitive metric of bilayer organization that can be compared with experimental repeat distances measured by neutron scattering and X-ray diffraction, recognizing that the experimental repeat distance includes water in the headgroup region, which bilayer thickness generally does not. For SC lipid bilayers, water contributions to the repeat distance differences are nearly always small, which is not the case for phospholipid bilayers. Compared with metrics like APL, bilayer thickness is more complicated because it can be calculated by different methods that yield different numerical values [134]. Examples based on mass density profiles are illustrated in Fig. 6. It is important, therefore, to specify the method of calculation when reporting bilayer thicknesses derived from simulation including bin size, which affects resolution. Note also that the bilayer thickness and orientation, or tilt, of the lipid tails are linked; for the same tail length, the bilayer thickness is naturally smaller for tails with larger tilt angles. Thus, it is useful to report tilt angle along with bilayer thickness.

An obvious method for estimating bilayer thickness is to calculate the distance between the headgroup peaks (*d*_*HH*_) in a density profile along the bilayer normal. Fig. 6 illustrates *d*_*HH,m*_ calculated from the lipid mass density profile. The electron density profile is also used (*d*_*HH,e*_). An advantage of the *d*_*HH*_ method is that this bilayer thickness is more comparable than the other methods illustrated in Fig. 6 to repeat distances derived from X-ray diffraction and NSLD experiments. For the most direct comparability to repeat distance from NSLD experiments, bilayer thickness should be calculated from simulated NSLD results (*d*_*HH,n*_). The *d*_*HH*_ method has the disadvantage of being unsuitable for some multicomponent systems where there may be no clear headgroup peak or where there are multiple peaks from the headgroup locations of the different components. Additionally, care must be taken in comparing *d*_*HH*_ results for bilayers with headgroup-headgroup interfaces as compared to bilayers with headgroup-water interfaces; systems with headgroup-headgroup interfaces may exhibit only a single broad peak in this regime, resulting in a larger *d*_*HH*_ value than that calculated for otherwise identical systems with headgroup-water interfaces [110].

The bilayer thickness, designated as the full-width half-maximum thickness (*d*_*FWHM*_), is the distance between half of the maximum peak values in a lipid density profile as illustrated in Fig. 6 for the mass density profile [162]. An advantage of this method is that it does not require the presence of a well-defined headgroup peak. Another approach is to calculate the distance between the lipid–water interfaces on either side of the bilayer, where the interface is defined as the location at which the water density (mass or electron) along the bilayer normal direction falls to 1/*x* multiplied by its bulk value where *x* is typically equal to e (*d*_*WI*,1/*e*_) or 2 (*d*_*WI*,1/2_); Fig. 6 shows *d*_*WI*,1/*e*_. While this metric can be used for multilayer systems by normalizing by the number of leaflets considered, it would not be able to identify layer-by-layer variation that could arise due to differing hydration levels or different lipids compositions or structure.

There are also a few methods that do not use density profiles. One of these is the reference atom method (*d*_*REF*_), in which the bilayer thickness is the distance between specific reference headgroup atoms in each leaflet. Oxygen and nitrogen atoms of lipid molecules are commonly used as reference atoms for SC lipid systems as noted in Tables 5 and 6. The choice of reference atoms affects the value of the bilayer thickness, making comparisons of results that used different reference atoms questionable. Like measurements using the various *d*_*HH*_ methods, calculated *d*_*REF*_ results will differ for hydrated and dehydrated bilayers due to the presence of a headgroup-water interface in the hydrated bilayers, as discussed above. A second method involves calculating the bilayer thickness (*d*_*V*_) from the total lipid volume. In this method the water volume is subtracted from the total volume of the simulation box containing the bilayer(s), which is divided by the cross-sectional area of the simulation box and the total number of bilayers, where the water volume is calculated from the number of water molecules multiplied by an assumed volume per water molecule (e.g., assuming a density of 1 g/cm^3^ calculated from the density of the bulk water phase in the simulation [158]). MacDermaid et al. [159] calculated *d*_*V*_ by subtracting the average water thickness, calculated by dividing the water volume by the cross-sectional area, from the height (volume/area) of the simulation box. The *d*_*V*_ method assumes a constant water density across the simulation box, which may not hold at the lipid–water interface. Also, like *d*_*WI*_, it requires a bilayer-water interface and will fail to capture layer-by-layer differences that may exist in multilayer systems.

Bilayer thicknesses reported for pure CER NS vary among the studies by about 15 Å from 41.0 to 57.5 Å for CER NS C24 (Table 4) and from 35.0 to 47.0 Å for CER NS C16 (Table 5). For comparison, the experimental repeat distances, determined by X-ray diffraction, for stable bilayers (either anhydrous or fully hydrated) measured at 299 K varied by approximately 10 Å from 42 Å for pure CER NS C16 [165] to 52–53 Å for pure CER NS with a mixture of primarily C18, C20, and C24 acyl chains [166]. (However, metastable phases were also observed, which had repeat distances of 47 Å for the fully hydrated CER NS C16 [165] and 56 Å and 59 Å for the anhydrous and fully hydrated CER NS/FFA mixture [166]; anhydrous CER NS C16 did not exhibit a metastable phase.) However, monolayers of CER NS in a V-shaped conformation can also occur as observed by Rerek et al. [167] for CERs NS and NP using FTIR (and perhaps not detectable in the X-ray diffraction experiments [165,166]). CER NP is known to form six solid phases: five are monolayers of the V-shaped conformation with different tilt angles between the hydrocarbon chains, and one is a bilayer arrangement with parallel hydrocarbon chains [136]. CER NS is likely to exhibit complex phase behavior similar to CER NP.

The simulation results depend on both the method of calculating the thickness and the force field. For example, for pure CER NS C24 bilayers (Table 4), bilayer thicknesses calculated using the *d*_*V*_ and *d*_*WI*,1/*e*_ methods yield results that are on average larger (by ~5.5 Å) than those calculated using the *d*_*HH,e*_ method, and thicknesses from GROMOS-based force field simulations are slightly higher on average compared with CHARMM-based force fields (56.8 and 54.6 Å, respectively for *d*_*V*_). Even when bilayer thicknesses are calculated from the same simulation, results from the *d*_*FWHM*_ and *d*_*HH,m*_ methods for a pure CER NS C24 bilayer differed by almost 10 Å (Fig. 6). The *d*_*WI*_ and *d*_*FWHM*_ methods might be expected to predict larger bilayer thicknesses than the *d*_*HH,e*_ method, as observed in Fig. 6, because the lipid-water interfacial region is entirely ignored by the *d*_*HH,e*_ method. In contrast, Wang and Klauda [143,148], using electron density profiles, report smaller bilayer thicknesses calculated using the *d*_*FWHM*_ method compared with the *d*_*HH,e*_ method for pure CER NS C24 and pure CER NS C16. However, comparisons of the Wang and Klauda results for CER NS C24 with those presented in Fig. 6 reveal a significant difference in the *d*_*FWHM*_ values (45.1 Å and 57.3 Å, respectively) while the values are close for *d*_*HH*_ (50.5 Å and 48.5 Å, respectively) and for *d*_*WI*_ (54.2 Å and 55.0 Å, respectively), suggesting the need for careful examination of the Wang and Klauda *d*_*FWHM*_ result. Notably, the relative order of the bilayer thickness method results for pure lipid systems could be different for mixtures of lipids, where water is able to penetrate into the headgroup region. When comparing bilayer thicknesses between publications, therefore, one must be cautious to account for the method used in calculating the reported values.

The bilayer thickness of a pure CER, unlike the APL, naturally depends on acyl tail length (cf. Tables 4 and 5). Paloncyova et al. observed a nearly linear increase in the volume per lipid (VPL) for pure CER NS bilayers as a function of the acyl chain length [152]. As a result, the bilayer thickness increases proportionally to the VPL for pure CER NS bilayers with acyl chain lengths longer than C12 because the APL is constant for those CERs [152]. In addition, CER NS with tail lengths longer than C16 interdigitate at the center of the bilayer [115,152]. Moore et al. found that the degree of interdigitation increased as the ratio of CER NS C24 to CER NS C16 increased in bilayer systems of only CER NS as well as in mixtures with CHOL and FFA C24 [115]. The addition of CHOL and FFA tend to have opposite effects on the bilayer thickness; CHOL generally decreases the bilayer thickness while FFA generally increases it [115,154,161]. For example, Gupta et al. found the *d*_*V*_ was 46.9 Å for an equimolar CER NS C24:CHOL bilayer and 59.3 Å for an equimolar CER NS C24:FFA C24 bilayer compared with 56.5 Å for the pure CER NS C24 system (Table 4) and 51.2 Å for the equimolar mixture of CER NS C24, CHOL, and FFA C24 (Table 6) [154]. For comparison, experimental repeat distances for the equimolar mixture of CER NS C24, CHOL, and FFA C24 with 5 wt% cholesterol sulfate at 305 K are 53.9 Å [81] and 53.4 Å [82].

#### 3.2.4. Tilt angle

The orientation of the lipid tails is typically described by the tilt angle with respect to the bilayer normal vector. Simulations of pure CER bilayers generally exhibit some degree of tilt, although this is often based on visual inspection and infrequently quantified. In addition, tilt angle is difficult to measure experimentally and has not been quantified using experimental techniques.

Tilt angle is typically calculated using one of two methods. In the first and simplest method, the angle is calculated between the vector formed by two reference atoms (i.e., a headgroup atom and the terminal tail atom) and the vector describing the bilayer normal (typically the z-direction in simulations). This method is unreliable if the lipids curl or are disordered in the central region of the membrane (i.e., the region where tails interdigitate); even if reference points are chosen such that this regime is avoided, the calculated value may still be strongly influenced by the chosen reference points and this approach may add a bias due to the underlying bond angles of the system. Additionally, this method can make comparisons across simulation resolutions challenging (i.e., simulations performed with atomistic compared to a coarse-grained models where the underlying atomistic positions are no longer known).

A more robust method of calculating the tilt angle is to determine the angle between the principal axis of rotation of the lipid tail and the bilayer normal [168]. Because this method relies on calculating the moment of inertia from a collection of atoms, the derived tilt angle tends to be less sensitive to the choice of bounds (i.e., which chain atoms are included in the calculation) or the underlying bond-angles, than the method that relies on reference atoms. Nevertheless, the principal moment is most often calculated for a subset of the tail that excludes the atoms in the segment that tends to occupy the interdigitated regime, where lipid order is typically reduced. This method can be easily applied to all simulation resolutions, allowing for direct comparisons between atomistic and coarse-grained models.

Lipids in a bilayer will tilt to decrease the spacing between the chains when the spacing dictated by the headgroups places the tails farther apart than their “optimal spacing” [169]. Because SC lipid headgroups are smaller than phospholipid headgroups, gel-phase SC lipid simulations tend to exhibit smaller tilt angles than gel-phase phospholipid systems [116]. Pure CER NS C24 bilayers exhibit tilt angles on the order of 15° (Table 4). In addition, GROMOS-based force fields result in lower tilt angles in comparison to CHARMM-based force fields (18° and 14°, respectively for CHARMM-based and GROMOS-based CER NS C24 bilayers). This difference may be expected given that tails in the GROMOS-based force fields do not have explicit hydrogen atoms, which may alter the tail packing. Additional components (CHOL and/or FFA) seem to decrease the tilt angle compared with pure CER bilayers (Table 6), which may be due to the disrupted lipid packing in the headgroup region allowing tails to be closer to one another without tilting [152].

#### 3.2.5. Interdigitation

Interdigitation is a measure of the overlap distance ($\lambda_{\text{ov}}$) of opposing lipid tails in the hydrophobic region between two adjacent leaflets. It is calculated by integration of the overlap in the lipid mass density profiles of adjacent leaflets 1 and 2 using Eq. (1) [161]:

(1) $$\lambda_{ov}=4\int_{z_{\text{min}}}^{z_{\max}}\frac{\rho_{1}(z)\rho_{2}(z)}{(\rho_{1}(z)+\rho_{2}(z)\;{)}^{2}}dz$$

where $\rho_{1}(z)$ and $\rho_{2}(z)$ are the lipid mass density profiles for each leaflet along the bilayer normal (z), and $z_{min}$ and $z_{max}$ are the minimum and maximum z-coordinates of the simulation box (or positions outside regions of nonzero density [134]). All lipids that could be part of each of the bilayer leaflets (i.e., lipids with headgroups at the outer boundary of or inside each leaflet) should be included in the calculation; lipids with tails pointing out rather than into the chosen bilayer will not affect the calculation because the product of $\rho_{1}(z)$ and $\rho_{2}(z)$ for these lipids will be zero.

In mixed lipid systems, interdigitation values are often calculated for individual lipid components rather than for all components combined. For example, Wang and Klauda [148] calculated interdigitation for each component in ternary mixtures of CHOL and FFA C24 mixed with either CER NS C24 or CER AP C24, and Das et al. [161] and Podewitz et al. [145,146] calculated interdigitation for only CER NS C24 in mixtures with CHOL and FFA C24. Interdigitation values for all lipid components of the mixture combined should be similar to that of the component with the largest interdigitation or the average of components with larger and similar interdigitation. Interdigitation of all lipids might exceed that of individual lipid components if interdigitation between different lipid components (e.g., CER tails with FFA tails) is larger than interdigitation between the same lipid component. Thus, for the equimolar mixture of CER NS C24, CHOL and FFA C24 (fully protonated) at 305 K, an overall interdigitation of 10.5 Å from CG simulation [110] agrees well with FFA C24 (10.6 Å), which was the component with the largest interdigitation in atomistic simulation from Wang and Klauda [148] (interdigitation values for CER and CHOL were 6.4 Å and 0.88 Å, respectively). Reanalysis of trajectories of Moore et al. [115] for the same system reveal similar values to Wang and Klauda [148] with 7.3 Å, 0.1 Å, and 8.7 Å, respectively for CER, CHOL, and FFA, and an overall interdigitation of 8.1 Å between all lipid components. In a double bilayer system containing pure CER EOS, Wang and Klauda [134] calculated interdigitation of the very long CER EOS fatty acid tail by defining $\rho_{1}(z)$ and $\rho_{2}(z)$ as the lipid mass density profiles for the adjacent bilayers instead of leaflets because the fatty acid tail always penetrated through the adjacent interior leaflet. A similar approach could be used for assessing interdigitation of EO-type CER components in mixed lipid systems that produce an LPP.

#### 3.2.6. Order parameters

Several order parameters are commonly calculated from simulations of lipid membranes [170-173], including the carbon-hydrogen order parameter ($S_{CH}$), the carbon-carbon backbone order parameter ($S_{CC}$), and the nematic order parameter ($S_{2}$). While each of these provides different information and requires slightly different inputs, they can all be calculated using the same general equation:

(2) $$S=\frac{3}{2}\langle cos^{2}\theta \rangle -\frac{1}{2}$$

where ⟨⟩ designates the ensemble average. $S_{CH}$ quantifies the orientation of the C─H bonds with respect to the bilayer normal (i.e., $\theta =\theta_{z}$). Typically, this is restricted to the saturated carbon atoms of the lipid tails and excludes the terminal methyl group. Values of $S_{CH}$ range from −0.5 to 1, where $S_{CH}=-0.5$ represents a lipid chain entirely in a trans configuration with the C─H bonds oriented perpendicular to the bilayer normal (i.e., $\theta_{z}=90^{{}_{{}^{{}^{\circ}}}}$ when the chain backbone is oriented parallel to the bilayer normal) and a value of $S_{CH}=1$ indicates that the C─H bonds are parallel to the bilayer normal [173]. $S_{CH}$ can be directly compared to experimental values of the carbon-deuterium order parameter, $S_{CD}$, which has been used to validate atomistic force field parameters [117,132], although often the absolute value of $S_{CH}$ is reported, in order to compare the data available from experiment. For example, Wang and Klauda calculated the $S_{CH}$ parameters for an equimolar mixture of CER NS C24: CHOL:FFA C24 at 65 and 80 °C using the CHARMM-Wang force field and found good agreement between these and experimental $S_{CD}$ values [143].

$S_{CH}$ cannot be directly calculated for united atom or coarse-grained models because hydrogen atoms are not explicitly included. Instead of $S_{CH}$, the related metric $S_{CC}$, which quantifies the orientation of the carbon backbone with respect to the bilayer normal, is calculated using Eq. (2) [172,173]. In this case, the vector between carbons *i*-1 and *i* + 1 is typically used to capture the backbone orientation of carbon *i*, which is then used to calculate the angle with respect to the bilayer normal (i. e., $\theta =\theta_{z}$). Note that calculating $S_{CC}$ between carbons *i* and *i*-1 (or *i* and *i* + 1) instead will cause odd/even variations due to bond angles. The range of values of $S_{CC}$ is −0.5 to 1.0, where $S_{CC}=1$ when the chain backbone is oriented parallel to the bilayer normal ($\theta_{z}=0^{{}_{{}^{{}^{\circ}}}}$), and $S_{CC}=-0.5$ when it is oriented perpendicular to the bilayer normal ($\theta_{z}=90^{{}_{{}^{{}^{\circ}}}}$).

**Fig. 7.**
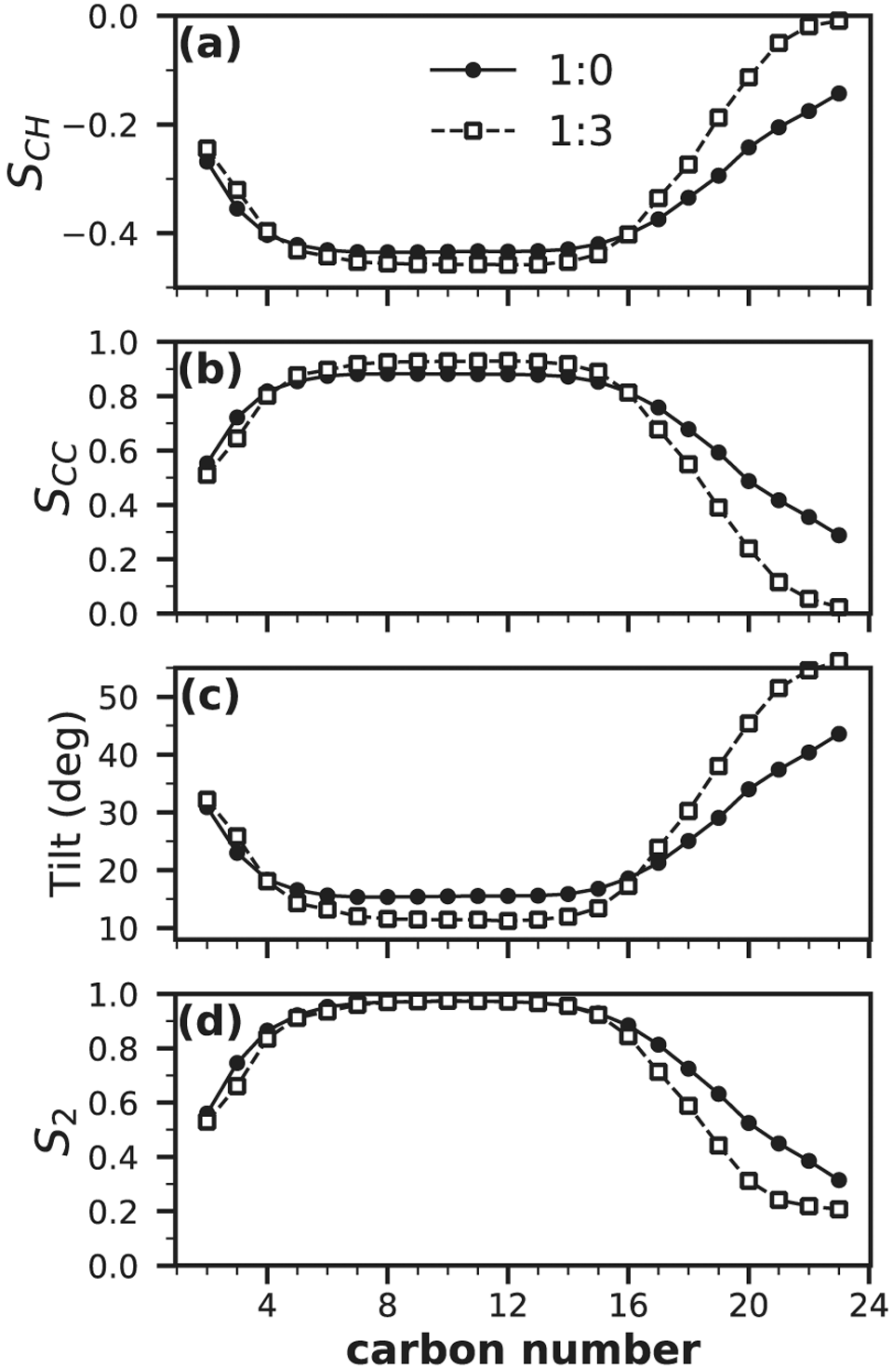
Comparisons of the *S_CH_*, *S_CC_*, mean tilt angle, and *S*_2_ order parameters for the fatty acid chain of CER NS C24 in bilayers of either pure CER NS C24 or a 1:3 molar ratio with CER NS C16 derived from simulation results reported by Moore et al. [115]. Carbon number 24 corresponds to the terminal methyl with structure CH_3_.

A complication of the $S_{CH}$ and $S_{CC}$ parameters is that they capture orientation (i.e., tilt angle) in addition to the order/disorder (i.e., uniformity) of the system. For example, $S_{CH}=0$ can mean that the system is isotropic (i.e., disordered), or that it is well ordered with C─H bonds aligned at 54.7° with respect to the bilayer normal [173]. Thus, comparison of numerical values between different systems must be done carefully, as both the tilt angle and disorder in the hydrocarbon chains independently affect the values of $S_{CH}$ and $S_{CC}$. This is illustrated by the comparison of $S_{CH}$ and $S_{CC}$ values for carbon atoms in the CER NS C24 fatty acid chains in bilayers of pure CER NS C24 (designated as 1:0) and a 1:3 mixture of CER NS C24 with CER NS C16 (Figs. 7a and b). Data for this analysis are from simulations with the CHARMM36-Guo force field reported by Moore et al. [115]. Both systems and metrics exhibit three different regimes with transitions roughly at carbon numbers 5 (nearest the headgroup) and 15 (end of the C16 fatty acid chain). In the central most regime, where values of the metrics remain roughly uniform along the chain backbone, the magnitude of $S_{CH}$ and $S_{CC}$ for the mixed system is slightly larger than for pure CER NS C24. This does not necessarily indicate that the mixed system is more ordered. In this example, the different numerical values in the order parameters are related to the small difference in tilt angle (~5°) between the two systems (Fig. 7c). These differences in $S_{CH}$ and $S_{CC}$ would be larger if the difference in the average tilt angles for the two systems were larger. We note that in the third regime (starting at carbon number 15), values of both $S_{CH}$ and $S_{CC}$ trend toward zero, which, when coupled with visual inspection, suggests that this variation is associated, primarily, with loss of uniform ordering rather than a uniform change in tilt angle in these systems.

The nematic order parameter ($S_{2}$) is a better choice for evaluating the uniformity of chain ordering independent of chain tilt angle. It, like $S_{CC}$, examines ordering in the chain backbone, but $S_{2}$ defines θ as the angle between an individual backbone vector and the average direction (i.e., director) of the backbone vectors in the leaflet, rather than the bilayer normal used in $S_{CC}$ [168]. As a result, $S_{2}$ can be used to meaningfully compare uniformity between different systems and different system resolutions (i.e., atomistic, united atom, and coarse-grained). A value of $S_{2}=1$ corresponds to completely uniform orientational ordering of the backbone vector; reduction in the value of $S_{2}$ directly corresponds to reduced uniformity of the orientational ordering with a value of zero representing an isotropic system. Calculations of $S_{2}$ should consider individual leaflets separately because each leaflet can have different tilt angles, and even in systems where leaflets have, on average, identical tilt angles, lipids may point in different directions (e.g., cross-tilted systems). Fig. 7d, shows $S_{2}$ plotted as a function of carbon number, where the vector describing the backbone of each atom is calculated in the same way as for $S_{CC}$ (i e., between *i*-1 and *i* + 1 for carbon *i*). The general trends with respect to carbon number are similar to that observed for $S_{CC}$ and $S_{CH}$ with transitions at carbon numbers 5 and 15. However, in the central regime, the degree of orientational ordering is nearly identical for the two systems, clearly demonstrating that differences in the numerical values for $S_{CH}$ and $S_{CC}$ in this regime (Fig. 7a and b) arise from differences in the system wide tilt angle.

Order parameters are sometimes reported as a single average value for the entire chain backbone rather than for each carbon. This is especially common for nematic order. Because order parameter values usually vary with carbon number, the method for calculating the average should be reported. For example, a simple method is to average the parameter values from the entire chain or from a subset of the backbone atoms (such as the well-ordered regime between carbons 5–15 for the systems presented in Fig. 7). For metrics such as $S_{2}$, the backbone orientation can be represented by the vector associated with the smallest moment of inertia of the backbone atoms (or subset of atoms) as discussed above in the context of tilt angle [168]. Using the moment of inertia allows molecules without long carbon tails, such as CHOL, to be described within a consistent framework for evaluation of backbone ordering. Average values of $S_{2}$ for SC lipids are typically high, in the range of 0.95–0.99; for example, in an equimolar mixture with FFA C24 and CHOL using the CHARMM36-Guo force field, $S_{2}$ is 0.99 for a bilayer of pure CER NS C24 and 0.96 for the mixture of CER NS C24 with CHOL and FFA [115].

Metrics other than $S_{CH}$, $S_{CC}$, and $S_{2}$ have been used to quantify the behavior of chain ordering in membranes. For example, as shown in Fig. 7c, tilt angle provides similar information to $S_{CC}$, which is not surprising given that $S_{CC}$ is calculated directly from the average tilt angle. Just as $S_{CC}$ or $S_{2}$ primarily differ in their choice of reference vector (i.e., the bilayer normal versus leaflet director, respectively) other choices of reference vector can be used to capture different behaviors, such as calculating the orientation between a given chain and its neighbors as a function of distance between the two chains to determine spatial correlations in the system [174]. To quantify the degree of in-plane ordering of the chains, the 2d hexagonal order parameter (often called the hexatic order parameter) of the projection of the center of mass of lipid tails is commonly calculated; changes in hexagonal ordering have been used to identify order-order and order-disorder phase-transitions for CER membranes [130]. Again, because simulations provide explicit knowledge of the positions of all molecules in the system through time, ad hoc (i.e., non-standard) metrics of lipid ordering can be devised to quantify and probe different aspects of lipid behavior. As with the aforementioned structural quantities, reporting these standard lipid tail order parameters provides additional data to allow for meaningful comparisons between different studies and force fields.

#### 3.2.7 Mechanical properties

Molecular dynamics can also be used to measure mechanical properties of model SC membranes. Primarily the area compressibility modulus, surface tension, and local pressures have been reported and can be used to elucidate the fluidity or rigidity of the system. Venable et al. provide an excellent explanation of how these values may be computed [175].

The area compressibility modulus ($K_{A}$) measures the energy required to change the lateral density of the membrane. It is the most common mechanical property extracted from simulations, largely due to its easy calculation from the variance in the fluctuations in the lateral area:

(3) $$K_{A}=\frac{k_{B}T\langle A\rangle }{\langle \delta A^{2}\rangle }$$

Here, 〈A〉 is the average total area, $\langle \delta A^{2}\rangle$ is the variance in the lateral area, $k_{B}$ is the Boltzmann constant, and T is the absolute temperature. Wang and Klauda determined $K_{A}$ was 2000 mN/m for pure CER NS C24 bilayers in simulations using the CHARMM36-Wang force field [134,143]. For reference, this value is an order of magnitude larger than those of gel-phase phospholipid bilayers, which typically have a $K_{A}$ of ~300 mN/m [176]. Tail length also affects $K_{A}$ for pure CER NS systems, where bilayers with C16 tails had a smaller $K_{A}$ compared with those with C24 tails [134,143]. This suggests that pure CER bilayers with C16 tails are more laterally deformable than those with C24 tails. In addition, Wang et al. observed that pure CER AP C24 bilayers have higher $K_{A}$ values than pure CER NS C24 bilayers, indicating that CER AP bilayers are more resistant to changes in the lateral area. This may be caused by the increase in hydrogen bonding in CER AP headgroups which rigidifies the headgroup region. Equimolar mixtures of CER NS C24:CHOL:FFA exhibit $K_{A}$ values roughly 1.5 times that of pure CER NS C24 bilayers [143,148,154,161]. As such, mixed systems require more energy to compress or expand laterally, which may contribute to the mechanical stiffness of the SC. The bending modulus is often calculated for phospholipid systems to measure the propensity for the bilayer to curve. However, Notman et al. asserted that this property cannot be measured for CER systems, which are much more rigid and do not appreciably undulate over the course of the simulation [122].

In studies of mixtures of CER NS C24, CHOL and FFA C24 with molar ratios of 1:1:1, 1:2:1, 1:0.5:0.5, 1:1:0.5, and 1:1:0.2, Das et al. found that increases in the CER and CHOL fraction causes a decrease in $K_{A}$ indicative of a more flexible bilayer [161]. In contrast, increases in FFA caused an increase in $K_{A}$ resulting in a more rigid bilayer [161]; this observation is consistent with experiments showing that FFA enhances orthorhombic phase formation albeit in systems with mixed CERs, diverse FFA chain lengths, and the presence of the LPP [14,20,103]. Das et al. also observed that CHOL promotes smaller bilayer thicknesses and increases the lateral density of lipid tails as evidenced by density profiles [161]. Experimentally, when CER EOS is absent from the SCS mixture of CERs and FFAs, orthorhombic phase formation only increases for small CHOL additions; for CHOL: CER mole ratios above 0.1 in an equimolar mixture of CER and FFA, the relative amounts of the orthorhombic and hexagonal phases remain essentially constant [44].

### 3.3. Observations from atomistic simulations

We now review observations in the literature derived from atomistic simulations of SC lipid systems. Such simulations have primarily focused on SC systems that mimic the SPP, though a limited number of studies attempting the LPP have also been reported.

#### 3.3.1. Lipid hydrogen bonding

All of the major lipid components of the SC contain functional groups in the headgroup that can participate in hydrogen bonds. Consequently, the orientation and packing of these headgroups are largely dictated by hydrogen bonding. Most often, a set of geometric criteria are used to determine hydrogen bonds. The criteria generally include the donor-acceptor distance, the acceptor-hydrogen distance, and the hydrogen-acceptor-donor angle. The radial distribution function between hydrogens and acceptors is another metric that can be used to quantify hydrogen bonding. Since simulation offers atomic-level resolution, it is simple to quantify and compare inter- versus intramolecular hydrogen bonding, as well as the hydrogen bonding between the different lipid species.

Early simulations conducted by Pandit and Scott [119] showed that inter-lipid hydrogen bonding is more prevalent in pure CER bilayers compared to pure sphingomyelin bilayers. Das and Olmsted studied H-bond network formation in pure CER bilayers and found that, although there are a high number of CER-CER hydrogen bonds, the relatively small headgroups form clusters of hydrogen bonds rather than a percolating network [265]. This was further demonstrated in the work of Guo et al. [130], where they noted that pure CER NP bilayers contained more hydrogen bonds than pure CER NS bilayers due to the additional hydrogen bond donor and acceptor in CER NP compared to CER NS [130]. This observation also suggests that the increased hydrogen bonding provides thermal stability to the bilayer, which is consistent with the higher gel-liquid phase transition temperature of pure CER NP bilayers compared with those of pure CER NS determined from the simulations [130]. In agreement with these simulations, experimental FTIR data of pure CERs NS and NP determined that CER NP participates in a greater number of hydrogen bonds and has a higher transition temperature than CER NS [153,167].

Several papers report a higher fraction of CER-water hydrogen bonds relative to CER-CER hydrogen bonds in pure CER NS bilayers regardless of tail length [130,138,154]. Furthermore, Papadimitriou et al. studied pure CER NS C24 bilayers with several different force fields (CHARMM36-Wang, GAFF, GROMOS97-54A7, OPLS-UA, and GROMOS-Papadimitriou), and found that the total number of hydrogen bonds was generally consistent, and also confirmed that lipid-water hydrogen bonds are generally more prevalent than lipid-lipid hydrogen bonds [138]. In contrast, however, Notman et al., reported that CER-CER hydrogen bonds are more abundant in a pure CER NS C24 bilayer compared to CER-water hydrogen bonds (2.8 and 0.3 hydrogen bonds per CER for CER-CER and CER-water interactions, respectively), which may be due to the slightly elevated temperature studied (i.e., 323 K compared to 300–305 K in other studies) [122]. Nonetheless, the publications of Notman et al. and several others agree that the CER-CER hydrogen bonds predominantly occur between hydroxyl groups rather than amide groups [122,130,132,143,148]. Guo et al. observed that the ratio of CER-CER versus CER-water hydrogen bonds shifts from 80/20 in CER NS C16 to 40/60 in pure CER NP C16 bilayers, to which the higher gel-liquid phase transition is attributed [130]. Similarly, Wang and Klauda reported an increase in hydrogen bonding for pure CER AP C16 bilayers compared to those of pure CER NS C16 [132,134].

For mixed lipid bilayers containing CER NS C24:CHOL:FFA C24, lipid-water hydrogen bonding has been found to again dominate over lipid-lipid hydrogen bonding [147,154]. CERs participate in more hydrogen bonds per molecule compared to FFA, and FFA forms more hydrogen bonds per molecule than CHOL [110,147,148,154,177]. This can be attributed to the number of hydrogen bond forming groups on each of the molecules. Both Moore et al. [115] and Wang et al. [143] found that hydrogen bonding is unaffected by the CER tail length when comparing bilayers composed of equimolar CER NS, CHOL, and FFA C24 with varying ratios of CER NS C16 to CER NS C24; this suggests that the headgroup interactions and packing are largely independent of tail properties [115,143]. Wang et al. [143] also observed that increasing the temperature of equimolar CER NS C16:CHOL:FFA C24 and CER NS C24:CHOL:FFA C24 bilayers from 305 to 353 K reduced the number of inter-lipid hydrogen bonds, indicating that elevated temperatures may reduce SC barrier function by disrupting the hydrogen bond network between lipids. It is important to remember that the simulations were performed on bilayers, rather than multilayers, and that experimental systems with mismatched tail lengths often exhibit phase separation, which would not be observed in these simulations [33,76,83].

In more realistic SC lipid systems, the CER-water interface is much less relevant due to the low hydration in the skin [8]. Das et al. [177] found that in the inner leaflets of a double bilayer system (Fig. 5c) of CER NS C24:CHOL:FFA in a 2:2:1 molar ratio, a significant portion (42%) of the hydrogen bonds are between lipids in adjacent leaflets, providing adhesion between the inner layers. Similarly, Del Regno and Notman [147] observed that inter-leaflet hydrogen bonds make up roughly 40% of total lipid-lipid hydrogen bonds for CER NS C24:CHOL: FFA C24 bilayers in equimolar and 2:2:1 ratios with low hydration (2 waters per lipid) levels. They also observed that water formed disconnected flattened pools between adjacent bilayers at this level of hydration and lipid-water hydrogen bonds made up roughly 60% of the lipid hydrogen bonds [147]. However, one should note that pools of water have not generally been observed experimentally within the lamella in healthy SC.

#### 3.3.2. Effect of temperature on lipid phase behavior

The phase behavior of SC lipids is clearly dependent on its composition and temperature. Simulations can examine the effects of temperature on the structure of SC membranes by slowly annealing and cooling the system over a large temperature range and sampling structural properties at various points in the simulation. Phase transitions are indicated by a rapid change in chain packing (i.e., APL) or chain ordering (i.e., $S_{CD}$ or $S_{2}$) over a small temperature interval. With this, the thermal stability can be assessed by comparing phase transition temperatures for systems of varying chemistry. However, there is generally a large hysteresis in the phase transition temperatures due to the relatively short timescales of molecular simulations, especially if the system does not reach an equilibrium state at each temperature state point [130]. In addition, reported experimental phase transition temperatures, especially for mixed lipid systems, often have large error bars, which make comparisons of experimental and simulated phase diagrams difficult. Nonetheless, several publications have reported reasonable agreement between simulated and experimental phase behavior.

Using the GROMOS-Notman force field, Notman et al. [122] simulated the thermal stability of a pure CER NS C24 bilayer at 283, 323 and 363 K. A gel-like phase was observed at 282 K and 323 K, but a liquid-crystalline phase was observed at 363 K, which suggests that a gel-liquid phase transition occurs between 323 and 363 K. However, using the same force field in a thermotropic study of bilayer phase behavior that examined the APL over a temperature range of 305–430 K, Guo et al. [130] observed an order-disorder phase transition for a pure CER NS C16 bilayer at 420 K. Similarly, Gupta et al. [154] reported an order-disorder transition temperature >400 K for a pure CER-C24 bilayer over the temperature range of 300–450 K, again using the GROMOS-Notman force field.

When using the CHARMM36-Guo force field, Guo et al. observed an order-disorder transition for a pure CER NS C16 bilayer at ~380 K by measuring the APL over a temperature range of 305–430 K [130]. Similarly, Paloncyova et al. [152] reported that pure CER NS C24 bilayers undergo an order-disorder phase transition at roughly 365 K using the CHARMM36-Wang force field based on changes in the APL over a temperature range of 300–400 K. As reference, experimental data based on Fourier transform infrared (FTIR) spectroscopy and differential scanning calorimetry (DSC) report a main order-disorder transition at 366 K and a solid-solid (i.e., orthorhombic to hexagonal) phase transition at ~340–345 K for CER NS with fatty acid chains of C16 to C20 [178]. The solid-solid transition is much more subtle, but was detected by Guo et al. [130] who observed small peaks in the heat capacity at 348 and 358 K using the CHARMM36-Guo force field, suggesting an order-order phase transition. It is evident from these results that overall the CHARMM-based force fields better predict the thermal phase behavior of pure CER NS compared to GROMOS-based force fields, which appear to be too stable and require much higher temperatures to undergo a phase change. One would expect similar concerns for other CERs and mixed lipid systems.

Calorimetric measurements from simulations of mixtures of CER NS, CHOL, and FFA have also been reported. Using the GROMOS-Notman force field, Gupta et al. [154] found that the addition of FFA C24 to pure CER NS C24 bilayers caused a decrease in the orderdisorder phase transition temperature, while the addition of CHOL to pure CER NS C24 and to equimolar CER NS C24:FFA C24 mixtures greatly increases the order-disorder phase transition temperature compared to that for pure CER NS C24. These results suggest that CHOL provides thermal stability to SC lipid lamella. Similarly, Paloncyova et al. [152] did not observe the gel to fluid phase transition in bilayers containing CER NS C24, CHOL, and FFA C24 in an equimolar ratio over a temperature range of 300–400 K using the CHARMM-Wang force field, which demonstrates the higher thermal stability of mixed lipid bilayers compared to the pure CER NS C24 systems. These simulation results are consistent with experiments of CER NS C18, CHOL and FFA C18, except for the equimolar ternary mixture, which exhibits a lower gel to fluid phase transition temperature than for pure CER NS C18 [179]; similarly, the measured gel-fluid phase transition temperature of ~340 K for the equimolar CER NS C24, CHOL and FFA C24 mixture [76] is below the ~365 K phase transition of pure CER NS [154].

#### 3.3.3. CER EOS and the long periodicity phase

The vast majority of publications describing molecular simulations of SC lipid mixtures have studied the SPP, which forms without the LPP in the absence of EO-type CERs (see Section 2.1). Since the arrangement of the lipid molecules in the ~13 nm LPP is uncertain, it is difficult to set up and run accurate pre-assembled LPP systems. Nonetheless, some papers in the literature have attempted simulation studies of membrane systems containing CER EOS, some of which exhibit an LPP-like structure.

In 2011, Engelbrecht et al. [129] performed simulations and experiments of a novel branched CER EOS molecule (the linoleic acid esterified in the ω-position is replaced with C10-methyl-branched palmitic acid) mixed with CER AP C18, CHOL, and FFA C22 in a 0.60:0.40:2.1:2.4 molar ratio. Neutron scattering experiments of this system detected a lamellar repeat distance of 4.8 nm and no LPP, consistent with previous experiments of systems with CERs EOS and AP [9]. They attributed the absence of the LPP to the strong headgroup interactions of CER AP, which restrict CER EOS to the SPP. An alternative explanation is that formation of the LPP is less favorable for EOS with the saturated branched chain compared with EOS linked to the unsaturated and more flexible linoleic acid [92,180,181]. The simulations were performed on a fully dehydrated stack of four pre-assembled bilayers of the same composition as the experimental mixtures. CERs AP and EOS were both placed in the hairpin conformation and the long acyl tail of the branched CER EOS extended through the bilayer with the palmitoyl chain in the adjacent bilayer. This arrangement remained stable after annealing to 363 K and cooling to 305 K over the course of 517 ns, except for two of the 80 branched CER EOS molecules in the simulation, for which the palmitoyl chain folded back at the ester linkage into the same bilayer as the rest of the molecule. The initial and final configurations had no LPP, like the experiments, and a final repeat distance of 4.0 nm, which is shorter than in the experiments. Although the repeat distance of the initial structure was not reported in the publication, the authors mention that the repeat distance rapidly decreased early in the simulation, which suggests that the initial structure may not have been stable. In subsequent experimental studies of this same lipid mixture that were prepared differently (spread onto the quartz substrate using a syringe instead of an airbrush pistol), a small fraction of lipids formed a LPP-like phase (~11.8 nm) along with the 4.8-nm SPP [182]; this LPP phase was absent when isopropyl myristate (10%) was added to the system [180].

In an early study of SC lipids, Das et al. [125] simulated mixtures of CERs EOS, NS, and NP in a 1:5:4 molar ratio mixed with CHOL and FFAs in 1:1:1 and 2:2:1 (CER:CHOL:FFA) molar ratios. A distribution of tail lengths from C20 to C34 were used for the acyl and free fatty acid tails. Simulations in which the molecules were placed randomly with random orientations in the simulation box along with 30 wt% water formed inverse micellar structures over the course of 50 ns. These were the first published simulations that attempted to self-assemble SC lipid membranes, and the only publication to attempt to do so using a UA force field (GROMOS-Das). In a subsequent simulation, CERs EOS, NS, and NP, mixed with CHOL and FFAs in a (0.1:0.5:0.4):1:1 molar ratio were arranged randomly with CERs in the hairpin conformation in a pre-assembled double bilayer structure in which each bilayer is separated by a 1 nm thick slab of water [125]. This arrangement also resulted in a micellar structure at about 90 ns that remained stable for the rest of the 260-ns simulation. The very high level of hydration, which is not present in experimental SC lipid membranes, may be responsible for the formation of micellar structures. An additional arrangement was simulated in which CERs EOS, NS, NP, CHOL and FFAs, again in a (0.1:0.5:0.4):1:1 molar ratio, were randomly placed between rigid “corneocyte walls” located at the top and bottom of a 28.8 nm tall simulation box [125]. The corneocyte wall was made up of a pre-assembled layer of hypothetical molecules formed by joining two CER NS molecules such that the sphingosine chains of each lipid are connected to the acyl chains of the other. As such, a CER NS headgroup is exposed to both the top and bottom of the interior lipids. The authors argue that lipids covalently bonded to the corneocyte envelope may promote the formation of a lamellar structure. However, after 1 μs of simulation, the system still did not form a lamellar structure. In experiments, SC models are constructed on substrate surfaces without corneocyte envelopes suggesting that the specific chemistry of the corneocyte cell envelope is not necessary for the formation of native SC lipid lamellar arrangement.

In 2018, Lundborg et al. [32] used the CHARMM36-Lundborg force field to simulate several lipid systems containing CER EOS in the “splayed bilayer” (i.e., extended) arrangement proposed by Iwai et al. (based on an equimolar mixture of CER NP C24, CHOL and FFA C24 without CER EOS), which assumes CHOL is associated with the sphingoid chain of the fully extended CER [31]. Simulations that included CER EOS were performed on mixtures with CERs NP and NS C24, with CHOL and FFA, in which both the FFA and CER NP acyl chains had a distribution of chain lengths from C20 to C30 [32]. Content of CER EOS, total CER, CHOL and FFA, the ratio of CER NP and NS, the arrangement of CHOL with the sphingoid and acyl chains, and water content were all “screened” for matches with the electron density observations from the cryo-TEM images of SC samples. Overall, the optimal similarity between the simulated and original cryo-TEM images was judged to be for the CERs EOS, NP, NS mixture with CHOL and FFA in a (0.15:0.68:0.17):1:1 molar ratio with 75% of the CHOL associated with the sphingoid chains and 0.3 water molecules per lipid. Good agreement with the periodicity of the experimental cryo-TEM data using the “splayed bilayer” model, in which all CERs adopt an extended configuration, was obtained although the bilayer thickness was only 10.6 nm. The discrepancy in bilayer thickness with the ~13 nm experimental repeat distance could not be explained, and suggests that the correct LPP structure still has not been obtained. Of the systems simulated by Lundborg et al. [32], only pure CER EOS exhibited a periodicity (13.0 nm) that was similar to the LPP, although quite different from the experimentally observed 9.3 nm repeat distance [183].

In 2019, Wang et al. studied a double bilayer of pure CER EOS initially arranged in the hairpin conformation with straight acyl chains that extended into the adjacent bilayer or the water layer adjacent to the outer leaflets; the lineolate tail of the latter folded back into the outer leaflet early in the simulation [134]. In two replicates the pure CER EOS systems remained ordered in double bilayers over 2.25 μs. However, in the third simulation of the same system, at about 1 μs the interdigitated interior layer of the membrane separated to form two disordered headgroup layers with a more ordered tail packing in the outer leaflets. The ordered bilayers of the first two simulations may be in a metastable state that would, with longer simulation times, transition to a disordered interior (the simulations were judged to have reached an equilibrated state when the APL stabilized). Dehydrated systems of CER EOS were not considered and the possibility of CER EOS adopting a fully extended conformation was not explored.

In a follow-up study, Wang et al. [135] simulated LPP systems by initializing their structures based on the molecular models proposed in Groen et al. [72] and Mojumdar et al. [29], in which an interior layer (slab) containing fully interdigitating tails is centered between two ordered (outer) bilayers constrained by layers of water (see Fig. 4b). Simulations were performed on systems containing 5 waters per lipid with approximately 5% of the water placed at each of the two bilayer-slab interfaces corresponding to ~1 water per lipid headgroup in the bilayer leaflet adjacent to the slab. They studied two systems, which contained approximately equimolar mixtures of CER, CHOL and FFA C24, where (based on the numbers in Table 1 of their paper) CER EOS was either 17 or 47 mol% of the total CER and the molar ratio of CER NS to NP was respectively 3:1 and 3.5:1. Notably, in experimental membranes with similar compositions, the latter composition forms only the LPP, whereas the former composition forms both the LPP and SPP [26]. After ~2–3 μs of simulation, the system with a lower CER EOS composition exhibited an ordered interior bilayer, whereas the system with a higher fraction of CER EOS had a disordered interior bilayer [135]. The increase in unsaturated linoleate tails in the central bilayer disrupted packing in the system with a higher EOS concentration. The system with a lower CER EOS fraction had a total bilayer thickness of ~13 nm whereas the system with higher EOS fraction had a thickness of ~ 11 nm. However, both models had similar but smaller thicknesses for the interior (slab) layer (approximately 3.4 and 3.2 nm respectively for the low and high CER EOS systems) compared to experimental electron density profiles of the LPP (4.0 to 4.2 nm) [29,72]. The terminal linoleate of the CER EOS acyl tails were found to continually fluctuate between folded (hooked) and unfolded conformations with transition times of only a few ns. Consistent with this observation, Mojumdar et al. [29] concluded from neutron diffraction experiments of the SCS that the terminal linoleate, which is located at the position of the inner headgroup and protruding into the inner lipid layer, must be able to fold back at least partly to fit in the gap between the ester bond of CER EOS and the terminal methyl groups of the FFAs and the extended acyl chains of the non-EOS CERs from the opposite headgroup region.

Simulations from Wang et al. [135] also showed that a large fraction (~32% and 43% for the low and high CER EOS systems, respectively) of CERs NS and NP in the inner leaflet of the outer bilayer were found to transition from the initial hairpin conformation to extended conformation over the course of the several microsecond simulations; times for this transition are on the order of 200 ns. This appearance of extended CERs agrees with experimental data which suggest that a large fraction of CER NS in the LPP is in an extended conformation [28,106]. However, Wang et al. only studied a single unit cell of a hydrated LPP membrane (i.e., outer bilayer-slab-outer bilayer). It is reasonable to expect that the repeat distance and fractions of extended CERs might be larger for dehydrated systems of multiple stacked LPP unit cells.

MacDermaid et al. [159] studied a mixture of CER EOS, CER NS C24, CHOL, and FFA C22 (0.5:0.5:1:1 molar ratio), which is nearly identical to a mixture that in experiments produced only the LPP with a 12.7 nm repeat distance [75] (0.5:0.5:1:1 molar ratio of CER EOS:CER NS C24: CHOL:FFA5 where FFA5, the five component FFA mixture listed in Table 2, has an average chain length of C22). The initial symmetric bilayer structure was prepared by replicating and rotating a monolayer containing an equal number of CER NS, CHOL, and FFA C22 molecules, after which half of the CER NS molecules were converted to CER EOS by adding atoms to the fatty acid chain and orienting the linoleic acid tails parallel to the monolayer plane. Simulation of this hydrated bilayer for ps at 1 bar and 303 K resulted in a disordered layer, approximately nm thick and mostly composed of the CER EOS linoleic acid tails with a small amount of CHOL, centered between two highly ordered leaflets; the overall thickness of the repeating unit (two leaflets + the disordered central layer) was ~5.4 nm, which is similar to the thickness of the SPP. A pre-assembled stack of four hydrated bilayers (with either 2:1 or 5:1 water molecules per lipid) did not change significantly during simulations at 303 K for ~0.5 μs. But when the bilayer stack was then heated for 0.25 μs to 368 K, well above the melting temperature of most CERs, leaflets of adjacent bilayers ‘fused’ at multiple points as water between the bilayers reorganized into droplets and continuous channels. This overall structure of hemifused bilayers, which contained some CER EOS in the extended conformation (CER NS conformation was not mentioned), separated by water droplets and/or channels was retained after annealing to 303 K for 1.8 μs. An LPP-like phase was never observed in their atomistic simulations.

MacDermaid et al. [159] also performed atomistic simulations on pre-assembled bilayers of CER EOS alone and mixed with 10, 20 or 30% CHOL; the starting configuration was not described. They found most of the linoleic acid segments of CER EOS, many of them in a hooked conformation, were located in the liquid-disordered layer that was sandwiched between two highly ordered leaflets containing the other parts of the CER EOS. CHOL was located in both the disordered and ordered layers. The bilayer thicknesses (*d*_*V*_) estimated from simulation were smaller than the repeat distances observed in experiment [183]; i.e., 8.2 nm compared with 9.3 nm^2^ for pure CER EOS, and 7.5 nm at 30% CHOL compared with 7.7 nm and 9.8 nm (two phases were observed) at 33% CHOL.

Although the various pre-assembled atomistic simulations involving CER EOS may show significant disagreement with experimental data, the results can provide some insight into the behavior of CER EOS and the possible structure of model LPP membranes. Accurately simulating systems with CER EOS is difficult because CER EOS is larger and less mobile than other SC lipids, and the systems simulated must be larger to accommodate formation of the LPP. Determining a good initial configuration for systems with CER EOS or other EO-type CERs and ensuring meaningful independence of the results from that configuration remains a significant challenge.

#### 3.3.4. Permeability and diffusivity

Permeant permeability, which is commonly used to evaluate the barrier effectiveness of skin and also SC lipid membranes, can be calculated using molecular simulations. Notman et al. [184] provide an excellent summary of permeability mechanisms through SC lipid bilayers. Because transport of molecules through a lipid bilayer occurs on timescales longer than those accessible by atomistic simulation, statistical mechanical relationships are used to estimate permeability. Specifically, permeability calculations from molecular simulation consider both thermodynamic and transport properties (free energy and diffusion values) of the system. These are generally related to the permeability coefficient using the inhomogeneous solubility-diffusion model [185] or via the time-fractional Smoluchowski eq. [186,187]. Most often the former approach, expressed by Eq. (4),

(4) $$k_{p}={\left(\int_{-h∕2}^{h∕2}\frac{exp(\;-\;\Delta G(z)∕k_{B}T)}{D(z)}dz\right)}^{-1}$$

has been used to estimate the permeability coefficient ($k_{p}$) for a permeant in SC lipid systems. In this equation h is the bilayer thickness, ΔG(z) and D(z) are the free energy of transfer and in-plane diffusion coefficient, respectively, at a position z normal to the bilayer plane, with the integration performed across the bilayer [185].

These quantities can be calculated using the “z-constraint” technique [185,188]. in which the center of mass of the chosen permeant molecule is constrained to fixed positions along the z-axis of the system (i.e., normal to the bilayer plane), while the permeant molecule remains free to move in the *xy* plane (i.e., tangent to the bilayer plane). The z-constraint method is convenient as it allows the simultaneous calculation of both ΔG(z), from the constraint force required to keep the center of mass of the permeant fixed at selected z locations, and D(z), from the in-plane diffusion of the permeant. Diffusion and free energy (representing permeant partitioning) can also be calculated separately, for example, to estimate parameters for the lipid layers in microscopic diffusion models that represent the lipid and corneocyte components of the SC separately. Although these measurements are generally taken in the direction perpendicular to the bilayer plane, the permeability (and diffusivity) can similarly be measured in the direction parallel to the bilayer; e.g., see supplementary information from Paloncyova et al. [189]. Unless noted otherwise, simulated $k_{p}$ values listed below are for transfer perpendicular to the bilayer plane. In general, these studies examine the influence of SC lipid composition and additives, such as chemical penetration enhancers (CPEs), on the transport of water or other molecules in lipid bilayers.

It is important to recognize some of the limitations of permeability (and diffusion) calculations from MD simulations. First, permeability (and diffusivity) calculated across the membrane normal direction does not take into account the lateral diffusion of a permeant within the lipid bilayer (i.e., parallel to the plane of the bilayer). This may be particularly important for hydrophobic permeants, which may, due to their limited solubility in the hydrophilic corneocytes, primarily permeate the SC through an extracellular pathway within the lipids rather than across the lipids and corneocytes in series as their hydrophilic counterparts may do [2,135]. Second, permeability calculated through a mixed lipid system via simulation does not consider inhomogeneity in the lipid membrane, such as phase separated domains and grain boundaries, which may play a major role in dictating the path of permeation. Third, most permeability (or diffusivity) calculations have been performed on a simulated bilayer with a water layer adjacent to the headgroups that, is absent or small in human, pig and mouse SC [99,100,190] and in SC lipid membranes [8,9]. Finally, simulated permeability cannot be directly compared to permeability coefficients measured through the skin or extracted SC lipids because the pathway can be confounded by the presence of corneocytes and proteins [2]. As a result, all comparisons of permeability coefficients estimated from MD simulations with experiments in skin necessarily involve assumptions, whether recognized or not, of the transport pathway through the skin. Despite these limitations, simulated permeability calculations in SC lipid systems can provide insight into the *relative* propensity of molecules to pass through the lipid matrix of the SC. A direct comparison to permeability measurements through SC lipid membranes would be possible if the lamellae in the experimental system are defect-free and aligned parallel to the supporting membrane, the number of bilayers is known, and resistance through the supporting membrane is insignificant. Because experimentally satisfying all these requirements is unlikely, permeability (and diffusion) estimates from simulation can at best only approximate experiments.

In 2009, Das et al. [266] computed water permeability through a variety of SC model bilayers. They observed that the calculated permeability for a pure CER NS C24 bilayer was approximately five orders of magnitude lower than that of DPPC bilayers. Adding CHOL increased permeability, due to a larger free volume and poor ordering. Paloncyova et al. [189] found comparable results when comparing the free energy barriers and diffusion coefficients of p-amino-benzoic acid (PABA) and the ethyl and butyl esters of PABA across bilayers of pure CER NS C24 and pure dioleoylphosphatidylcholine (DOPC) bilayers. The pure DOPC bilayers were more fluid and disordered, exhibiting greater diffusion coefficients (by ~10 fold) for the three PABAs combined with a lower free energy barrier (by ~1/3) for crossing the bilayer compared to the pure CER NS C24 bilayers [189].

Gupta et al. [156] observed in pure CER NS bilayers of different acyl chain lengths (from C8 to C24) that longer fatty acid chains reduced water permeability compared with shorter chains. This can be attributed to increased interdigitation for longer chain lengths, which increases the density in the bilayer center, as well as a larger bilayer thickness, which widens the free energy barrier. In a subsequent publication, Gupta et al. [191] compared the permeability of water and eleven other small (MW between 32 and 106) hydrophobic and hydrophilic (logarithm of the octanol-water partition coefficient between −2.11 and 3.27) molecules through equimolar CER NS C24:CHOL:FFA C24 bilayers. While the diffusion coefficients obtained were somewhat similar, hydrophilic permeants encountered the largest free energy barriers within the bilayer, while hydrophobic permeants encountered the largest free energy barriers around the headgroups. In general, the more hydrophilic permeants exhibited lower permeability coefficients compared to more hydrophobic permeants. Del Regno et al. [147] explored possible permeation pathways of water through a multilayer of equimolar CER NS C24, CHOL, and FFA C24. Lateral diffusion in addition to the permeability normal to the bilayer was measured, finding that lipids tend to penetrate the headgroups through cholesterol rich domains.

Wang et al. [134] simulated pure bilayers of CER NS C16 and CER NS C24. For each of these systems, the permeability coefficient of ethanol perpendicular to the bilayer plane was calculated. Compared with the CER NS C24 bilayer, the CER NS C16 bilayer exhibited a smaller free energy barrier through the headgroup region based on the potential of mean force (PMF) compared to pure CER NS C24 bilayers, which coincided with less interdigitation and higher free volume in the bilayer center for CER NS C16. Consequently, the average transverse permeability for the CER NS C16 bilayers was an order of magnitude larger than that of the CER NS C24 bilayer; i.e., $\log(k_{p},\;\text{cm}∕s)$ of −3.9 and −5.2, respectively.

Wang et al. [135] also estimated ethanol permeability coefficients for the two LPP systems described in Section 3.3.3, in which CER EOS was either 17 or 47 mol% of the total CER. Recall, that the initial LPP arrangement (an interior layer sandwiched between two identical bilayers with different compositions in their outer and inner leaflets) was retained after equilibration, but the system with more CER EOS formed a disordered interior layer, whereas the system containing less CER EOS formed an ordered interior layer. As a result, the system with less CER EOS had lower ethanol permeability (i.e., the transverse $\log(k_{p},\;\text{cm}∕s)$ was −4.7 compared with −5.7 for the system with more CER EOS). This is consistent with (although stronger than) the trend observed experimentally [75] for ethyl-PABA permeability in synthetic membranes composed of varying amounts of CER EOS mixed with CER NS in an equimolar mixture of CER:CHOL:FFA. In addition, both LPP model systems exhibited lower $k_{p}$ values than the SPP-type bilayers of either CER NS C16 or CER NS C24 in an equimolar mixture with CHOL and FFA C24. This indicates that the model LPP structure may be less permeable than the model SPP structures, which also agrees with the experimental trends observed for ethyl-PABA permeation through membranes of the SCS prepared with and without CER EOS [21,135].

Wang and Klauda [135] also compared the $k_{p}$ results with in vitro human skin permeability experiments by assuming transcellular transport (i.e., perpendicular diffusion) across a total of 80 lipid repeat units consisting of either the bilayer for the SPP-type systems without CER EOS or the bilayer-sandwiched interior layer for the LPP-type systems containing CER EOS; note that the different thicknesses of the SPP and LPP repeat units were not considered. In using this approach, Wang et al. have assumed that the SC can be described by a brick-and-mortar type model with 6 lipid repeat units (the mortar) separating 15 layers of corneocytes (the bricks), in which the corneocytes are highly permeable (i.e., they provide no resistance to mass transfer). This is similar to a published microscopic diffusion model [1]. which represents the SC with 105 lipid layers that are 13 nm thick and distributed evenly between, above and below a stack of 15 corneocytes with permeability relative to the lipid layers that depends on the size and lipophilicity of the permeant; in this microscopic diffusion model, ethanol permeability in the corneocyte is high relative to the transverse lipid permeation. With the adjustment for 80 lipid layers, Wang et al.’s calculated $\log(k_{p},\;\text{cm}∕s)$ is approximately −5.9 for the systems without EOS (except for pure CER NS C24, which is −7.1), and − 6.6 and − 7.6 respectively when more or less EOS is included. These latter three results are comparable to the range of experimental observations for ethanol permeation through human skin from water $\log(k_{p},\;\text{cm}∕s)=-6.65$ [192] to and − 7.08 [193]), although this agreement cannot prove the correctness of either the $k_{p}$ estimated from atomistic simulation or the assumed SC model.

Gajula et al. [194] also used molecular simulation combined with an SC model to predict permeability in human SC for three solutes: caffeine, fentanyl, and naphthol. However, different from Wang et al. [135], the corneocytes in the brick-and-mortar SC model used by Gajula et al. are impermeable and permeation is exclusively through the tortuous path of the lipid matrix that surrounds the corneocytes. Gajula et al. further assumed that the lipid matrix diffusion coefficient is equal to the transverse diffusion coefficient calculated for each solute from their bilayer simulations of an equimolar mixture of CER NS C24:CHOL:FFA C24, and they use experimental values of the SC-vehicle partition coefficient for the solute in the boundary condition of the lipid matrix with solute in the vehicle (i.e., the solution containing the solute) on the SC surface. They adopted the SC diffusion model from Kushner et al. [195], which represents two-dimensional solute diffusion through a lipid matrix surrounding impermeable corneocytes with a one-dimensional homogeneous solute diffusion model that accounts for the tortuous pathways of permeation limited to the lipid matrix with parameters that depend solely on the geometry of the chosen brick-and-mortar model. However, Gajula et al. [194] applied this one-dimensional model from Kushner et al. [195] to two-dimensions and then solved it for a brick- and-mortar configuration using a finite element method. As a result, the average SC diffusion coefficients used to calculate the cumulative solute release profiles that are compared with experiments (which are between roughly 1 × 10^−15^ and 4 × 10^−15^ m^2^ s^−1^ for the three solutes studied) are equal to the average diffusion coefficient calculated from molecular simulation (between 2 × 10^−10^ and 4 × 10^−10^ m^2^ s^−1^) adjusted twice for permeation limited to the tortuous pathways of the lipid matrix; see Section S4 in the Supplementary Information for additional details. Using SC diffusion coefficients estimated correctly by adjusting the lipid matrix diffusion coefficient only once gives values between 6 × 10^−13^ and 9 × 10^−13^ m^2^ s^−1^, which greatly over predicts the experimental observations (see Section S4).

Other authors have combined permeability or diffusion coefficients calculated from molecular simulation with experimental observations to predict $k_{p}$ of solutes that transfer into human skin from water. For example, Rocco et al. [196] developed an equation for predicting $k_{p}$ through human skin from a water vehicle based on molecular properties of the permeant in a simulated bilayer composed of CER EOS:CER NS C24:CHOL:FFA C24 with a molar ratio of 0.25:0.75:1:1 and arranged as proposed by Iwai et al. [31] (i.e., fully extended CER, CHOL aligned with the sphingoid chain of the CER, and FFA aligned with the acyl chain of the CER) (P. Rocco, personal communication, email 4 May 2021). The CHARMM36-Wang [132] force field and steered molecular dynamics were used to calculate average values of the molar volume (*MV*) and D in the direction perpendicular to the bilayer plane at 300 K in a 4 Å thick region near the water-lipid interface [196]. Separately, the Virtual LogP for each permeant was calculated by the molecular lipophilicity potential (MLP) approach [196]. The three coefficients in the chosen equation for predicting $k_{p}$ were derived by linear regression of the logarithm of experimental $k_{p}$ values for 80 different permeants to log(P×D∕MV) and the absolute temperature of the permeability experiment. The resulting equation showed slightly better agreement with the experimental $k_{p}$ data than that estimated from the popular Potts-Guy eq. [197] knowing only MW and the logarithm of the experimental octanol-water partition coefficient (logP), which are readily available for many chemicals [196,197]. The advantages, however, of calculating $k_{p}$ values for human skin using input parameters derived from molecular simulation when simpler non-simulation methods are available are not clear.

Using a different strategy (with some details missing), MacDermaid et al. [159] used Eq. (5)

(5) $$k_{p}=exp\left(-\frac{G^{⋆}}{k_{B}T}\right)\frac{D}{\lambda_{0}}$$

to estimate permeability through the SC from molecular simulation of a pre-assembled bilayer of CER EOS:CER NS C24:CHOL:FFA C22 with a molar ratio of 0.5:0.5:1:1, which after equilibration for 1.5 μs at 1 bar and 303 K gives a bilayer that is ~5.4 nm thick with a liquid-disordered central core (as described in Section 3.3.3). In Eq. (5), $G^{⋆}$ is the height of the single free-energy barrier value that MacDermaid et al. [159] observed in their bilayer simulations, which was calculated as “the difference between the highest peak of the PMF and the most stable local minimum in the ordered lipid region (1.25 nm < z<2.7nm)” (G. Fiorin, personal communication, email 15 July 2021), D is the average diffusion coefficient in a plane normal to the bilayer (which they determined was nearly constant across z), and $\lambda_{0}$ is the average diffusional path length through the lipid matrix of the SC. They assumed further that the MW dependence of the D values derived in the atomistic simulations for five molecules (methanol, nonanol, ethylbenzene, phenol and thymol), logD∝(−0.0022×MW), was similar to the Potts-Guy equation, logD∝(−0.0061×MW), which when included in Eq. (5)gives (G. Fiorin, personal communication, email 15 July 2021)

(6) $$logk_{p}=log\left[exp\left(-\frac{G^{⋆}}{k_{B}T}\right)\right]-0.0061\times MW+log\left(\frac{D_{0}}{\lambda_{0}}\right)$$

The logarithms in Eq. (6) are in base 10 (note that MacDermaid et al. [159] incorrectly state that D in the Potts-Guy equation is ~ exp. (−0.0061×MW)) and $D_{0}$ is the diffusivity for a molecule of zero volume (MW=0). They then derived $D_{0}\;∕\lambda_{0}∼8.9$ cm/h by regressing the experimental values of $\log\;k_{p}$ for nine small (MW <300) molecules (the five in the D simulations plus propanol, testosterone, mannitol, and benzene) to Eq. (6) using $G^{⋆}$ for each molecule (G. Fiorin, personal communication, email 15 July 2021). Like the Potts-Guy equation, Eq. (6) reasonably matches the experimental $k_{p}$ values for eight molecules, but significantly underestimates $k_{p}$ for mannitol, which is much more hydrophilic (logP=−3.1) than the other molecules (logP between −0.7 and 3.7) causing it to permeate the SC by a different pathway [198]. In the end, because MacDermaid et al. assumed the effect of MW is the same as in the Potts-Guy equation, their permeability analysis shows only that the solubility estimated from $G^{⋆}$ is consistent with that estimated using logP in the Potts-Guy equation.

Several groups have examined the influence of additives on the permeability of small molecules through model SC systems [107,122,140,157,199]. Simulations of CPEs sometimes involve adding a large concentration of the CPE into the solvent phase and observing the transport of these molecules into the bilayer, usually without advanced sampling techniques. Other studies insert some number of CPE molecules into the lipid bilayer. Many studies infer rather than calculate the effect of the CPE on permeability or diffusivity by examining changes in the bilayer structure in the presence of the CPE. A complication of all these CPE studies is that the chosen or simulated CPE concentration in the lipids might not be representative of the experimental situation, which is not generally known and also varies with depth in the SC. As a result, simulated CPE effects are likely to be at best only qualitatively meaningful.

The first CPE study was by Notman et al. in 2007, in which a pure CER NS C24 bilayer was solvated with varying concentrations of DMSO in water [122]. They observed that DMSO at mole fractions (in the solvent) below 0.4 displaces water at the membrane interface until, at a mole fraction of 0.4, the membrane interface is saturated with DMSO. At higher concentrations, DMSO penetrates into the interior of the bilayer, which causes a phase transition from gel to liquid-crystalline. Although neither permeability nor diffusion coefficients were presented, these structural observations correlated well with experimental observations that show DMSO enhances permeability by fluidizing SC lipids [122].

Akinshina et al. explored the addition of six oil-based CPEs (monoglycerides and fatty acids of the same C18 length but a varying degree of saturation), which are naturally present in the sebum layer on the surface of the SC, to CER NS C24 bilayers [140]. Lipid CPEs spontaneously inserted into the bilayer when initially placed in the water layer above the well equilibrated bilayer. In addition, they found that when some of the CER NS molecules were replaced by a CPE (by removing two random CER molecules and placing two CPE molecules in their places), the unsaturated lipid CPEs containing at least one *cis*-double bond induced bilayer instability, whereas bilayers containing the either saturated FFAs or those with only one *trans*-double bond, remained stable. Similarly, Hoopes et al. found that adding small amounts of oleic acid to an equimolar CER NS C24:CHOL:FFA C24 bilayer decreased the density within the bilayer and increased the lateral mobility of the lipids, indicating the presence of a more fluid-like phase [162].

Wang et al. examined the mechanisms by which menthol acts as a CPE through bilayers of CER NS C24, CHOL, and FFA C24 in a 2:2:1 ratio [157]. Menthol was found to disrupt the chain packing and vertical alignment of CER headgroups, thereby decreasing water’s free energy of penetration into the bilayer. In a subsequent study [199]. the calculated PMF for quercetin in a bilayer of pure CER NS C24 showed a decreased energy barrier when menthol was present.

Lundborg et al. [107] computed permeability coefficients of benzene, codeine, DMSO, ethanol, naproxen, nicotine, testosterone and water through a mixed lipid membrane and examined the effect of four CPEs (Azone (laurocapram), oleic acid, stearic acid and water) on codeine, ethanol, nicotine, testosterone and water. The membrane was constructed as a double bilayer stack with an arrangement based on the splayed-bilayer model proposed by Iwai et al. [31] that placed the FFAs with the CER fatty acid tails, and 75% of the CHOL with the CER sphingoid tails[32]; see Fig 4c. The system contained an equimolar ratio of CHOL, FFA, and a CER mixture of CER EOS, CER NP, and CER NS C24 with a molar ratio of 13:69:18 [32,107]. In addition, the CER NP acyl chain and FFA both had a distribution of tail lengths with C20, C22, C24, C26, C28, and C30 in a molar ratio of 5:9:34:27:9:16 for CER NP and 6:13:26:39:13:3 for the FFA. The chosen “optimized” system included 0.3 water molecules per lipid headgroup. This composition was confirmed by the authors (L. Norlen, personal communication, email 14 April 2021). The periodic four-leaflet multilayer stack, which was similar to Fig. 5f but with a tiny amount of water (0.3 water molecules per lipid) in the headgroup region and all CERs NS and NP in the extended conformation, had a thickness of 10.6 nm.

For the eight permeants studied, the simulated $k_{p}$ values were, except for ethanol, smaller than the experimental diffusion cell measurements of intact human SC by factors of 3 to 3000. The relative ordering of the $k_{p}$ values generally agree with the experimental measurements with the notable exception of testosterone; it is calculated to have one of the lowest $k_{p}$ values (only codeine is lower), while experimentally it has one of the higher values (only nicotine and benzene are larger) [107]. Not surprisingly, the magnitude of permeability enhancement varied with the permeant and concentration of CPE. Lundborg et al. [107] also calculated $k_{p}$ values for the same eight permeants in the Iwai et al. [31] model system with CER EOS removed (which is the basis of the SPP model shown in Fig. 4f). These $k_{p}$ values were larger than those calculated for the mixture with CER EOS by factors of 2 to 80,000, and, except for water, they were also larger than the experimental measurements by factors of 1.5 to 1300. In this system without CER EOS, the relative ordering of the $k_{p}$ values did not match the experiments. For example, simulated $k_{p}$ values were lowest for water, and codeine was ranked between ethanol and naproxen, whereas, in experiments, codeine had the lowest $k_{p}$ value, and water, which was 60-fold larger than codeine, was ranked between ethanol and naproxen. Such large variations between experiment and simulation have been seen in studies of other membrane systems. While quantitative agreement with experiment is not expected, such inconsistent qualitative agreement makes drawing meaningful conclusions difficult.

In general, the simulation studies of permeability through CER membranes suggest a number of relationships that have also been observed experimentally. Denser-packed membranes decreased solute permeability, notably by raising the free energy barriers within the hydrophobic bilayer interior. To this end, hydrophilic compounds generally exhibit lower permeability compared to hydrophobic compounds of similar size. Smaller solutes are also more permeable than larger solutes with similar lipophilic characteristics. Poorly packed membrane regions offer paths of lower resistance for solutes to travel through the membrane. These principles also apply when examining CPEs; those that partition into the membrane hydrophobic region, disrupt packing, and fluidize the membrane interior help increase solute permeability. These effects can be considered when developing topically applied products to increase drug delivery by reducing the barrier function of the SC.

### 3.4. Challenges with atomistic simulations of stratum corneum lipids

While molecular simulations of systems of small molecules and their mixtures are routine, simulations of SC lipids are very challenging. First, the SC lipid matrix is a complex mixture containing hundreds of individual lipid components, so a truly representative system would necessarily require on the order of 10^5^–10^6^ atoms. Although modern computer power allows simulations of millions of atoms, atomistic simulations are computationally expensive, making studying a series of systems (e.g., to study the effect of FFA or CER tail length or composition on the structure) impractical. To avoid this issue, simulations of SC lipid mixtures generally contain a limited number of lipid species, ranging from a single CER subclass to mixtures of 1–3 CERs plus CHOL and one or a few FFAs, greatly reducing the required number of molecules (and hence computational power) needed for a representative system.

Another challenge in simulating SC lipids is related to the lamellar organization and dense lipid packing of the lipid matrix. The molecular-level details of the lamellar organization are currently unclear in experimental systems, so computationally initializing these systems in realistic configurations is difficult, because the morphology and arrangement for mixed lipid systems are unknown. Normally this would not be an issue. In a typical fluid simulation, for example, the system “forgets” its initial configuration within a short timeframe. However, the SC lipids exist in dense, gel-like phases, which greatly limits lipid mobility making the possibility that the system will transition from the initial configuration during a feasible simulation run-time unlikely. Furthermore, such systems can easily become trapped in metastable states over typical simulation times. As a result, simulated properties can be biased by the initial, assumed configuration. Given the typical timescales studied by MD, it is likely this bias remains even in the several studies that have employed simulated annealing, in which simulations are performed at temperatures higher than those of skin in order to accelerate lipid mobility and thus equilibration. A further complicating issue is that lipids in the SC are composed of stacked lamellae and any realistic representation of the SC lipid matrix therefore requires the simulation of large multilayer systems, further increasing system size and computational cost.

An alternative approach for addressing equilibration issues, described by Moore et al. [115], applies a simulated-tempering like equilibration methodology, in which the system takes a random walk through temperature space, thereby providing energy to the system for local rearrangements without inducing a phase change. This random walk molecular dynamics (RWMD) method was inspired by the simulated tempering method used in MC simulations. RWMD operates by increasing the temperature, which allows the system to cross free energy barriers between local minima, and then quenching the system to the new local minimum. By rapidly repeating this process many times, a wider phase space of configurations can be explored in a short simulation time. This approach improves upon simple simulated annealing, used in several studies, because the system is not driven out of equilibrium by remaining at an elevated temperature for a prolonged time. The validity of the approach was demonstrated for equimolar CER NS C16:CHOL bilayers initialized from various highly biased, phase-separated configurations. Specifically, the CHOL-CHOL coordination numbers were shown to reproducibly converge to the same value from several independent trials using RWMD equilibration. In addition, the rate of convergence was found to be much faster than simulating at 305 K combined with simulated annealing at 340 K.

Despite the challenges of ensuring the accuracy and reproducibility of atomistic simulations from pre-assembled configurations, nearly all atomistic simulation studies of the SC lipids have been conducted from pre-assembled bilayers with only a few performed on multilayer systems [147,177,200,265]. Simulations of multilayers require on the order of 100,000 atoms. While this can be accessible to atomistic models, performing multiple trials or reaching sufficient timescales to ensure equilibration makes the study of such systems computationally prohibitive. This also raises concerns as to whether or not the results reported are biased by the initial configuration, and in the case of bilayer simulations, lipid interactions with the water on the top and bottom of a bilayer. Simulating CERs in the extended conformation requires multilayer systems, because interfacial CERs in hydrated bilayer simulations are limited to the hairpin configuration. The timescales of observing the transition from a hairpin to an extended conformation however cannot be easily accessed by atomistic simulations, requiring hundreds of nanoseconds to observe even a single transition [135]. Thus in an atomistic simulation, the CERs will typically remain in the conformation in which they were initialized, whether they are hairpin or extended [134,177].

Self-assembled structures, which remove the influence of the initial configuration on system properties, can address equilibration issues. However, the system sizes and timescales required for self-assembly of realistic systems that mimic the SC are computationally prohibitive for atomistic models. Coarse-grained (CG) models are therefore an attractive alternative to atomistic models; their lower resolution makes them computationally cheaper than atomistic models, which enables longer and/or larger simulations than are possible via atomistic simulation. CG simulations have been used to study SC lipid self-assembly as discussed in detail below.

## 4. Coarse-grained simulations of stratum corneum lipids

In complex lipid mixtures like those seen in the SC, processes like self-assembly, lipid reorganization, phase changes, and phase separation occur on much longer timescales or in system sizes that are not generally accessible to atomistic simulation. Many of these can be studied using CG models that, when properly developed, can capture the important interactions between the molecules without including the atomistic detail. The level of coarse-graining (i.e., how many atoms are grouped or “mapped” into each CG bead), determines the level of specificity of the CG model and the potential computational speedup. Once the mapping of the atomistic system to the CG beads has been established, how the CG beads interact (i.e., the force field) needs to be determined. Typically, CG force fields (models) are derived by determining CG model parameters that reproduce appropriate properties from an atomistic simulation and/or experimental work. Like atomistic models, CG models are better at predicting properties that are closely related to the properties on which they were optimized and less reliable for properties that were not included in their optimization. It is therefore important to consider the derivation of the CG model, including how it was parameterized and validated, when assessing the likely reliability of its predictions. The best CG model for one application may be unsuitable for another application [201].

Generally, as in atomistic force fields, CG force fields contain separate bonded and non-bonded components although some terms in atomistic force fields, such as proper and improper dihedral terms, may be omitted to improve efficiency. For neutrally charged and non-zwitterionic molecules, the electrostatic component may be excluded for efficiency because long-range electrostatic calculations can be computationally expensive. Because interactions in the CG model are softer and fewer than in atomistically detailed models, the computational time decreases and the integration timestep increases, allowing much longer simulation times and/or system sizes to be studied.

Force fields for CG models are typically developed using either a top-down or a bottom-up approach. In the top-down approach, the parameter set for the force field are derived to match properties acquired from the CG simulation to system properties (e.g., thermodynamic properties such as partitioning, density, and interfacial tension) from experiments or from atomistic simulations when experiments are not available. In the bottom-up approach, the force field is optimized based upon finer grained (higher resolution) simulation data, such as that acquired from atomistic simulations. Bottom-up approaches are generally structure-based, in that force field parameters are optimized to match target structural properties, such as radial distribution functions (RDFs) or other structure factors. Several bottom-up CG force field optimization techniques have been developed for a wide variety of applications, including iterative Boltzmann inversion (IBI) [202], multi-state IBI (MS-IBI) [203], reverse-Monte Carlo [204], force matching [205], and relative entropy optimization methods [206]. Interested readers can find an excellent discussion of CG methods in the review of Noid et al. [207].

A limitation of many CG simulations is that they are often only qualitatively correct because generic CG models were used (i.e., the force field was parameterized to represent a molecule class and not a specific molecule), rather than system-specific CG models derived from the corresponding atomistic simulations or experimental data for a particular molecule or groups of molecules. Thus, the goal when developing CG models for SC lipids should be to simplify the atomistic representation as much as possible, while retaining enough detail to obtain accurate results for the property/phenomena of interest. All of the CG models used in modeling SC lipids that are included in this review retain sufficient chemical specificity to be useful for predictions of some properties.

### 4.1. Coarse-grained models of stratum corneum lipids

To date, CG models (force fields) for SC lipids have been studied much less than those for proteins and phospholipids. Consequently, a significant amount of the work published thus far has been devoted toward developing the CG models. In this section we describe the development of these CG models. Table 7 lists the various CG models that have been used to simulate SC lipids and their lamellae.

In all the CG methods reviewed here, intramolecular bond stretching and bond bending between any three consecutively bonded CG beads are described by harmonic potentials. Proper and improper dihedrals, imposing secondary structure and preventing out-of-plane distortions, are often neglected with small effect. Non-bonded interactions between two CG beads are defined by parameters between CG beads of the same type (self-interactions), between CG beads of different types (cross-interactions), and between each CG lipid bead and the CG water molecules (which includes 3 or 4 water molecules per bead depending on the force field).

To reduce the total number of CG beads that must be optimized, CG force fields are usually designed to be modular (i.e., different molecules are built by different combinations and ordering of different bead types) and transferable (i.e., non-bonded interactions for each bead type are the same in all molecules containing that bead type). We note that transferability can also refer to the ability of the CG model to remain accurate at different thermodynamic states (i.e. different temperatures or phases), we refer to this as state transferable. Once non-bonded self- and cross-interaction parameters have been defined for a given bead type, they should not need to be re-derived for that bead-type in a new molecule. Depending on the CG force field, a bead type might be defined by its atomic groupings (e.g., as in the IBI and MS-IBI methods), or by the type and strength of its interactions (as in the MARTINI force field, described below), where a bead type may be used for more than one atomic grouping. In some CG force fields (e.g., MARTINI) there are pre-set standard parameters for bead-bead interactions. In other models (e.g., force fields optimized via IBI, MS-IBI and the Shinoda-DeVane-Klein (SDK) methods), self- and cross-interaction parameters for beads in these systems must be determined. However, once determined they are modular and can be transferred.

#### 4.1.1. MARTINI based models

Most CG simulations of SC lipid systems have adopted the popular MARTINI force field, which has been used widely in simulations of membranes and other biological systems containing various lipids, proteins, and sugars [222,238]. In the MARTINI force field, an average of four heavy atoms plus associated hydrogens are mapped to each bead, except for rings (such as in CHOL), which are mapped with as many CG sites as needed (typically 2 or 3 heavy atoms per bead) to keep the ring geometry. Each non-water bead is placed into one of four categories: charged (Q), polar (P), non-polar (N), or apolar (C). In addition, each bead is assigned either a strength of interaction on a scale of 1 to 5 (for polar and apolar beads) or a hydrogen-bonding capability (d = donor, a = acceptor, da = both, 0 = none for charged or non-polar beads); see [222] and supporting information in [212]. This yields 18 possible bead types for which generic non-bonded parameters for the interactions between beads of the same type (self-interactions) or different types (cross-interactions) have been pre-set based on comparisons to experimental free energy data for hydration, vaporization, and partitioning between water and several organic phases for several small molecules representative of various functional groups. For ring molecules, these standard non-bonded bead interactions are modified to reduce the size and strength of the ring-ring interactions (designated as S; e.g., SC1 identifies a C1 bead located in a ring); interactions between ring and non-ring beads are the same as between two non-ring beads (i.e., SC1-C1 bead interactions are the same as C1-C1 bead interactions).

The 18 bead types can be combined to describe all the common lipid headgroups, which are then easily joined to alkyl tails that vary in length and degree of saturation. Generic pre-set parameters for the bonded interactions (i.e., bond lengths, angles, and force constants for each) between the MARTINI bead types are also available. Force field parameterization using MARTINI is thus much simpler than bottom-up approaches such as the IBI method and other iterative approaches discussed below. However, because the interaction parameters are not specific to a particular molecule or class of molecules, the MARTINI force field may not capture the important interactions between atoms in a given molecule. Additionally, because the non-bonded interaction parameters are optimized to free energy data, the temperature dependence of the MARTINI force field is inherently incorrect (see Section 4.3 on challenges of CG simulations) [222,238]. For this reason, simulations performed at temperatures outside the range for parametrization (~270–330 K) should be considered with caution [222]. Furthermore, while CG models optimized to thermodynamic properties may provide good estimates for partitioning at a range of state points, they typically predict structural properties with much less accuracy, thus limiting MARTINI’S ability to accurately represent the crystalline packing exhibited by SC lipids [67].

**Fig. 8.**
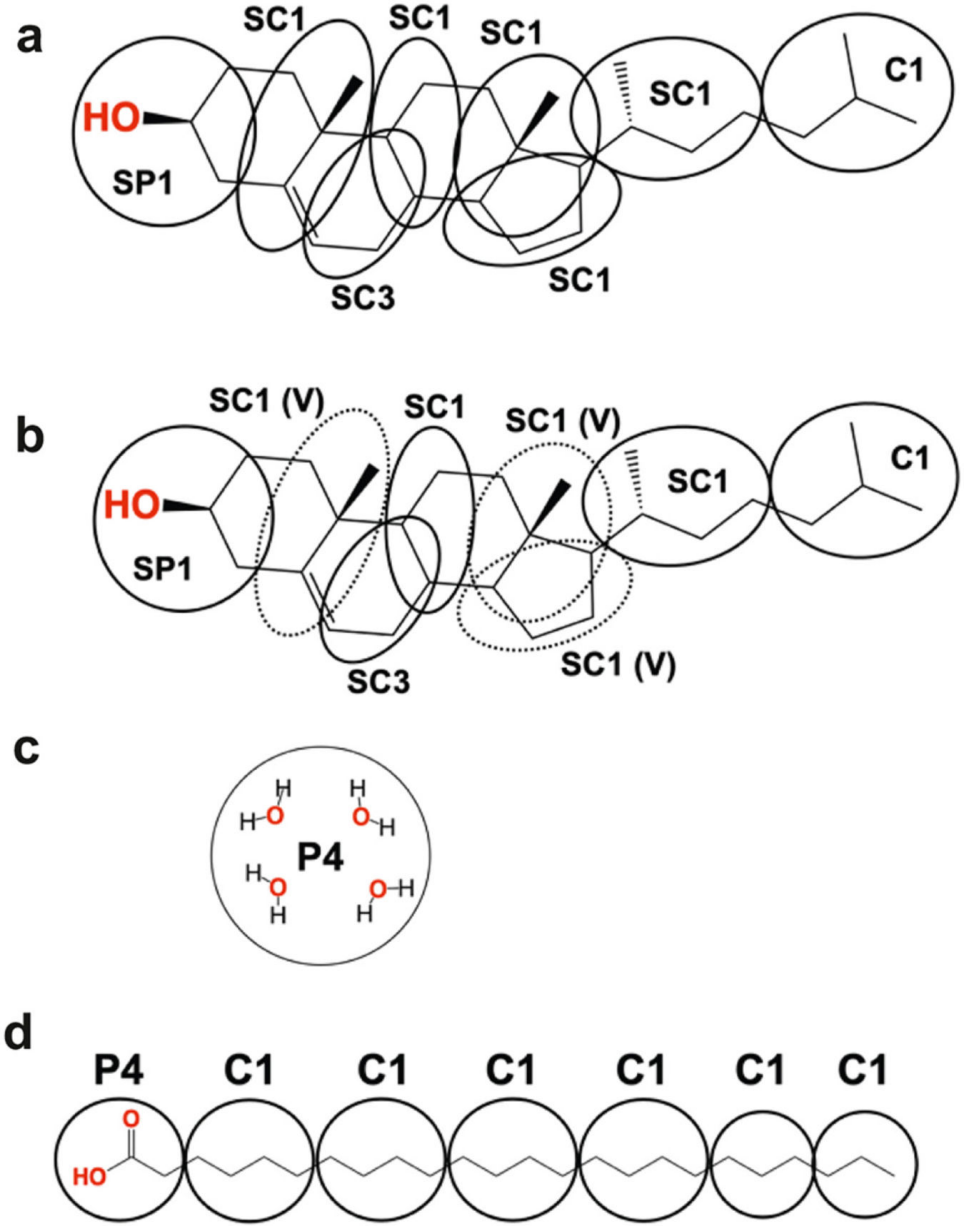
Schematic showing the MARTINI CG mapping and beads assigned for (a) CHOL in the original MARTINI force field [222], (b) CHOL in the 2015 update of the MARTINI force field where the dashed lines show the addition of virtual sites [237], (c) MARTINI water model illustrating the 4 to 1 water mapping, and (d) FFA C24 [222].

MARTINI models (force fields) for water (with four molecules mapped to one bead), CHOL and FFA molecules were developed as part of the original MARTINI force field (Fig. 8) [222,236]. Because the original MARTINI-CHOL force field can be numerically unstable and fail to reproduce experimentally observed fluidity in liquid ordered domains in CHOL-phospholipid mixtures, Melo et al. re-mapped and re-parametrized it in 2015 [237]. The new CHOL mapping (MARTINI-CHOL_new_) uses virtual interaction sites, in which forces acting on the virtual beads are propagated to accelerate and displace the non-virtual beads in the molecule to their new positions, which then sets the new positions of the virtual beads (i.e., positions of the virtual beads are determined from positions of non-virtual beads in the molecule instead of from forces acting on the virtual bead). The use of virtual sites provided additional stability and the MARTINI-CHOL_new_ model is better able to reproduce the liquid-ordered phase of CHOL-phospholipid mixtures compared to the original model [237]. However, Podewitz et al. concluded from their analysis of the DPPC:CHOL system and also the CER NS C24, CHOL, FFA C24 system that the new CHOL model may overestimate the increased fluidity [145,237].

**Fig. 9.**
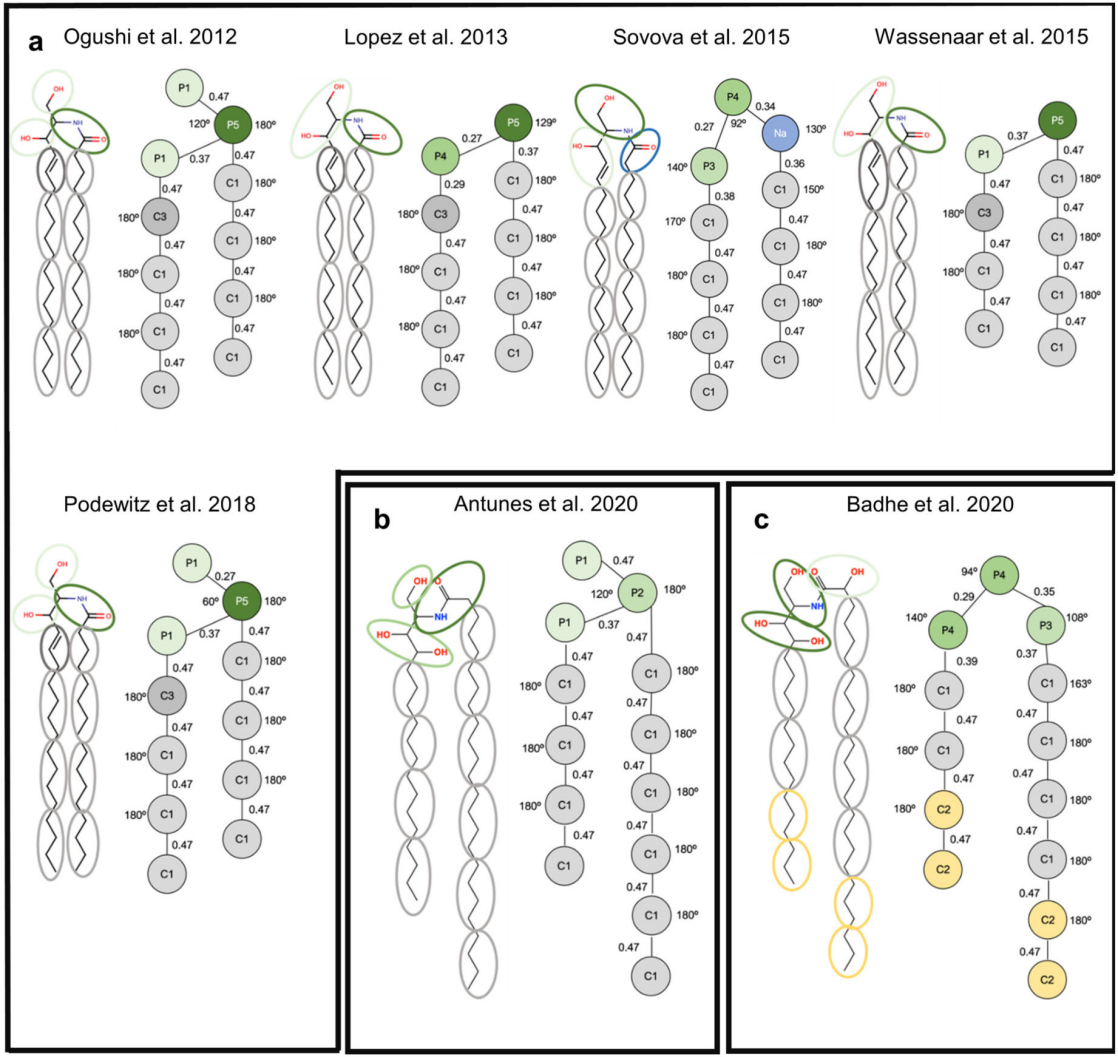
A summary of published CG MARTINI models for CERs: (a) CER NS C16 [145,155,212,221,240], (b) CER NP C24 [232], and (c) CER AP C24 [233]. The mapping scheme for each model is shown to the left of its CG representation. The CG representations denote the MARTINI bead type (inside each bead) as well as equilibrium bond lengths (between beads in nm) and bond angles (in degrees). CER NS C16 models are redrawn from Podewitz et al. [145].

Five different MARTINI models for CER NS have been proposed in the literature [145,155,212,221,240]. The number and arrangement of beads, the bead types, and bond parameters for each of these models are shown in Fig 9a for CER NS C16. Molecules with C18 or C24 hydrocarbon chains are derived from the C16 mapping by adding one or two beads containing 3 or 4 alkyl groups depending upon the version of the model. Derivation of these models combined a top-down approach for nonbonded interactions with a bottom-up approach for the bonded interactions (except for the Ogushi et al. model [212] as discussed below).

In the first MARTINI CG model for CER NS, Ogushi et al. [212] mapped the CER NS headgroup using three beads representing respectively the amide group, and the two hydroxyl methyl groups, thereby allowing rotations that affect CER motions. The unsaturated bond was combined with one CH_2_ group in the bead connecting the headgroup and fatty acid tail. The model was not optimized to describe CER NS in any way as the standard parameters for the MARTINI model were used to describe both the bonded and non-bonded interactions [222,236].

The second CER NS MARTINI model was published by López et al. as part of the development of a MARTINI model for glucosylceramide [221]. In this model, the headgroup is mapped as only two beads, in which one bead represents the amide group and the other bead the two hydroxyl groups combined. The bond between these beads was parameterized to reproduce the distance distributions found in the all atom simulations. Wassenaar et al. updated the Lopez model in 2015 using the automatic MARTINI lipid parameterization software *insane* [240]. In the updated model, the polarity of the headgroup bead containing the two hydroxyls is reduced from level P4 to P1, 5 instead of 4 carbons are mapped to the sphingoid tail beads, and bond lengths and force constants between some of the headgroup beads were changed. Validation of the proposed CER parameters were not supplied in either publication. Parameters for the Lopez et al. [221] and the Wassenaar et al. [240] models are available on the MARTINI website [241] (identified respectively as Lopez et al. 2013 with the name CER and as current with the name DPCE).

Sovova et al. [155] in their MARTINI model for CER NS C24, mapped the hydroxymethyl and amide group into one bead, grouped the second hydroxymethyl and unsaturated bond into a second bead, and combined the carboxyl group and a CH_2_ group into the third bead. To keep the parameter set as close as possible to the MARTINI force field, pre-set MARTINI parameters were used for all interactions except for the bonded parameters of the headgroup beads. These were modified to reproduce the gel phase APL and bilayer thickness as determined from united atom (GROMOS-Notman) simulations [155]. CG model parameters that did and did not include dihedral terms for the headgroup were also considered and found to produce similar results. However, the simulation time step could be almost 4 times larger if the dihedral terms were excluded, thus the final model did not include the dihedral terms.

The newest CER NS MARTINI model, from Podewitz et al. (MARTINI_CERNS-Podewitz) uses the same mapping as Ogushi et al., but re-parametrized the headgroup bond lengths, angles and force constants to reproduce angular and bond distributions from all-atom simulations of CER NS bilayers using the CHARMM-Anishkin force field [127,145]. These were then modified to reproduce the experimental APL, bilayer thickness, and phase transition temperature (which the Ogushi et al. model underestimated by 20 K) [145,146]. Parameters for MARTINI_CERNS-Podewitz are available on the MARTINI web pages [241], identified as "by Klaus Liedl" with the name CERA.

Recently MARTINI models for CER AP and CER NP have been published. In the CER AP model from Badhe et al. [233] (Fig. 9c), the headgroup is represented by three beads that parallel those in the Sovova et al. model [155]; the hydroxymethyl and amide groups form one bead, the second bead contains the adjacent hydroxymethyl groups, and the last bead combines the carboxyl and hydroxymethyl groups. The mapping and bead type assignments of the fatty acid and sphingoid tails in the CER AP model are however slightly different from the CER NS models. For CER AP C24 the last two beads of both tails are designated as type C2 instead of Cl; for CER AP C18, the two C2 beads are dropped leaving four C1 type beads. As in the Sovova et al. model [155], standard MARTINI model parameters were used for all interactions except for the bonded parameters of the headgroup beads. These were again modified to reproduce key structural properties of CER AP C24 as determined from united atom (GROMOS-Badhe [126]) simulations. Badhe et al. claim that the model should only be used at temperatures between 270 and 330 K, because this is the valid temperature range for the MARTINI parameterization [222].

The mapping for the CER NP C24 model from Antunes et al. [232] (Fig. 9b) is similar to the CER NS models from Ogushi et al. [212] and Podewitz et al. [145], except that the headgroup bead containing the amide group is designated as type P2 instead of P5. Like Ogushi et al. [212], Antunes et al. [232] used standard MARTINI parameters for the bonded and non-bonded interactions and provided no comparison with experimental data to validate the choices made.

#### 4.1.2. Iterative Boltzmann inversion (IBI) parameterized models

**Fig. 10.**
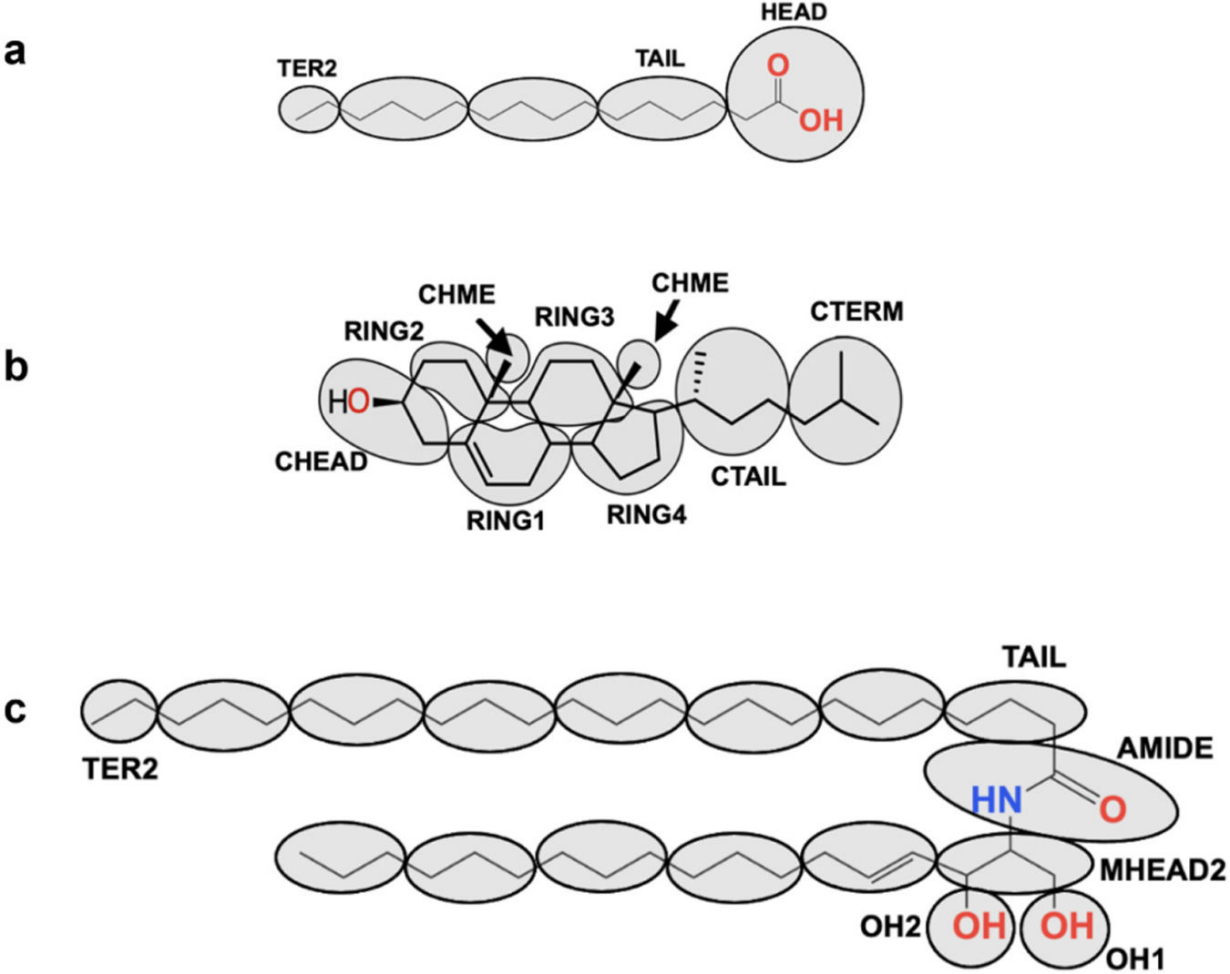
Coarse-grained mapping for (a) FFA C16 in the IBI model [210], (b) CHOL in both IBI [209] and MS-IBI [110], and (c) CER NS C24 in the MS-IBI model [151]. The MS-IBI mapping for FFAs (not shown) is the same as in the IBI model except that tail beads are mapped 3:1, as in the fatty acid and sphingoid tails of the CER NS, rather than 4:1 [151].

Hadley and M^c^Cabe were the first to apply CG modeling to study SC lipids. They developed force fields for CHOL [209], FFA [210], and water [242] using the structure-based iterative Boltzmann inversion (IBI) method [202] in a bottom-up approach that matched CG simulations with atomistic simulations of fluid and crystalline CHOL, FFA C16, and mixtures of CHOL and FFA C16. The CG mapping for CHOL and FFA are presented in Fig. 10. The FFA mapping is similar to that used in other CG models in this review. The rigid multi-ring CHOL is mapped with a hydrophilic head bead, four hydrophobic ring beads, two tail beads, and, unique from other models, two beads that represent explicitly the two chiral methyl groups, which distinguishes the “rough” and smooth faces of the molecule [209]. Hadley and M^c^Cabe showed that these features were needed for the CG simulations to exhibit the same structural behavior as those observed in experiment and atomistic simulations [209]. In addition, simulations by Rog et al. demonstrated that chiral methyl groups of CHOL have a significant impact on bilayer properties, where sterol molecules with smoother faces are not as effective as CHOL in inducing lipid ordering order when added to DPPC bilayers [243,244].

The IBI method iteratively adjusts the force field parameters describing the interactions between the CG beads until the RDF from a CG simulation matches the target RDF, where the target RDF is determined from an atomistic simulation of the identical system mapped to the CG level [202]. The IBI compatible CG water model developed by M^c^Cabe and Hadley [242] adopted the computationally efficient and novel approach of dynamically mapping four water molecules to each CG bead using the *k*-means clustering algorithm [245,270]. Bonded and non-bonded interaction parameters were derived first for pure FFA, CHOL and water, after which the non-bonded cross-interaction potentials were determined and the self-assembly of hydrated CHOL-–FFA C16 mixtures of varying composition in water studied. Stable bilayers with structures and behaviors that were in good agreement with experimental observations, including CHOL orientation, phase behavior with changing CHOL concentration, and CHOL’s fluidizing effect [246-248] were obtained. They also noted that stable bilayers did not form when FFA C16 was replaced by FFA C12 or FFA C24 in equimolar mixtures with CHOL, which was attributed to the mismatch in the length of the hydrophobic tails of the CHOLs and these FFAs.

While these IBI-CG force fields are able to self-assemble and successfully reproduce solid phases and many of the behaviors seen in their atomistic/experimental counterparts, they do not possess the desired state transferability because the force field parameters were optimized to a single state [208]. As a result, to accurately resolve both the amorphous and solid behavior of pure FFA as well as mixtures of FFA and CHOL required different CG force fields [210] to capture bilayer formation [208]. Although tailor-made IBI-CG force fields for each specific state point could be developed, they could not be used to study the effect of composition on self-assembly, which would generally involve more than one state. Despite these transferability issues, this work demonstrated for the first time that CG models can be used to study solid phases and the self-assembly of SC lipids, and that the proposed CG mappings for water, CHOL and FFA were robust.

#### 4.1.3. Multi-state iterative Boltzmann inversion (MS-IBI) parameterized models

Using the MS-IBI approach, M^c^Cabe and colleagues have developed CG force fields for the three classes of SC lipids (i.e., CER, CHOL and FFA) and water that address the non-state-transferable nature of CG models, particularly those optimized using IBI [151,229,110,231]. The MS-IBI approach extends the IBI method to derive structurally accurate, state transferable, nonbonded pair potentials [203]. State transferability is especially important for self-assembly simulations, which inherently span multiple states. One of the major limitations of bottom-up CG methods, such as IBI, is that the parameters are fitted to a single target atomistic state, which often introduces undesired artifacts in potentials between highly coordinated atoms in the gel phase. In contrast to IBI, MS-IBI accounts for structural changes over multiple states (such as highly coordinated gel phase bilayers and uncoordinated melted phase micelles) during optimization [203]. In MS-IBI the CG force field is derived iteratively to match the target RDF’s mapped from the all-atom simulations, after which the individual pair interactions are refined via simulated wetting experiments to reproduce the correct hydrophobic-hydrophilic balance [151,203]. Inaccurate representation of the hydrophobic-hydrophilic balance affects the entire system by unphysically altering the lipid packing in hydrated lamellar states [151].

The MS-IBI method was developed by Moore et al. [203] and subsequently used to develop force fields for pure CER NS C24 and C16 and FFA C24 [151], their interactions with water, and lipid–lipid cross-interactions [231] between CER NS C24 and FFA C24. A MS-IBI force field for CHOL and cross-interactions between it and CER NS C24 and FFA C16 and C24 have been developed recently [110].

The CG mappings for CHOL and the FFAs are the same as in the Hadley and M^c^Cabe model [209,210] (Fig. 10) except for a 3:1 rather than 4:1 mapping for the FFA tail beads [151]. This same tail bead mapping is also used for the alkyl and sphingosine tails of CER NS (Fig. 10c). Mapping for the CER NS headgroup is similar to that used by the Lopez et al. [221] and Wassenaar et al. [240] force fields in terms of the atom groupings; however, a key difference is that the hydroxyl groups on the C1 and C3 carbons of the sphingosine chain are each mapped to a single distinct bead (identified as OH1 and OH2, respectively), which was found to be necessary to represent the in-plane packing of the headgroups correctly [151]. All beads interact through spherically symmetric potentials. The non-bonded pair interactions were optimized successively beginning with the lipid-lipid self-interactions, followed by lipid-water interactions for each lipid species, and then cross-interactions between two lipids (e.g., FFA-CER, FFA-CHOL and CHOL-CER) [110,231]. Interactions between the CHOL tail and water were further refined using simulated wetting measurements to ensure the hydrophobicity of the CHOL tail group is accurately captured [110]. The CHOL tail force field was simultaneously optimized using target data from simulations of pure CHOL and CHOL mixed with FFA or with CER NS, making the tail beads transferrable between lipids [110]. The CER NS model has also been shown to be readily applied to other CERs, including CER NP, AP and AS, without additional parameterization [249].

#### 4.1.4. Shinoda-DeVane-Klein models

A recent model for CER NS C24, CER EOS, CHOL and FFA C22 was derived using the method described in Shinoda et al. SDK model [159,239]. Similar to MARTINI, the SDK derived models utilize partition coefficients to determine non-bonded interaction potentials, which are represented as Lennard Jones potentials (as in the MARTINI force field) with only two adjustable parameters [239]. However, like the IBI method, parameters for the non-bonded potentials are determined by iteratively modifying their values and comparing CG simulation data with target atomistic simulation data. Compared with MARTINI, the SDK model is somewhat more detailed, with a 3:1 mapping, and uses softer interactions, which together allows better reproduction of thermodynamic properties such as heats of vaporization and surface tension [201,235].

**Fig. 11.**
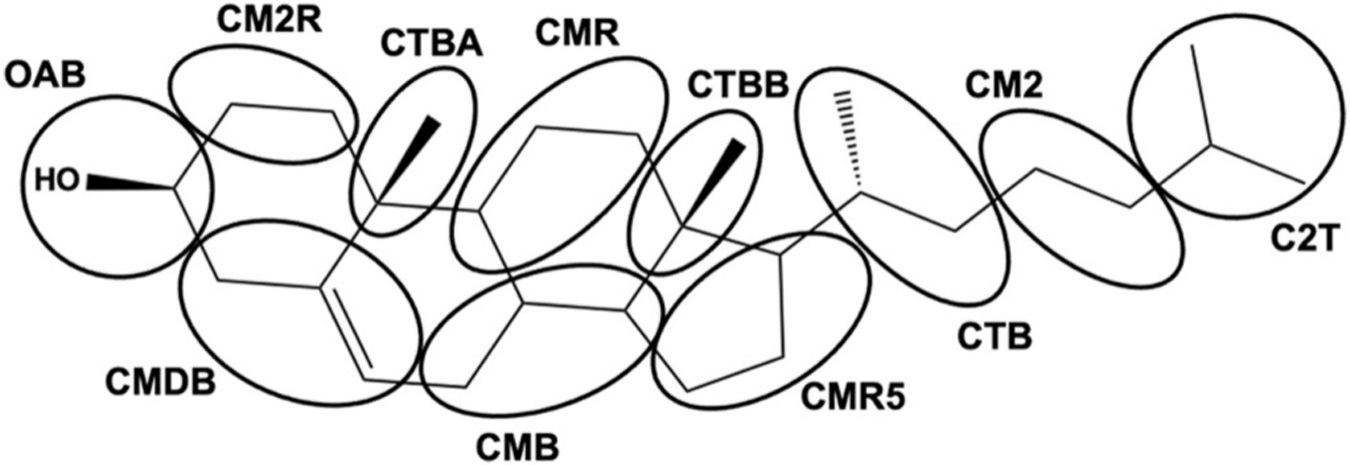
Coarse-grained mapping for CHOL used in the SDK model [234].

The SDK-CG mapping scheme chosen for CHOL consists of 11 beads (Fig. 11) [234], including two that represent explicitly the two chiral methyl groups as in the IBI and MS-IBI models [110,209]. Mapping for CER NS C24, CER EOS and FFA was not described explicitly. The authors state that the interaction parameters for the CG beads were taken from parameters developed previously for “liquid hydrocarbons, alcohols and lipids,” combined with new parameters (used in the headgroups of the CER and FFA) derived from thermodynamic data for formamide, *N*-methyl-formamide and butyric acid [159]. Unfortunately, details of how the parameters were obtained are not provided in their publication.

### 4.2. Observations from coarse-grained simulations

We now review observations in the literature from CG simulations of SC lipid systems. The CG force fields presented in Section 4.1 have been used by their developers as well as others to perform simulations of pure CER systems or mixtures of one or more CERs with other SC lipid components. Initially CG simulations were conducted on pre-assembled hydrated bilayers, which are discussed first. More recently, pre-assembled multilayer systems have been considered as well as self-assembled bilayer and multilayer systems, which are also described. Table 7 summarizes studies that have used CG simulations to examine pure CERs and SC lipid mixtures.

#### 4.2.1. Structural properties of pre-assembled lamellae

We focus first on CG simulations of pre-assembled bilayers beginning with CG simulations of pre-assembled bilayers of pure CERs with the MARTINI force field, after which we consider bilayers of SC lipid mixtures. Table 8 summarizes the results and lists the APL, bilayer thickness, VPL and tilt angle for hydrated pure CER bilayers derived from CG simulations when reported.

Ogushi et al. [212] used their CER NS C18 MARTINI force field to simulate a pure CER bilayer as well as a phospholipid bilayer containing 10% CER. They reported for the pure CER bilayer an APL of 63 Å^2^ and a bilayer thickness of 39 Å at 300 K which is in poor agreement with an APL of 46 Å^2^ and bilayer thickness of 36 Å from atomistic simulation for the same composition using the CHARMM-Wang force field [152]. The number of lipids in these simulations was small (42 and 64 lipids total for the mixed and pure lipid systems respectively), which may have affected the results [212].

Sovova et al. [155] performed simulations of pre-assembled bilayers with their CER NS C24 MARTINI force field to study phase behavior as a function of temperature and hydration. They observed an APL at 300 K of 46 Å^2^, which is larger than the ~42 Å^2^ obtained from atomistic simulation with CHARMM-based force fields [130], but matches the value from their GROMOS-Notman UA simulations. Although good agreement between the bilayer thicknesses of the CG and atomistic systems was obtained, the tilt angle of the bilayer tails observed in the atomistic simulations (22° and 24° respectively in simulations with GROMOS and CHARMM) was absent in the CG simulations. This discrepancy suggests that the CG tails do not pack as tightly as the atomistic tails [155]. In simulations at temperatures varying from 300 to 360 K, the authors identified a gel-liquid phase transition between 340 and 345 K, which is lower than the experimental value of 366 K [267]. Sovova et al. [155] also simulated several systems containing a six- or eight- leaflet hydrated multilayer stack (with periodic boundary conditions on all sides) in which the water layer separating the bilayers in each system was a constant thickness (i.e., similar to Fig. 5e except that the simulation box includes three (or four) bilayers instead of just one) and the thickness of the water layer was varied between systems from 0 to 28 water beads per lipid (each bead represents four water molecules) at 300 K (gel phase) and at 360 K (liquid crystalline). At 300 K with 0 and 28 water beads per lipid (equivalent to 0% and 76% water by mass), the bilayers retained their lamellar conformation, whereas at 360 K, regardless of the hydration, the bilayers formed micellar structures, which increased in diameter as the amount of water increased. Finally, in simulations without water, two stacked bilayers built with CERs in the hairpin conformation at 30% larger than the equilibrium APL (46 Å^2^) were equilibrated. The final APL was again 46 Å^2^ and approximately 5% of the CER tails adopted the extended conformation, demonstrating that the MARTINI_CERNS-Sovova model can represent the extended conformation.

In a subsequent study, Paloncyova et al. [152] used the MARTINI_CERNS-Sovova force field in simulations of pre-assembled bilayers of pure CER NS CX, where X was varied from 2 to 24 by including none or up to six CG tail beads. The systems were fully hydrated with at least 28 water beads (representing 112 water molecules) per lipid. They also simulated hydrated bilayers of CER NS C18:l in which the monounsaturated fatty acid tail is mapped and parametrized as an oleoyl tail in the MARTINI force field [152]. In agreement with atomistic simulations [152] (CHARMM36-Wang), the CG simulations at 310 K showed lamellar bilayers that were gel phase when the fatty acid tails contained at least 8 carbons, and liquid crystalline when the fatty acid tail contained only 2 or 4 carbons, or was C18:1. Consistent with this, the APL was ~46 Å^2^ in all simulations except for CER NS C2, which was smaller, and CER NS C18:1, which was larger. The bilayer thickness increased from 32 Å for CER NS C4 to 49 Å for CER NS C24, which is larger than the atomistic simulations by ~6 Å for 12 or more carbons and by 12 Å for C4. Bilayer tilt was not reported for the CG simulations.

Podewitz et al. compared structural and thermotropic parameters for pre-assembled bilayers of pure CER NS C16 simulated with 5 water beads/lipid (20 molecules/lipid) using their model (MARTINI_CERNS-Podewitz) and the four other MARTINI models in Fig. 9 [145,146,155,212,221,240]. Generally, the APL, VPL, and bilayer thickness (*d_HH,e_*, calculated as peak-to-peak distance in electron density profiles) for the gel state at 320 K were found to be similar for all five MARTINI-based force fields. This was also true for the liquid-disordered state at 380 K, except for the Lopez et al. model, which was unstable at *T* > 330 K, and for the bilayer thickness predicted by the Wassenaar et al. model, which was significantly underestimated (1.2 nm compared to ~2 nm for the others) [145,146].

Of the five MARTINI CER NS models considered by Podewitz et al., MARTINI_CERNS-Podewitz was the only one to exhibit an order-disorder phase transition at 365 K [145,146], in agreement with experimental transition of ~365 K [165,178]. In contrast, the estimated phase transition temperatures were lower than the experiments by ~20 K or more for the Ogushi et al. and Wassenaar et al. models, and higher by ~10 K for the Sovova et al. model [145,146]. The latter result is larger by ~40 K than the phase transition between 340 K and 345 K reported by Sovova et al. [155] from CG simulations using their own MARTINI model for CER NS C24. As reference, in experiments with CER NS with acyl chains from C16 to C24, changing the tail length had minimal effect on the transition temperature [165,178]. These results illustrate how CG models may not provide reliable predictions of properties that were not included in the model’s optimization. As discussed in more detail below (see Section 4.3), the MARTINI force field is known to yield unreliable thermotropic results [145,155], unless, as Podewitz et al. did, the standard MARTINI parameters for bond lengths, angles and force constants of the headgroup beads have been adjusted to reproduce thermotropic data, in this case, specifically the experimental phase transition temperature [145].

Overall, bilayer simulations of CER NS C16 using MARTINI_CERNS-Sovova and MARTINI_CERNS-Podewitz models [145,146] agree best with atomistic simulations using the CHARMM-Anishkin force field [127] for bilayer thickness and tail order at 320 K. However, all five models failed to accurately reproduce the atomistic APL and all significantly underestimated the lipid tail tilt angle, which ranged 0.05 to 1.16° at 320 K compared with 17° observed in the atomistic simulation [145,146]. Podewitz et al. attributed the lack of tilt to the parameters of MARTINI CG force fields; this is a reasonable hypothesis given that the bond/angle parameters are tuned but CG bead sizes retain the standard values specified by the MARTINI parameterization [236]. Alternatively, Sovova et al. claimed that the loss of atomistic detail in CG models renders them generally unable to reproduce tilt in the CER tails [155], although this statement is disproven by other published CG models where tilt is observed [110,151,231]. The small differences in the abilities of the five MARTINI force fields for CER NS to reproduce atomistic simulation data implicates the models themselves as the cause of their shortcomings. Limiting factors include the use of standard CG bead sizes, along with the CG mappings used in all of the CER NS MARTINI models presented thus far, which lack directional headgroup interactions. These interactions have been shown to influence the properties of CER systems [151]. Overall these MARTINI force fields are more representative of a generic lipid than CER NS specifically.

Podewitz et al. [145,146] also self-assembled hydrated bilayers at 300 K and 340 K for 23 different molar ratios of CER NS C24, CHOL, and FFA C24 including 13 binary mixtures and 7 ternary mixtures. Simulations were performed using the MARTINI_CERNS-Podewitz force field combined with force fields from the MARTINI developers for FFA C24 and the original (2007) and new (2015) versions for CHOL (described in Section 4.1.1). Table 9 compares results from Podewitz et al. [145,146] at the same compositions as observations from all other published CG studies of bilayers containing ternary mixtures of CER NS C24, CHOL and FFA C24 (Table 7). Seven papers from Gupta and colleagues [200,223-228] describe MARTINI simulations of hydrated bilayers of equimolar CER NS C24, CHOL and FFA C24 with assorted additives including nanoparticles, proteins, and CPEs. Unfortunately, except for order parameters, structural properties of the bilayer in the absence of any additives are not presented in these papers. MARTINI simulations of pre-assembled bilayers containing a 1:1:0.5 molar ratio of CER NS C24: CHOL:FFA C24 using the MARTINI_CERNS-Lopez force field are reported in two papers from Shi and colleagues [214,215]. The only other CG study of bilayers with this ternary mixture is from Shamaprasad et al. [110], which used MS-IBI force fields.

For the properties listed in Table 9, there is little difference in the bilayers simulated using the two MARTINI CHOL models while keeping the MARTINI models for CER NS and FFA C24 the same, except for the phase of the lipid mixture, which is identified as liquid-ordered for the new CHOL model and as gel for the original CHOL model when the molar ratio of CER NS:CHOL:FFA is 1:1:1 and 1:1:0.5. APL values calculated from the MS-IBI and MARTINI models are similar. Thickness of the inner bilayer of the 6-leaflet multilayer stack is consistently larger by 3–5 Å compared with a hydrated bilayer in MS-IBI simulations of systems with the same composition. Also, for the hydrated bilayer at the same compositions, the thicknesses from simulations performed using MARTINI_CERNS-Podewitz (calculated as *d*_*WI*,1/2_) are 1.2 to 2.7 Å larger than the MS-IBI simulations (calculated as *d_HH,m_*), and ~ 5 Å smaller than those performed using MARTINI_CERNS-Lopez (calculated as *d_HH_* for an unspecified density profile) [145]. Some of these differences are probably due to the different methods for calculating thickness; for example, hydrated bilayer thicknesses calculated as *d*_*WI*,1/2_ rather than *d_HH,m_* for the 1:0.5:1 molar ratio of CER NS:CHOL:FFA was ~4 Å larger from MS-IBI and atomistic simulations (see bilayer thickness values listed in parentheses in Table 9). For the lipid mixtures listed in Table 9, interdigitation of FFA C24 is greater than or equal to interdigitation of CER NS C24, while, as expected, interdigitation of the shorter CHOL molecule is negligible or small. For simulations with interdigitation values listed for individual lipids and all lipids combined, the total lipid result is either the average of CER and FFA interdigitation, or larger than the interdigitation values for both CER and FFA as hypothesized in Section 3.2.5. CER interdigitation values from the MS-IBI simulations are consistently larger than those from both the MARTINI and atomistic simulations by 2–4 Å.

In more recent work, Badhe et al. [233] using the MARTINI model (MARTINI_CERAP-Badhe), studied a pre-assembled hydrated triple bilayer (i.e., as in Fig. 5e but with periodic boundary conditions on the top and bottom of a simulation box containing all three bilayers) of CER AP C18:CHOL:FFA C16 in a 1:0.7:0.64 molar ratio with varying amounts of water distributed in equal amounts on the headgroups of each leaflet (0, 4.8 or 6.9 water molecules per lipid); 4.8 water molecules/lipid for each leaflet corresponds to a water layer of about 8 Å between the headgroups of two bilayers (i.e., between two leaflets)^3^. After equilibration, separated pools of water were observed between leaflets in the systems with water, and the fraction of CER molecules that changed conformation from the initial hairpin to fully extended decreased (from 23% to 12%) as the system became more hydrated; as expected, a thicker water layer forces more CERs into the hairpin conformation. Badhe et al. [233] also studied CHOL and FFA flip-flop across the triple bilayers. Flip-flop events for CHOL occurred within a leaflet and between leaflets of both the same and neighboring bilayers, whereas flip-flop events for the FFA only occurred between leaflets. For both CHOL and FFA, flip-flop events between bilayers were more frequent than within a bilayer, and the number of events increased with increased hydration and more bilayer bending around water pools.

Based on atomistic simulations by Iwai et al. [31], Antunes et al. [232] used MARTINI CG simulations to examine the proposed asymmetric molecular arrangement of an equimolar mixture of CER NP C24, CHOL and FFA C24 in which all CER molecules are fully extended with the acyl chains associating with only the FFA and sphingoid chains associating with only CHOL. The SPP model proposed by Skolova et al. [33] (Fig. 4f) was also based on results from Iwai et al. [31]. In simulations by Antunes et al. of pre-assembled dehydrated four leaflet (double bilayer) multilayer stack as illustrated in Fig. 5f) and dehydrated eight leaflet (bi-double bilayer) multilayer stack with periodic boundary conditions, some of the CHOL and FFA molecules diffused from their initial positions to the other CER chain, or showed some disorganization [232]. The authors attributed this behavior to an inability of the simulations to adequately compact the lipid structure as the system relaxed. However, their results could indicate that the assumed organization was not favored by all molecules or that the CG models may not represent critical features of the molecular interactions. Antunes et al. [232] also studied hydrated multilayer systems, in which the volume added to the top and bottom of the simulation box from the dehydrated multilayer stack system (Fig. 5f) was filled with water beads. Their observation that the dry and hydrated systems showed no significant differences is inconsistent with other work [110,147] and likely attributed to the fact that water was placed in direct contact with the hydrophobic lipid tails, which would be highly unfavorable, rather than in contact with the hydrophilic headgroup region. Steered simulations in which either a Nile red molecule or a water bead (4 water molecules mapped to a single bead) were forced to cross the pre-assembled fully dehydrated double bilayer with extended CER NS both failed when crossing the CER head-group zone for the second time, perhaps indicating again problems with either the CG force fields or the presumed conformation and positions of the individual molecules.

Using the MS-IBI parameterized models, Moore et al. [151,231] calculated structural properties of pre-assembled and self-assembled CG bilayers of pure CER NS C24 and CER NS C16 as well as equimolar mixtures of CER NS C24 and FFA C24. Excellent agreement was shown between the APL, bilayer thickness, and nematic order parameter (S_2_) of CG and atomistic systems simulated using the CHARMM-Guo force field. This agreement is expected since the CG model was optimized to reproduce the atomistic structure. The tilt angles for the pre-assembled CG systems (~ 6 to 7°) were lower than those of the atomistic systems, arising perhaps from small deviations in the lipid packing due to the loss of detail [151,231]. However, unlike the MARTINI models for CER NS [145,146,155], the tilt angles from the MS-IBI CG models were significant [151]. The ability to accurately capture the corresponding atomistic behavior was attributed to the explicit treatment of OH groups in the CG model, allowing it to capture short-ranged, directional interactions. To support this claim, a simpler three-bead headgroup model, without explicit OH interactions, that is similar to the MARTINI_CERNS-Sovova mapping, was also examined in this work [151]. For a pre-assembled pure CER NS C24 hydrated bilayer, the three-bead headgroup model produced a larger APL, larger bilayer thickness, and a similar tilt angle (Table 7) with a lower nematic order (0.91 cf. 0.98) compared to the 4-bead headgroup model, which was similar to the atomistic simulation results, except for the larger tilt angle (see Table 4). These results contradict the assertion by Sovova et al. that the loss of atomistic detail in CG models renders them generally unable to reproduce tilt in the CER tails [155]; indeed tilt has also been seen with other CG models including both MS-IBI and MARTINI models (e.g., see Shamaprasad et al. [110] and Badhe et al. [233]).

The MS-IBI parameterized CG model was also used to simulate pre-assembled systems containing mixtures of CER NS, CHOL, and FFA at 305 K [110]. The ternary mixture of CER NS C24:CHOL:FFA C24 with 1:0.5:1 molar ratio, and equimolar binary mixtures of CHOL with FFA C16 and with CER NS C24 exhibited close structural agreement with equivalent atomistic simulations. The CG model slightly overpredicts the atomistic APL for all three compositions, and slightly underpredicts the atomistic bilayer thickness for the CHOL-FFA C16 and ternary mixtures. However, unlike the MARTINI-based force fields, the CG model accurately captures the atomistic tilt angle for all compositions [110,145,146]. At 330 K, the CHOL-FFA C16 mixture exhibits a bilayer thickness (*d_HH,m_*) of 35.7 Å [110], which is slightly smaller than the experimental repeat distance of 39 Å (from X-ray diffraction at 328 K) for a mixture of the same composition, although CHOL in the experimental lamellar phase may be >50 mol% [247]. The change in APL for the CHOL-FFA C16 mixture at 305 K compared with 330 K calculated from the CG model is much smaller (30.3 to 30.9 Å^2^) than observed in the atomistic simulation (29.9 Å^2^ at 305 to 33.3 Å^2^ at 333 K) [110]. This observation is consistent with the expectation that a CG model that was not optimized to reproduce thermotropic behavior would be unlikely to accurately predict temperature dependence [110].

MacDermaid et al. [159] studied several pre-assembled bilayer systems using the SDK model at 303 K. Hydrated bilayers (as illustrated by Fig. 5a with either 5 or 20 water molecules per lipid) containing mixtures of CER NS and CHOL varying from 0 to 50 mol% (in 10% increments) showed good agreement with experimental X-ray scattering repeat distances for an equimolar mixture of CERs and FFAs [44] (i.e., the CER mixture listed in Table 1 for the SCS but without CER EOS combined with the FFA7 mixture listed in Table 2) at CHOL fractions >30% of the total CER and CHOL. However, below 30% CHOL, the model failed to reproduce the repeat distance of the main phase. Similarly, simulations of fully hydrated bilayers containing mixtures of CER EOS and CHOL varying from 0 to 50 mol% (in 10% increments) were also run. Once again, the bilayer thicknesses of the CG model agreed well with experimental data [183] for the 50 mol% CHOL system, but underpredicted the thickness for the 0% system. (See Figs. S2 and S3 in the Supplemental Information for MacDermaid et al. [159] for the CHOL mixtures with CER NS and CER EOS, respectively.) This may suggest that the model is better suited for systems in the liquid phase rather than the gel phase.

Nearly all CG studies of SC lipids have reported order parameters either to compare with atomistic simulations or to evaluate the effects of chemicals, nanoparticles and/or proteins on lipid organization and tail ordering. For example, Moore et al. [151,231] report values for nematic order parameter (*S*_2_) for CER NS, comparing simulations using the MS-IBI CG force field to atomistic systems simulated using the CHARMM36-Guo. *S*_2_ was calculated as a means of validating the structural behavior predicted by CG force fields derived in these studies. In Moore et al. [151,231], a single average value is reported for each system, where *S*_2_ is calculated from the moment of inertia of the lipid tails (as discussed in Section 3.2.6).

Other studies have reported the *S*_*cc*_ order parameter (sometimes designated as *S_Z_*) [214], typically as a function of carbon number. For example, Sovova et al. [155] report *S*_*cc*_ order for simulations of CER NS using the MARTINI_CERNS-Sovova CG force field and the UA GROMOS-Notman. Because the MARTINI_CERNS-Sovova force field does not result in an appreciable tilt angle in contrast to the UA simulations, a direct quantitative comparisons of the *S*_*cc*_ values between the two models is not really meaningful, given the significant differences in the numerical values; as discussed previously in Section 3.2.6 a complication of *S*_*cc*_ is that tilt angle and disorder in the hydrocarbon chains both affect the values of *S*_*cc*_. Comparison of the change in *S*_*cc*_ as a function of carbon number reveal that the acyl chain shows a reduction in *S*_*cc*_ at carbon 11 in the CG model as compared to 15 in the UA model. In the work of Badhe et al. [233] *S*_*cc*_ was compared for CER AP between the CG model and the GROMOS-Badhe model, finding close agreement in terms of shape of *S*_*cc*_, and much closer quantitative agreement of the numerical values than Sovova; these results suggest closer agreement of the ordering, given that the reported tilt angles did not differ substantially.

Unfortunately, the definition of the lipid tail order parameter is not provided in some studies, (e.g., [215,216,218,219]) nor is the averaging scheme employed in studies that report a single value for the tail order parameter (e.g., [145]). Furthermore, since tilt angle is often not reported or only casually discussed in many studies, the utility of these reported *S*_*cc*_ values is limited given the connection between *S*_*cc*_ and tilt angle.

#### 4.2.2. Self-assembly of stratum corneum lipid systems

A few studies have examined self-assembly of SC lipids into bilayer or multilayer structures (Table 8). Hadley and M^c^Cabe investigated the self-assembly of CHOL and FFA mixtures using their IBI-derived force field. However, Moore et al. were the first to self-assemble bilayers as well as multilayers of mixtures containing CER [231]. They were able to reproducibly self-assemble bilayers and multilayers of pure CER NS C24 and CER NS C16 [151] as well as equimolar mixtures of CER NS C24 and FFA C24 using the MS-IBI derived model [231]. The results showed good structural agreement with atomistic bilayers as assessed by APL, bilayer thickness, nematic order parameter, and tilt angle [151,231]. Compared to MARTINI models, the tilt angle for CER NS C24 bilayers from MS-IBI CG simulations show better agreement with atomistic simulations although still too small (i.e., ~0–1°, 7°, and 22° for MARTINI [145,146], MS-IBI [151], and atomistic simulations respectively [151]). Since the MS-IBI CG force field was derived to match CG and atomistic structures, a high degree of agreement is expected between the CG and atomistic structures. In addition, since both bulk fluid and lamellar states were used as targets for the optimization of the MS-IBI CG force field, self-assembly from randomized fluid-state initial configurations can be successfully achieved. In self-assembled multilayer structures, more CERs adopt extended conformations in equimolar mixtures of CER NS C24, CHOL and FFA C24 (~35%), than in equimolar mixtures of CER NS C24 and FFA C24 (~23–28%), which contain more extended CERs than in pure CER NS C24 (~15%) [110,231]. As discussed above, the arrangements proposed by Iwai et al. [31], and Skolova et al. [33] assume that all CERs are in an extended conformation, which differs from the mixture of hairpin and extended CERs in the multilayers self-assembled using the MS-IBI-derived model.

The MS-IBI-parameterized force field has also been used to simulate the self-assembly of hydrated bilayers as well as 4- and 6-leaflet hydrated multilayer stacks of CER NS C24:CHOL:FFA C24 with a 1:0.5:1 molar ratio [110]. Like bilayers in experimental samples, either isolated SC or SC lipid membranes [8], the headgroups of the inner bilayers of the 4- and 6-leaflet multilayer stacks contact another lipid layer rather than bulk water. As a result, CERs in an inner bilayer can adopt either extended or hairpin conformations, whereas CERs in a single hydrated bilayer are limited to the hairpin conformation. Comparisons of structural properties of the 2-, 4- and 6-leaflet systems show significant differences, primarily due to the presence of extended CERs in the inner leaflets of the 4- and 6-leaflet systems. For example, the more efficient packing of extended CERs caused the average APL across all leaflets in the 2-, 4- and 6-leaflet stacks to decrease (33.7, 33.40, and 33.14 Å^2^). At the same time, bilayer thickness increased (50.82, 52.94, 53.81 Å) for the 2-, 4-, and 6-leaflet systems to approach more closely the experimental repeat distance of 53–54 Å determined by small angle X-ray diffraction (SAXD) on the SCS synthetic lipid mixture (see Tables 1 and 2) without CER EOS (so that only the SPP forms) [8,110]. These findings suggest that a hydrated multilayer stack of at least 6-leaflets (with 4 inner leaflets that do not contact bulk water) may provide a better representation of experimental data than the hydrated bilayer. The effect of CHOL content on SPP-forming systems of equimolar mixtures of CER NS C24 and FFA C24 was explored in self-assembled 6-leaflet systems. The bilayer thicknesses determined from simulation decreased with increasing the CHOL/CER NS mole ratio from 0.2 to 1, which differed from the constant repeat distance observed in SAXD for increasing amounts of CHOL in the SCS CER mixture (Table 1) without CER EOS combined with an equimolar amount of FFA5 (Table 2) [44]. The difference between the self-assembled simulations and experiments may reflect the different lipid compositions of the experiments and simulations, especially the presence of FFAs with multiple tail lengths instead of only FFA C24 as in the simulations. In addition, CHOL phase separates in the experimental systems for CHOL/CER NS ratios at or above ~0.5 [44,86], which means the composition of the SPP phase in these mixtures is not the same as the overall system composition or the composition in the simulations where phase separation has not been observed.

Podewitz et al. were the first to self-assemble bilayer systems using a MARTINI-based model [145]. They self-assembled bilayers of CER NS C24, CHOL, and FFA C24 at 23 different molar ratios from bilayers containing randomly mixed components using MARTINI_CERNS-Podewitz [145], the original MARTINI force fields for water and FFA [222,236], a either the 2007 or the 2015 MARTINI model for CHOL [222,237]. Simulations were performed at two temperatures, 300 and 340 K, chosen to be below and above the experimentally observed phase transition temperature for the equimolar ternary mixture. One of three different phases (liquid disordered, liquid-ordered, and gel (hexagonal) packing) were observed depending on the temperature, composition and which CHOL model was used. Thermotropic behavior in terms of structural and dynamic properties were presented in a series of ternary phase diagrams, which show that (1) the APL remained constant for the different phases and controlled by the CER composition, (2) the bilayer thickness decreased with increased CHOL concentration in the gel phase, but was mostly independent of composition for the liquid-disordered phase, (3) lateral self-diffusion and the area compressibility modulus changed significantly with phase change, and (4) increased CHOL decreased the order parameters of CER and FFA in the gel phase, but increased the same parameters in the liquid-disordered phase. Simulations of the equimolar CER:CHOL:FFA system with the 2007 CHOL model predicted a gel phase at 300 K, in agreement with experiment, while the 2015 CHOL model predicted a liquid-ordered phase. This result, along with separate simulations for the DPPC:CHOL system led Podewitz et al. [145] to conclude that the increased fluidity of the 2015 CHOL MARTINI force field may be overestimated. However, it should be noted that SC lipids in experimental studies organize into mostly dehydrated multilayer structures, and the self-assembly of multilayers is vital to producing a reliable model of SC lipid membranes. Thus far, the MARTINI model has not been used to self-assemble multilayer systems.

In self-assembly simulations using MARTINI, Antunes et al. [232] observed that equimolar mixtures of CER NP C24, CHOL and FFA C24 randomly dispersed in a vacuum formed disorganized structures, whereas dispersing the same mixture between water layers produced a single bilayer (i.e., only two leaflets) with all CERs in the hairpin configuration. Apparently water was necessary to force the lamellar organization with a single bilayer favored energetically over the multi-leaflet arrangement proposed by Iwai et al. [31] and assumed in pre-assembled simulations by Antunes et al. [232] (as described in Section 4.2.1) in which CHOL and FFA are exclusive neighbors to the sphingoid tail and the fatty acid chain, respectively of the fully-extended CERs.

The SDK model was the first CG model used to attempt self-assembly of SC lipid lamellae containing the LPP-forming CER EOS [159]. Self-assembly was observed for pure CER NS C24 and CER EOS, and binary mixtures of either CER NS C24 or CER EOS with CHOL (all containing a 5:1 molar ratio of water to lipids) beginning from fully randomized configurations. The pure CER NS system formed a homogeneous lamellar phase, whereas the other systems had several non-uniform lamellar domains. Additionally, more complex systems including a four-component mixture of CER EOS:CER NS C24:CHOL:FFA C22 with a molar ratio of 0.5:0.5:1:1, and also an equimolar mixture of CER EOS, CER NS C24, and CHOL were examined; little information was provided about simulations of the latter system. The CG simulations of the four-component mixture started with a 20-nm-thick lipid slab consisting of a ~ 15 nm thick randomized lipid mixture centered between two hydrated pre-assembled lipid monolayers (leaflets), which together were ~ 5 nm thick; thick water layers on each outer leaflet (10 water molecules per lipid in the slab divided equally) prevented lipids from leaving the lipid slab. A *dehydrated* version of this model was designed to exclude water from entering the region within ±6 nm of the midplane of the lipid slab by adding a repulsive potential applied selectively to the water beads; the dehydrated model still included 10 waters per lipid outside the outer leaflets. Over 2 μs of simulation, molecules in the randomized central layer of both models were incorporated into the outer leaflets, which caused the central layer to shrink in thickness with a compensating increase in the surface area. The final thickness of the dehydrated model, which retained a relatively isotropic central region, was ~13 nm. In contrast, the hydrated model displayed a thin region (~6 nm) of essentially two leaflets that coexisted with a thick region (~11 nm) containing a disorganized lipid core with ~1.7 water molecules per lipid within the lipid core (i.e., excluding lipids in the outer leaflets) as small droplets in contact with lipid headgroups (i.e., as inverse micelles). The authors hypothesize that the inverse-micellar structure surrounding water droplets is a metastable transition state between the SPP and LPP. Notably, the distribution of headgroups from both the dehydrated and hydrated models differ significantly from experimental X-ray and neutron diffraction data of the LPP in synthetic lipid systems, which suggest that, unlike the self-assembled models, the lipid headgroups are predominantly located at the boundary of the unit cell (~ ±6.5 nm from the center) and also, in smaller quantity at ~ ±2 nm from the center [27,72].

#### 4.2.3. Permeability and transport properties

Seven papers from the group of Shi and Qiao published between 2015 and 2020 used the MARTINI force field to simulate CER NS C24 (MARTINI_CERNS-Lopez), CHOL (original MARTINI-CHOL), and FFA C24 (slightly modified from the original MARTINI FFA C24) [222,236] in a bilayer with a 2:2:1 molar ratio of CER:CHOL:FFA [214-217,220,250]. They used these simulations to study the influence of chemical permeation enhancers (menthol, borneol or borneolum) on SC lipid permeation by drugs exhibiting a range of octanol-water partition coefficients (P): osthole (*P* = 6300), ligustrazine (*P* = 20), 5-fluorouacil (*P* = 0.13) and others [214-217,220,250]. The authors derived new MARTINI compatible force fields for the CPEs and drugs. Simulations were performed on a pre-assembled mixed-bilayer built by the Packmol software package [268]. In general, the CPE entered the bilayer and aggregated in the tail region thereby disrupting tail packing and opening pathways for permeation. They also observed that borneol weakened the bilayer by interfering with hydrogen bonding between headgroups. Higher concentrations of the CPEs were found to decrease tail order parameters and increase the permeability, while also forming some water channels, and, at the highest concentrations, destroying the bilayer structure altogether. Higher temperatures were reported to fluidize the bilayer and increase CPE and drug penetration. In all these simulations, system sizes and timescales were on the same order of magnitude as atomistic simulations, so the use of CG models was not necessary.

Gupta and colleagues simulated a bilayer containing an equimolar mixture of CER NS C24, CHOL and FFA C24 using the MARTINI_CERNS-Sovova force field for CER NS and the original MARTINI force fields for CHOL, FFA C24 and water [222,236]. They used these simulations in a series of studies, published in six papers, that examined nanoparticle (NP) penetration into and through the pre-assembled hydrated bilayer, or, in one paper, a pre-assembled hydrated 4-leaflet multilayer as in Fig. 5c [223] (i.e., a hydrated double bilayer without water between the bilayers) as a function of NP type (gold [224,226,228], dodecanethiol-coated gold [225], a fullerene C_60_ [227]), size (1–6 nm), charge [225], a patterned hydrophilic/hydrophobic surface chemistry [223]. In addition, they investigated permeation of protein molecules (horseradish peroxidase [223,226] or interferon-alpha [228]) in the presence and absence of NP. Their most recent paper describes simulations of the effect of several chemical permeation enhancers (FFA C18:1, FFA C16, FFA Cll, geranic acid, dimethylsulfoxide, geraniol, glyceryl monooleate, isopropyl palmiate, limonene, and octylpyrrolidone) on the structure of a hydrated 4-leaflet multilayer stack (as in Fig. 5c), in which half of the water was replaced with ethanol (to mimic experimental conditions) [200]. The simulations showed that the gold nanoparticles disrupted the bilayer packing, which allowed rapid entry to the interior of the bilayer. Larger gold NPs disrupted the bilayer more but, because the free energy of their penetration is much higher, their permeability was smaller [224,225]. A nonzero surface charge on the gold NP (coated with dodecanethion) reduced permeation further [225]. The horseradish peroxidase and interferon-alpha proteins only permeated the bilayer when co-delivered with gold NPs; neither permeated the bilayer alone, remaining instead on the bilayer surface near the headgroups [226,228]. In contrast with gold NPs, pristine fullerene C_60_ molecules spontaneously form aggregates in the water layers, which absorb into the bilayer. At low fullerene concentration, the aggregates are small and disperse soon after absorption; at high concentration, the larger fullerene clusters remain aggregated in the bilayer interior. Nanoparticles simulated with different patterns of the same hydrophobic/hydrophilic surface chemistry showed different permeation. Of the surface chemistries considered, NP permeation alone or with horseradish peroxidase was most promising for NP with a 2:1 ratio of hydrophobic:hydrophilic surface beads distributed homogeneously across the surface. In the study of various CPEs, permeability of the CPE across the bilayer, as expected, depends on its size and partitioning into the lipids. Some of the larger CPEs cluster within the bilayer, which reduces their diffusion and the overall disturbance of the lipid layer packing. Experimental observations of increased electrical conductivity (i.e., ion mobility) following exposure to CPEs align roughly with the overall order parameter derived from the simulations.

In considering permeability results from CG simulation, it is important to remember that dynamic properties are likely to be inaccurate because the free energy landscape has been smoothed, which effectively removes some atomic-level friction [200,238]. Furthermore, the absence of tight tail packing for the MARTINI models may impact the diffusivity estimates through CG bilayers and the behavior of CPEs within the hydrophobic tail region of the bilayer. When parameterizing new molecules in the MARTINI force field, such as CPEs, one should validate these parameters with experimental or atomistic data to ensure the correctness and transferability of the parameters in complex mixed systems. However, perhaps due to the generic nature of the MARTINI model, this validation is generally absent for the CPE parameters derived in these studies. Also, many of the issues mentioned in Section 3.3.4 for permeability estimates in atomistic simulations apply to CG simulations. For example, (1) permeability calculated only normal to the membrane layers may not describe permeation through the SC if permeation parallel to the plane of the lipid layers also occurs; (2) permeability calculated through mixed lipid systems may not consider inhomogeneity in the lipid membrane, such as phase separated domains and grain boundaries; (3) permeability calculated for a bilayer with more than ~2 water molecules per lipid in contact with the headgroups may not describe permeation through SC or SC lipid membranes; and (4) permeability calculations cannot be directly compared to permeability coefficients measured through the skin or SC, which include corneocytes and proteins.

### 4.3. Challenges of coarse-grained simulations of stratum corneum lipids

Although there are many advantages in implementing CG models, there are limitations on the information that can be extracted from CG simulations. Many of the structural metrics previously described can be calculated from CG simulations, but some properties, such as the *S_CD_* order parameter, hydrogen bonding, and scattering patterns, require atomic-level detail and thus cannot be extracted from CG simulations because the positions of individual carbons or hydrogens are not defined. In addition, CG force fields, especially those optimized via structure-based approaches, tend to be valid over much smaller temperature ranges compared to atomistic simulations due to the use of specific states in the optimization processes. While large temperature changes may be rare in studies of SC lipid systems, changes in phase behavior (from disordered to highly ordered) driven by changes in lipid composition are more prevalent and are affected by this same limitation. Optimization techniques, such as MS-IBI, attempt to mitigate this limitation of large temperature changes by incorporating multiple thermodynamic state points during optimization. Even then, as shown in Moore et al. [151,231], there is poorer structural agreement between the atomistic and CG bulk fluid systems at 500 K compared to the bilayer states at 305 K as evidenced by differences in the atomistic and CG RDFs [203,231].

The first step in developing a CG model is the grouping (mapping) of atoms into the smallest number of beads that can realistically represent the essential chemistry and structure of the molecule. The chosen mapping scheme can significantly affect the ability of the CG model to accurately represent system behavior. This is illustrated in the study of CER NS C24 bilayers using MS-IBI from Moore et al. [151], which compared mappings that were identical except that the headgroup was mapped with either three or four beads. The four-bead mapping, shown in Fig. 10, treats the two hydroxyl groups as two separate explicit beads allowing the model to capture directional headgroup interactions from hydrogen bonding between lipids. In contrast, the three-bead mapping combines the hydroxyl groups with the backbone structure, similar to the mappings for the CER NS MARTINI models shown in Fig. 9, especially those from Ogushi et al. [212], Podewitz et al. [145], a Sovova et al. [155]. Compared with atomistic simulation, the three-site model shows a significant overprediction of the APL for the pre-assembled bilayer (46.2 compared with 39.9 Å^2^), that is similar to the other three-bead MARTINI models (Table 8) and not observed in the four-bead model; bilayer thickness is also over predicted by the three-bead model, but not by the four-bead model [151]. Because both mappings were optimized to the same targets, these differences in properties are a direct result of the mapping schemes, which is evident in the RDFs for each [151]. The observation that the MARTINI and MS-IBI methods produced similar results for the three-bead mapping suggests, at least in this case, that the mapping scheme was more important than the force field. Even then, the MS-IBI three-bead mapping had the advantage that it did exhibit, consistent with atomistic simulation, an appreciable tilt that was not captured by the MARTINI model. The four-bead mapping, with its explicit description of the hydroxyl groups, is better able to capture the in-plane order of the CER headgroups than the implicit hydroxyl group representation of the three-bead mapping.

Force fields optimized by fitting partition coefficients, such as MARTINI and SDK, can predict free energies more accurately than those derived by matching non-thermodynamic metrics [222,239]. However, the reduction in the number of degrees of freedom inherent to the CG model (caused by a reduction in the number of particles) reduces the entropic contribution to the free energy thereby causing the enthalpic contribution and temperature dependence to be incorrect [206,238]. This is discussed in detail in Marrink et al. [238]. Nonetheless, several studies have examined the behavior of model SC lipid mixtures as a function of temperature using the MARTINI force field, which is known to yield unreliable thermotropic results, except perhaps for the MARTINI_CERNS-Podewitz model, which adjusted bond lengths, angles, and force constants of the headgroup beads to reproduce the experimental phase transition temperature as well as APL and bilayer thickness [145]. Several techniques have been explored to achieve better temperature transferability in CG models by using temperature dependent potentials [251-253]; however, these methods have not yet been applied to SC lipids. Generally, simulations performed outside the temperature range used for parametrization (~270–330 K for MARTINI [222]) should be considered with caution.

An additional concern when using CG models is that the dynamics of CG systems are faster than atomistic systems because of the softer effective potentials used in CG compared to atomistic models. To further complicate the issue, the speedup in dynamic events is not constant; it depends on the system of study and thus a simple scaling between the CG and atomistic timescales is difficult to implement [238]. The inconsistency in the timescales of diffusion make it difficult to meaningfully compare permeability values derived from CG and atomistic simulations or even to compare permeability estimates among differing CG systems, although several publications have attempted to do so; e.g. [200,216,218]. Permeation molecules is in CG simulations is typically the 3–4 water molecules included in a water bead.

Because many properties of interest in membrane studies require atomistic resolution, multiscale methods have been developed for recovering atomistically detailed information from CG simulations using “back-mapping” or “reverse mapping” procedures to convert the CG configurations into atomistic configurations [254-257]. The motivation for back-mapping is to allow an atomistic model to utilize the computational efficiency of CG models; this enables simulation of long times scales (required for equilibration and for processes such as self-assembly) and large system sizes (required to model multilayer, multicomponent systems). Back-mapping relies upon the fundamental assumption that the behaviors predicted by the CG and atomistic models are sufficiently similar, such that equilibrating the back-mapped atomistic configuration requires relatively modest computational cost because it is already close to equilibrium [257]. Consequently, CG and atomistic models that predict significantly different structural properties (e.g., APL, tilt angle, bilayer height) are likely to be unsuitable for back-mapping.

Thus far, back-mapping from CG to atomistic configurations has been employed in four studies of SC lipid systems [110,158,159,233]. MacDermaid et al. [159] converted from CG to atomistic configurations by equilibrating atoms placed at random locations within the radius of each CG bead, followed by constant volume simulations for 20 ns to equilibrate. The initial placement of the atoms bears similarity to work by Rzepiela et al. [258], although, Rzepiela utilized restraints between the CG and atomistic configurations to remove high energy states. Studies from Karozis et al. [158] and Badhe et al. [233] used the method proposed by Wassenaar et al. [257], which involves the strategic placement of atomistic moieties represented by each CG bead and extensive relaxation with restraints that limit large deviations in atomic positions. Specifically, Karozis et al. [158] compared atomistic simulations initialized from a back-mapped self-assembled CG bilayer of pure CER NS C24 to pre-assembled atomistic bilayer simulations. They found differences in the density profiles, where peaks corresponding to the headgroup layer were broader in the back-mapped system. In addition, they calculated the permeability of ibuprofen through the self-assembled/back-mapped and pre-assembled bilayers and found that the free energy barrier posed by the hydrophobic tail region was much lower in the back-mapped system. These differences may be unrelated to the back-mapping algorithm. Other potential causes that should be considered include insufficient equilibration of the pre-assembled structure (in this case, simulated annealing was performed from 305 K to 360 K and back to 305 K with a total duration of only 6 ns) or differences in the equilibrium structures predicted by the CG and back-mapped atomistic models that were too large to allow for back-mapping to be useful (this work utilized the MARTINI_CERNS-Sovova CG force field and CHARMM36-Wang atomistic force fields; see Tables 4 and 8).

Shamaprasad et al. [110] introduced a simpler approach to back-mapping than the Wassenaar et al. method [257], in which the reconstructed atomistic structure is based on the location of the center-of-mass of the individual lipid molecules rather than the location of the individual CG beads. In this approach, the orientation (i.e., tail pointing in the +z or −z direction) and the conformation (i.e. hairpin or extended CER) for each lipid in the CG configuration are identified. Atomistic lipids with matching orientation and conformation are then placed at the center-of-mass of their CG counterparts. The lipid membrane with added layers of water is equilibrated using procedures typically employed to locally relax the lipids in pre-assembled systems. The resulting back-mapped configuration constructed from this approach preserves the lipid conformations and structural morphology of the CG system, with the lipid structures locally relaxed via the atomistic force field. Applying this method to a 6-leaflet self-assembled system containing CER NS C24:CHOL:FFA C24 in a 1:0.5:1 molar ratio, Shamaprasad et al. produced a back-mapped structure that closely matched the experimental repeat distance and experimentally determined localization of CHOL within the membrane, and provided close agreement of structural properties with the self-assembled CG simulation used to construct it [110]. Notably, Shamaprasad et al. utilized MSIBI-based CG force fields, which are derived specifically to match the structure of corresponding atomistic systems simulated using CHARMM36-Guo. The successful back-mapping results in Shamaprasad et al. may have occurred because this combination of force fields is well-suited for back-mapping, or because the method did not rely upon a bead-by-bead reconstruction (or a combination of both).

A significant challenge in applying back-mapping arises from the fact that, while only one configuration exists from the CG mapping (e.g., the position of each CG bead is determined by the center-of-mass of the associated atoms), a multitude of possible configurations exist for placing atoms around the center-of-mass of the CG bead (i.e., degeneracy). Poor choices of these positions can lead to unrealistic, high energy states that are far from equilibrium and may result in unstable systems. The choice of initial configurations is a key difference between the back-mapping methods from Rzepiela et al. [258] and Wassenaar et al. [257]. However, even with better choices of initial configurations, the dense gel phases typical of SC lipid systems increase the likelihood of overlapping atoms in the back-mapped configurations leading to a greater risk of high energy configurations and ultimately requiring the use of restraints or other algorithms to produce stable systems. By bypassing the bead-to-bead reconstruction of Rzepiela et al. and Wassenaar et al., Shamaprasad et al. eliminates the need for restraints, but also reduces the direct link between the CG and atomistic configurations and, as a result, increases the risk of larger computational cost to equilibrate. Clearly, further investigation of back-mapping algorithms, including how appropriate usage may depend upon the underlying CG and atomistic force fields, is necessary to be able to confidentially apply these methods for multiscale simulation of SC lipids.

## 5. Conclusions and opportunities for future directions

It is evident from the extensive discussions that the molecular simulation of SC lipid systems is an exciting and active field that can contribute to the understanding of lipid organization, structure, and barrier function. We have highlighted areas in which simulation is leading the way in providing molecular level understanding, for example with insight into the interactions between lipids, their hydrogen bonding patterns, and preferred orientations. We have also highlighted the limitations of the studies published thus far and the many challenges that lie ahead. The lipids of the SC are complex and as such, significant care is needed to ensure that any simulation study generates meaningful results. The exact models and parameters used in a simulation study and the procedures followed in running the simulations and their analysis need to be clearly documented in sufficient detail that other investigators can reproduce simulations, just as in experimental studies. There is a growing body of literature and freely available software tools to help guide researchers to implement best practices for reproducible computational studies and publications [259-263].

While experimental validation of simulation results is desirable and encouraged, comparisons between simulation and experiment must be performed with care. In many cases, the quantities obtained from simulations are not directly comparable to experimental studies. For example, simulations are often performed on hydrated bilayer systems with simplified lipid mixtures, which can provide insights in their own right, but cannot be compared directly to results of experiments on the same system in which multiple-lamella with headgroup-headgroup interfaces containing minimal amounts of water exist. Similarly, appropriate methodologies must be used for comparisons. For example, the bilayer thickness calculated from simulation depends on the method of calculation, which could lead to erroneous validation if the chosen method does not capture the same behavior as the experimental measurement. Furthermore, limitations in terms of system size and time scale over which even CG simulations can be performed may mean that the overall lamellae compositions observed in experimental systems is not the same as that being simulated. For example, bilayers composed of equimolar mixtures of CER, CHOL and FFA can be simulated even though a separate crystalline CHOL phase appears to be present in model membrane experiments and in intact SC at this composition. Similarly, experimental results can be over- or mis-interpreted and used erroneously to support computational findings. Too often authors of simulation studies are quick to declare agreement with experimental results when those results are for a completely different lipid mixture and any agreement may be mere coincidence rather than providing new insight. This is particularly true for commonly reported experimental results, such as APL, repeat distances, and density profiles, which sometimes change only a little or not at all despite variations in composition and morphology.

Certainly, when used judiciously, simulations can be employed as a partner to interpret the experimental observations from well-defined precise mixed synthetic lipid systems. While, as highlighted herein, atomistic models play an important role, they incur a high computational cost and, because SC lipids have limited mobility, can be unduly influenced by the starting lipid configuration. Computationally efficient CG models are clearly therefore an important piece of the puzzle, enabling the simulation of the long timescales required to allow lipids to equilibrate and/or self-assemble, as well as simulation of the large system sizes needed to examine multilayer systems that are more representative of experimental SC lipid membranes. However, the path forward clearly involves multiscale methods that combine the best of both approaches enabling researchers to obtain atomic level information on realistic multilayer, multicomponent lipid systems, with reduced computational cost. However, the inability to predict the experimentally observed phase separation is still a significant limitation of existing CG models of the SC thus far, which can allow lamellae to be simulated at compositions that do not occur in intact SC or in SC lipid model membranes. To address this issue, researchers may need to develop new simulation models akin to ultra-CG models used to study proteins, that provide configuration dependent resolution [264]. Furthermore, methods still need to be developed for relating transport properties (e.g., diffusion and permeability) derived from simulation to those measured in experiment. With respect to skin permeability, molecules with different properties may travel through the SC by different pathways that can change in the presence of chemical penetration enhancers or skin disease. Predicting chemical flux through skin, therefore, requires multiscale approaches that combine atomistic and CG simulations with multiphasic brick-and-mortar type diffusion models that include the microscopic heterogeneity of the corneocytes and surrounding lipid matrix. Simpler strategies might be possible for predicting chemical transport through model SC lipid membranes, although, even here, consideration of how to manage local variations in lipid morphology remains a challenge.

## Supplementary Material

Supplementary material

## Figures and Tables

**Table 1:** CER composition (mol%) of three synthetic lipid mixtures compared with SC samples from pigs and healthy humans^a^.

	SCS^b^	H- SCS^c^	SC model^d^	Pig			Human					
					
Analysis method^d^				TLC^e^			TLC^e^	LC/MS^f^				
					
SC source^g^							Surgical waste	Forearm tape strips, *n* = 2	Forearm tape strips	Forearm tape strips, *n* = 15	Forearm tape strips, *n* = 19	Forearm tape strips, *n* = 5
					
Ref	de Jager [70]	Uche [25]	Opalka [74]	Bouwstra [20]	Wertz [68]	Caussin [16]	Caussin [16]	Masukawa [87]^h^	t'Kindt [52]^i^	Janssens [56]^j^	Kawana [49]^k^	van Smeden [61]
CER												
**NS**	51	13	7.5	56.2	43.3	66.5	29.8	6.7	7.7	6.9	5.3	5.2
**NP**	16	30	22.8	17.4	10.1	8.1	29.0	21.9	22.8	26.5	24.7	26.8
**NP C16**	9											
**NH**								23.3	15.0	14.0	24.2	16.4
**NdS**		13	8.2					6.3	10.1	9.5	6.3	11.5
**AS**	4	13	23.4	3.6	12.1	6.1	18.7	3.7	9.9	4.6	4.4	2.9
**AS C16**				12.3	13.1	5.1						
**AP**	5	16	26.8	5.4	15.1	5.9	9.1	16.2	9.1	14.8	9.4	13.4
**AH**							5.1	15.8	11.1	13.1	18.3	13.3
**AdS**			1.3					0.9	1.7	1.1	0.9	1.7
**EOS**	15	15	7.8	5.2	6.3	8.2	3.5	3.0	6.7	3.8	2.2	3.7
**EOP** ^l^			1.6				2.1	0.6	1.2	1.4	1.1	1.5
**EOH**							2.8	1.7	4.4	4.1	3.2	3.2
**EOdS** ^m^			0.6						0.4	0.4	0.1	0.4
**Total**	100	100	100	100.1	100.0	99.9	100.1	100.1	100.1	100.2	100.1	100.0
**EO total**	15	15	10	5.2	6.3	8.2	8.4	5.3	12.7	9.7	6.6	8.8

a Results are reported for the 12 CER subclasses included in Fig. 2. Tables S1-S5 in the Supplementary Information lists the reported data and the calculation of mol% (if not reported as mol%) for the studies listed here as well as for several other studies including some listed in Weerheim and Ponec [38]. Wt% numbers were converted to mol% using molecular weights (Table S1) from Schmitt and Neubert [48] that accounted for differences in the CER headgroups while assuming an average of 67 carbons for all EO-type CERs and 44 carbons for non-EO CERs, except for CER AS C16, assumed to have 34 carbons.

b CER compositions for the SCS are also described in several subsequent publications from Bouwstra and colleagues. The fatty acid chain length is C24 except for CER EOS and the CER NP listed as CER NP C16; CER NP C16 is a replacement for CER AS C16, which was not available [71]. The SCS is an equimolar mixture of this CER composition with CHOL and either the FFA7 or FFA5 mixture listed in Table 2 for the SCS.

c CER compositions for a human SCS more closely match lipids found in human SC than the SCS. The fatty acid chain length is C24 except for CER EOS. The H-SCS is an equimolar mixture of this CER composition with CHOL and either the FFA7 or FFA5 mixture listed in Table 2 for the SCS.

d CER compositions for another synthetic lipid mixture chosen to match lipids found in human SC. EO-CERs are 10 mol% [88]. The fatty acid chain length is C24 except for the EO-CERs. The SC model system is an equimolar mixture of this CER composition with CHOL and the 5-component FFA mixture listed in Table 2 for the SC model plus 5 wt% cholesterol sulfate. Other versions of this mixture that replaced the mixture of EO-CERs with 20 mol% CER EOS or CER EOdS, or with 30 mol% CER EOP also produce an LPP [88].

e Thin layer chromatography (TLC) results are determined as wt%. Also, compositions depend on when the analysis was performed. Over time, more individual CERs became detectable as grouped subclasses (e.g., CER NdS and CER NS were combined in the same spot as were CER AdS and AS) were separated and identified.

f Liquid chromatography/mass spectrometry (LC/MS) results are typically reported as relative abundance, which corresponds to mol%.

g n = number of subjects included in the reported average when n was reported.

h Molar compositions listed in a previous review article [48] were calculated incorrectly; see Table S4 in the Supplementary Information for details.

i Listed mol% numbers were calculated excluding the reported values for CER OS, OP, OH and NT; these represent 3% of all the CER subclasses measured (see Table S5).

j Data from this study are also reported in papers from van Smeden et al. 2014 as mol% in Fig. 3b of reference [46] and as Wt% in Table 1 of reference [51] (although the numbers in Table 1 for AdS, and EOdS, and possibly EOP, are larger than expected, perhaps due to typographic or copying errors). See Table S5 and footnote c of Table S5 for additional information.

k Listed mol% numbers were calculated excluding the reported values for CER NSD, ASD, EOSD, OS, OP, OH, OdS, OSD and BS; these represent 2.1% of all the CER subclasses measured (see Table S5).

l CER EOP was not identified in human SC until 2003 [89].

m CER EOdS was not identified in human SC until 2011 [90].

**Table 2:** FFA composition (mol%) of three synthetic lipid mixtures compared with SC samples from pigs and healthy humans.

	SCS		SC model	Pig	Human		
				
SC source^a^	FFA7^b^	FFA5^c^			Epidermal cysts *n* = 6	forearm tape strips *n* = 22	forearm tape strips *n* = 15
							
Ref	Groen [91]	Janssens [92]	Opalka [74]	Wertz [93]^d^	Wertz [94]^e^	Norlen [95]^f^	van Smeden [46,51]^g^
No. of carbons							
16	1.8	1.8	1.3	7.1	10.7		4.3
17					1.0		
18	4.0	4.0	3.3	14.5	19.2		8.1
19					1.2		
20	7.7	7.6	6.9	4.9	6.4	5	0.8
21				3.5	2.0	0	
22	42.6	47.8	47.1	32.7	15.2	11	3.9
23	5.2			7.1	5.9	internal standard^f^	2.9
24	34.7	38.8	41.4	23.9	24.6	39	34.5
25				1.5	4.4	10	8.3
26	4.1			3.0	7.2	23	25.8
27				0.4		3	2.3
28				1.6	2.1	8	7.3
29						1	0.7
30						2	1.2
Total	100.1	100.0	100.0	100.2	99.9	102	100.2

a Reported results are the average of SC samples from n individuals; n is not listed if not reported in the paper.

b This 7-component synthetic FFA mixture is also described in several subsequent publications from Bouwstra and colleagues. A mixture with slight variations of these compositions was used in a few studies from Bouwstra and colleagues published prior to the 2008 paper from Groen et al. [91].

c This 5-component synthetic FFA mixture is also described in several subsequent publications from Bouwstra and colleagues for studies with deuterated FFA because two components of the FFA7 mixture (FFA C23 and C26) are not available deuterated. A mixture with slight variations of these compositions was used in a few studies from Bouwstra and colleagues published prior to the 2009 paper from Janssens et al. [92].

d C18 includes 5.9 mol% C18:1 and 1.9 mol% C18:2.

e C18 includes 6.7 mol% C18:1 and 1.7 mol% C18:2. The compositions listed do not include reported 0.8 wt% C14 and 0.7 25% C15.

f Compositions were reported as mol% for only the saturated species with chain lengths ≥ C20. The amount of FFA C23 was not determined separate from the internal standard.

g Compositions listed are the total of the saturated and monounsaturated species with chain lengths ≤ C30 that exceeded 0.2 mol%. The monounsaturated species was ≤0.1 mol% except for FFA C16 (0.2 mol%), C18 (2.3 mol%), and C30 (0.2 mol%); 0.3 mol% had chain lengths >C30.

**Table 3:** Atomistic and united atom force fields used to simulate model SC lipid systems.

Force Field	Year Published	Parameterization	Properties used for validation^a^	Parameters reported?
GROMOS-Scott [119]	2006	Based on the GROMOS96 43A1. New partial charges for headgroup were calculated using the Hartree-Fock method with a 6–311++g(d p) basis set.	Comparison of APL of pure CER NS C16 with experimental sphingosine APL at 368 K.	Yes
GROMOS-Notman [122]	2007	Based on GROMOS-Berger [121]. Used method in Mombelli et al. [137] to derive headgroup parameters from serine [124] and DPPC [123]	Comparison of APL of pure CER NS C24 at 323 K to experimental APL of CER NS C16 monolayers as determined from surface-pressure isotherms.	No^b^
GROMOS-Das [125]	2013	Based on GROMOS-Notman CER NS model. Partial charges for hydroxyl and ester group of CER NP and CER EOS were added.	None	Yes
GROMOS-Papadimitriou [138]	2015	Based on GROMOS-Berger [121]	Comparison of APL, tilt angle, bilayer thickness, molecular volume, lateral packing, and hydrogen bonding in headgroups for simulations of CER NS C24 at 300 K.	Yes
GROMOS-Schmitt [85]	2018	Based on GROMOS-Notman CER NS model. Added parameters for CER AP, although neither the parameters nor the methodology were reported.	None	No
GROMOS-Badhe [126]	2019	Based on GROMOS-Notman CER NS model. Added parameters for CERs NP, NdS, NH, AP, AdS, AS, AH.	None	No
CHARMM22-Anishkin [127]	2006	Based on the CHARMM22 force field. Added parameters for CER NS C16, but parameter derivation methods were not described in the publication.	None	Yes
CHARMM27-Imai [128]	2010	Based on CHARMM27. Added parameters for CER NS, although neither the parameters nor the methodology were reported.	None	No
CHARMM27-Engelbrecht [129]	2011	Based on CHARMM27 sphingomyelin model. CER AP and CER EOS headgroups were built by replacing the phosphocholine headgroup of sphingomyelin with a hydroxyl. Missing bonded and nonbonded headgroup parameters were taken from serine. Details regarding the parameterization of the ester in CER EOS were not provided.		No
CHARMM36-Guo [130]	2013	Based on CHARMM36 force field. Ab initio calculations were used to calculate bonded parameters and partial charges for the amide groups of CER NS and NP.	Comparison of experimental lamellar repeat distance of pure CER NS C16 system with simulated bilayer thickness of pure CER NS C16. Comparison of APLs between simulated pure CER NS C16 and CER NP C16 bilayers and experimental CER NS C16 and CER NP C16 monolayers as determined from surface-pressure isotherms. Comparison of thermal phase-transition temperatures between experimental and simulated CER NS C16 systems.	Yes
CHARMM36-Wang [131,132]	2014	Based on the CHARMM36 sphingomyelin force field proposed in Klauda et al. 2010 [117]. Dihedral parameters involving the amide group were optimized to match torsion scans of using fragments of CER NS and AP headgroups. The procedure for determining partial charges was not described in the publication. However, parameters are available online.		Yes
CHARMM36-Lundborg [32]	2018	Based on the CHARMM36-Wang CER AP force field. CER NP was built by removing the hydroxyl group at the acyl alpha-carbon. Quantum mechanics calculations using the restricted Hartree-Fock method and a 6-31G(d,p) basis set were used to calculate torsion scans for headgroup dihedrals of CER NP. New atomistic dihedral parameters were calculated to fit the associated torsion scan. The CER EOS model used in the publication is based on the CHARMM36-Wang CER NS force field. However, details of how the ester linkage was parameterized were not provided. Further details are provided in the SI of Lundborg et al. 2018 [32].	New dihedral parameters were validated by comparing pre-assembled atomistic configurations of crystalline CER NP C24 in a V-shaped configuration at 24, 45, 55, 75, 90, and 115 °C. A phase transition from the triclinic to monoclinic phases was observed at 90 °C (~45 °C above the experimental phase transition). The simulated crystalline CER NP C24 system melted at 115 °C, which is close to the experimental melting point of 121 °C.	Yes
Wang-Klauda CER EOS [134]	2019	Based on the CHARMM36-Wang CER NS force field. Details of the ester linkage parameterization were not provided.	None	Yes
GAFF OPLS-UA GROMOS97-54A7 [138]	2015	Fully parameterized systems are described in reference [138]	Comparison of APL, tilt angle, bilayer thickness, molecular volume, lateral packing, and hydrogen bonding in headgroups for simulations of CER NS C24 at 300 K for each force field.	Yes

a Validation involves demonstrating that simulation of pure system properties with the specific model parameters can accurately represent experimental results.

b All of the information required to calculate the force field parameters was provided.

**Table 4:** Area per lipid (APL), bilayer thickness, and tilt angle at 300–315 K as reported in published simulation studies for pure CER NS C24 bilayers. The force field used and force field type (united atom, UA or all-atom, AA) is also noted.

Author	Year	Ref.	Force Field	Force Field Type	Temp (K)	APL (Å^2^)	NLA (Å^2^)	Bilayer thickness (Å)	Thickness Calculation Method^a^	Tilt Angle (°)
Gupta	2015	[154]	GROMOS-Notman	UA	300	39.3	19.7	56.5, 55.5	*d_V_, d*_*WI*,1/*e*_	
Paloncyova	2015	[152]	CHARMM36-Wang	AA	300	45.0	22.5	41.0	*d_HH,e_*	
Papadimitriou	2015	[138]	CHARMM36-Wang	AA	300	40.4	20.2	54.3	*d_V_*	22.0
Papadimitriou	2015	[138]	GAFF	AA	300	38.0	19.0	57.2	*d_V_*	10.7
Papadimitriou	2015	[138]	GROMOS-Papadimitriou	UA	300	38.1	19.1	57.5	*d_V_*	9.8
Papadimitriou	2015	[138]	GROMOS97-54A7	UA	300	39.9	20.0	56.5	*d_V_*	10.1
Papadimitriou	2015	[138]	OPLS-UA	UA	300	37.7	18.9	55.1	*d_V_*	9.6
Sovova	2015	[155]	GROMOS-Notman	UA	300	46.0	23.0	49.0	*d_HH,e_*	22.0
Gupta	2016	[156]	GROMOS-Notman	UA	310	39.0	19.5	55.0	*d_HH,e_*	
Moore	2016	[151]	CHARMM36-Guo	AA	305	39.9	20.0	56.2	*d*_*WI*,1/*e*_	22
Wang	2017	[157]	GROMOS-Notman	UA	310	42.0	21.0			
Moore	2018	[115]	CHARMM36-Guo	AA	305	39.0	19.5	56.8	*d*_*WI*,1/*e*_	9.0
Wang	2018, 2019	[143,134]	CHARMM36-Wang	AA	305	42.8	21.4	50.5, 54.2, 45.1^c^	*d_HH,e_, d*_*WI*,1/2, *d_FWHM_*_	18.5^d^
Karozis	2020	[158]	CHARMM36-Wang	AA	300	43.4	21.7	52.7	*d_V_*	
Karozis^b^	2020	[158]	CHARMM36-Wang	AA	300	43.5	21.7	53.3	*d_V_*	
MacDermaid	2020	[159]	CHARMM36-Wang	AA	303			58	*d_V_*	

a Bilayer thickness calculation methods are denoted as follows (see Section 3.2.3): *d_V_*, the total lipid volume of the bilayer divided by the cross-sectional area of the simulation box; *d_HH,e_*, the distance between the headgroup peaks in the electron density profile; *d*_*WI*,1/*x*_, the distance between the lipid-water interfaces on either side of the bilayer defined as the location at which the mass density falls to 1/*x*, where *x* is either *e* or 2 (Wang [143] used electron density instead); and *d_FWHM_*, the distance between half of the maximum peak values in the lipid electron density profile.

b This system was initialized by reverse mapping the final configuration of a CG simulation. All other entries were simulations performed from pre-assembled configurations using bilayer building scripts such as CHARMM-GUI [133] or mBuild [160].

c A smaller bilayer thickness value for *d_FWHM_* compared with *d_HH,e_* and *d*_*WI*,1/2_ is unexpected for a pure lipid bilayer. Also, these bilayer thickness results, calculated using the electron density profile, are similar to those calculated for CER NS C24 using the mass density profile (Fig. 6) for *d_HH,m_* (48.5 Å) and *d*_*WI*,1/*e*_ (55.0 Å), but not for *d_FWHM_* (57.3 Å).

d Tilt angle is the average of the fatty acid and sphingosine chains.

**Table 5:** Area per lipid (APL), bilayer thickness, and tilt angle at 300–315 K as reported in published simulation studies for pure CER NS C16 bilayers. The force field used and force field type (united atom, UA or all-atom, AA) is also noted.

Author	Year	Ref	Force Field	Force Field Type	Temp (K)	APL (Å^2^)	NLA (Å^2^)	Bilayer Thickness (Å^2^)	Thickness Calculation Method^a^	Tilt Angle (°)
Imai	2010	[128]	CHARMM27-Imai^b^	AA	310	42.5	21.3	41.6	*d_REF_* (O in CER)	
Guo	2013	[130]	GROMOS-Notman	UA	305	39.8	19.9	43.7	*d*_*WI*,1/*e*_	17.0
Guo	2013	[130]	CHARMM36-Guo	AA	305	42.4	21.2	42.5	*d*_*WI*,1/*e*_	24.3
Paloncyova	2015	[152]	CHARMM36-Wang	AA	300	45.0	22.5	35.0	*d_HH,e_*	
Gupta	2016	[156]	GROMOS87-Berger	UA	310	38.2	19.1	47.0	*d_V_*	
Moore	2016	[151]	CHARMM36-Guo	AA	305	42.4	21.2	42.5	*d*_*WI*,1/*e*_	24.3
Wang	2017	[132]	CHARMM36-Wang	AA	310	43.6	21.8	38.5	*d_HH,e_*	16.4
Moore	2018	[115]	CHARMM36-Guo	AA	305	40.0	20.0	45.1	*d*_*WI*,1/*e*_	16.0
Wang	2018, 2019	[143,134]	CHARMM36-Wang	AA	305	43.6	21.8	39.4, 43.9, 35.4^c^	*d_HH,e_*, *d*_*WI*,1/2_, *d_FWHM_*	17.0^d^

a Bilayer thickness calculation methods are denoted as follows (see Section 3.2.3): *d*_*V*_, the total lipid volume of the bilayer divided by the cross-sectional area of the simulation box; *d*_*HH,e*_, the distance between the headgroup peaks in the electron density profile; *d*_*WI*,1/*x*_, the distance between the lipid-water interfaces on either side of the bilayer defined as the location at which the mass density falls to 1/*x*, where *x* is either *e* or 2 (Wang [143] used electron density instead); and *d*_*FWHM*_, the distance between half of the maximum peak values in the lipid electron density profile. Reference atoms used in the *d*_*REF*_ method are listed in parentheses.

b Force field parameters used to produce this data are not reported.

c A smaller bilayer thickness value for *d_FWHM_* compared with *d*_*HH,e*_ and *d*_*WI*,1/2_ is unexpected for a pure lipid bilayer; *d*_*FWHM*_ was also smaller than *d*_*HH,e*_ and *d*_*WI*,1/2_ for CER NS C24 (Table 4), which is inconsistent with the *d*_*FWHM*_, *d*_*HH,e*_ and *d*_*WI*,1/*e*_ values presented in Fig. 6 for the mass density profile.

d Tilt angle is the average of the fatty acid and sphingosine chains [143].

**Table 6:** Area per lipid (APL), bilayer thickness, and tilt angle at 300–340 K as reported in published simulation studies for equimolar CER NS C24:CHOL:FFA C24 bilayers^a^. The force field used and force field type (united atom, UA or all-atom, AA) is also noted.

Author	Year	Ref	Force Field	Force Field Type	Temp (K)	APL (Å^2^)	NLA (Å^2^)	Bilayer Thickness (Å)	Thickness Calculation Method^b^	Tilt Angle (°)
Das	2009	[161]	GROMOS-Notman	UA	340			51.7	*d*_*WI*,1/*e*_	
Hoopes	2011	[162]	GROMOS-Notman	UA	300	31.4	19.2	51.9	*d_FWHM_*	
Hoopes	2011	[162]	GROMOS-Notman	UA	340	32.1	19.7	50.3	*d_FWHM_*	
Gupta	2015	[154]	GROMOS-Notman	UA	300	30.9	18.9	51.2	*d_V_*	
Paloncyova	2015	[152]	CHARMM36-Wang	AA	300	32.0	19.6	45.0	*d*_*HH,e*_	
Del Regno^c^	2018	[147]	GROMOS-Notman	UA	305	30.4	18.6	48.0	*d_REF_* (N in CER)	10.5^d^
Del Regno^e^	2018	[147]	GROMOS-Notman	UA	305	31.2	19.1	47.0	*d_REF_* (N in CER)	11.9^d^
Moore	2018	[115]	CHARMM36-Guo	AA	305	32.0	19.6	51.8	*d*_*WI*,1/*e*_	9.0
Wang^f^	2018	[143,148]	CHARMM36-Wang	AA	305	32.8	20.1	49.1, 50.3, 44.4	*d*_*HH,e*_, *d*_*WI*,1/2_, *d_FWHM_*	
Wang	2018	[148]	CHARMM36-Wang	AA	305	32.6	20.0	49.7, 51.4, 44.7	*d*_*HH,e*_, *d*_*WI*,1/2_, *d_FWHM_*	
Yadav	2018	[163]	GROMOS-Notman	UA	310	33.0	20.2	49.8	*d_REF_* (O in CHOL, FFA C=0, & CER sphingosine 1-OH)	

a FFA C24 is fully protonated unless specified otherwise.

b Bilayer thickness calculation methods are denoted as follows (see Section 3.2.3): *d_V_*, the total lipid volume of the bilayer divided by the cross-sectional area of the simulation box; *d*_*HH,e*_, the distance between the headgroup peaks in the electron density profile; *d*_*WI*,1/*x*_, the distance between the lipid-water interfaces on either side of the bilayer defined as the location at which the mass density falls to 1/*x*, where *x* is either *e* or 2 (Wang [143] used electron density instead); and *d_FWHM_*, the distance between half of the maximum peak values in the lipid mass density profile for Hoopes [162] and the lipid electron density profile for Wang [143]. Reference atoms used in the *d_REF_* method are listed in parentheses.

c Fully hydrated bilayers with 30 water molecules per lipid.

d Tilt angle is the molar average of the tilt angles reported for each lipid component.

e Bilayers at low hydration of 2 water molecules per lipid.

f FFA C24 was fully deprotonated.

**Table 7:** Published work on coarse-grained simulations of SC lipid lamellae^a^.

Study^b^	Year	Ref	System Studied	Force Field				Initial Configuration^c^	What was studied
				CER	CHOL	FFA	H_2_O		
Hadley	2012	[208]	1:1 CHOL, FFA CX (X = 12, 16, 24); 1:Y CHOL:FFA C16 (Y=0.54, 3)		IBI_CHOL [209]	IBI_FFA [210]	IBI_H_2_O [211]	SA: other	Interactions of lipids during self-assembly, model validation between structural properties of atomistic and CG simulations
Ogushi	2012	[212]	Pure CER NS C18^d^	*MARTINI_CERNS-Ogushi			MARTINI_H_2_O^e^	PA: HBL	Validation between structural properties and diffusion coefficients of atomistic and CG simulations to examine flip-flop motions
Martins	2013	[213]	Unknown composition of CER NS C24:CHOL:FFA C24:CholSO_4_^f^; albumin microsphere, isopropyl myristate, sucrose ester	*MARTINI_CERNS-Martins (no info^g^)	no info	no info	no info	PA: 2-HBL	Interactions of protein with bilayer
Sovova	2015	[155]	Pure CER NS C24	*MARTINI_CERNS-Sovova			MARTINI_H_2_O^e^	PA: HBL, 3-HBL, 4-HBL; 4-DML (HP)	Validation between structural properties and electron density profiles of atomistic and CG simulations. CG simulations used to understand phase transitions and lipid tail conformation
Paloncyova	2015	[152]	Pure CER NS CX (X = 2, 4, 8, 12, 16, 20, 24) or CER NS C18:1	MARTINI_CERNS-Sovova^h^ [155]			MARTINI_H_2_O^e^	PA: HBL	Validation between structural properties of atomistic and CG simulations
Shi group^i^	2015–2020	[214-220]	1:1:0.5 CER NS C24:CHOL: FFA C24 (MARTINI_SC_mix_-Shi)	MARTINI_CERNS-Lopez^j^ [221]	MARTINI_CHOL_orig_^k^	MARTINI_FFAC24_mod_^l^ [222]	MARTINI_H_2_O^e^	PA: HBL	Penetration enhancers like menthol or borneol are introduced in SC bilayers to see how different molecules permeate and impact structural properties of the system
Gupta Group^m^	2016–2019	[200,223-228]	1:1:1 CER NS C24:CHOL: FFA C24 (MARTINI_SC_mix_-Gupta)	MARTINI_CERNS-Sovova [155]	MARTINI_CHOL_orig_^k^	MARTINI_FFAC24^n^	MARTINI_H_2_O^e^	PA: HBL [223-228] PA: 4-HML (HP) [200,223]	Permeation studies of how gold nanoparticles, nanoparticles with hydrophilic/hydrophobic surfaces, fullerene, proteins, or chemical penetration enhancers impact the bilayer morphology
Moore	2016	[151]	Pure CER NS C24, CER NS C16, and FFA C24	*MSIBI_CERNS		*MSIBI_FFA	MSIBI_H_2_O [229]	PA: HBL, 2-DML (HP) SA: LWmix	Validation between structural properties of pure bilayers of atomistic and CG simulations. Self-assembly of bilayer and multilayer structures to explore lipid tail conformation
Martins	2017	[230]	Unknown composition of CER NS C24:CHOL: FFA C24:CholSO_4_^f^; fullerene C_60_	MARTINI_CERNS-Martins (no info)	no info	no info	no info	PA: 2-HBL, 4-HBL	Permeation of fullerene and its interactions with bilayers
Moore	2018	[231]	1:1 CER NS C24:FFA C24	MSIBI_CERNS [151]		MSIBI_FFA [151]	MSIBI_H_2_O [229]	PA: HBL SA: WLW	Validation between structural properties of atomistic and CG preassembled and self-assembled bilayer simulations. Self-assembly of multilayer structures to explore lipid tail conformation and water molecules per lipid
Podewitz	2018	[145]	CER NS C24, CHOL, and FFA C24 at 23 different molar ratios	*MARTINI_CERNS-Podewitz	MARTINI_CHOL_orig_ MARTINI_CHOL_new_^o^	MARTINI_FFAC24^n^	MARTINI_H_2_O^e^	SA: LWmix	Dependence of bilayer structural and dynamic properties on component ratio, temperature, lipid phase, and CHOL force field
			CER NS C16	*MARTINI_CERNS-Podewitz, MARTINI_CERNS -Ogushi, -Lopez, -Wassenaar			MARTINI_H_2_O^e^	SA: LWmix	Structural properties, lateral self-diffusion, and phase transition temperatures of bilayers
Antunes	2020	[232]	1:1:1 CER NP C24:CHOL: FFA C24; unknown composition of CER NS C24: CHOL:FFA C24:CholSO_4_^f^ Nile red, polylactic acid (PLA) and poloxamer (PLX) polymers, sebum^p^	*MARTINI_CERNP-Antunes^q^ MARTINI_CERNS-Martins [230]	MARTINI_CHOL_new_^r^	MARTINI_FFAC24^n^	MARTINI_H_2_O^e^	PA: 4-DML (EX); 8-DML (EX); lipid tails point out of PA structure; water added to top/ bottom of DML in hydrated system SA:WLM or DL^s^	CER conformation; bilayer stability; self-assembly; interactions & potential of mean force of sebum and Nile red with PA system
Badhe	2020	[233]	Pure CER AP C24; 1:0.7:0.64 CER AP C18: CHOL:FFA C16	*MARTINI_CERAP-Badhe	MARTINI_CHOL_new_^r^	MARTINI_FFAC16^t^	MARTINI_H_2_O^e^	PA: 3-HBL stack; 6-DML (HP) SA: LWmix (Pure CER AP only)	Structural properties, lipid tail conformation, CHOL localization and flip-flop events
Karozis	2020	[158]	Pure CER NS C24	MARTINI_CERNS-Sovova [155]			MARTINI_H_2_O^e^	PA: HBL SA: LWmix	Back-mapped CG bilayer used in atomistic simulations
MacDermaid	2020	[159]	a) X% CHOL in CER NS C24 and in CER EOS (X = 0, 10, 20, 30, 40, 50); b) 0.5:0.5:1 CER EOS:CER NS C24:CHOL; c) 0.5:0.5:1:1 CER EOS:CER NS C24:CHOL: FFA C22 (mixture)	*SDK_CERNS *SDK_CEREOS	SDK_CHOL [234]	*SDK_FFAC22	SDK_H_2_O [235]	PA: HBL (a,c) SA: LWmix (a) SA: DL between 2 leaflets of a PA HBL (c) PA: 3-HBL (c) PA: 4-HBL between 2 LWmix (b)	Self-assembly of lipid mixtures forming SPP, permeability of small molecules in SPP, water droplet aggregation, and metastable states induced by heating or intermembrane water, back-mapped CG to atomistic droplet studies
Shamaprasad	2022	[110]	1:X:1 (X = 0, 0.2, 0.5, 1) CER NS C24:CHOL:FFA C24; 1:1 CER NS C24:CHOL; 1:1 CHOL:FFA C16; 1:0.5:0, 1:1:0, 1:1:1 CER NS: CHOL:FFA C24 where CER NS is 1:0, 0.25:0.75, 0.5:0:5, 0.75:1, or 1:1 CER NS C16: CER NS C24	MSIBI_CERNS [151,231]	*MSIBI_CHOL	MSIBI_FFA [151,231]	MSIBI_H_2_O [229]	PA: HBL SA: WLW	Structural properties of self-assembled bilayer and multilayers, lipid tail conformation, back-mapped CG to atomistic multilayer simulations

a Asterisk on the force field indicates that it was developed as part of the work described in that study.

b Studies are listed by the first author in order of year published except for papers from the research groups of Shi and of Gupta and Rai, which are listed together for each group.

c Initial configurations for pre-assembled (PA) or self-assembled (SA) simulations. Pre-assembled (PA) initial configurations are designated as HBL (hydrated bilayer, Figs. 5a and e); n-HBL (a stack of n hydrated bilayers, Fig. 5b); n-HML (n-leaflet hydrated multilayer stack, Figs. 5c and d); n-DML (n-leaflet dehydrated multilayer stack, Fig. 5f). In the HBL and n-HBL configurations, water restricts all CERs to the hairpin conformation; the initial CER hairpin (HP) or extended (EX) conformation is specified for the inner leaflets of the n-HML and for all leaflets of the n-DML configurations. Initial configurations for self-assembling systems are designated as WLW (layer of randomly configured lipids sandwiched between (i.e., phase-separated from) two water layers), LWmix (lipids and water mixed in random configuration), DL (dehydrated lipid; i.e., lipids in random configuration without water), or other (lipid-water initial configuration that was not WLW, LWmix or DL).

d Studied pure and also as 10% of a mixture with 1-palmitoyl-2-oleoyl-sn-glycero-3-phosphocholine (POPC), 1-stearoyl-2-arachidonoyl-sn-glycero-3-phosphocholine (SAPC) or 1,2-di-arachidonoyl-sn-glycero-3-phosphocholine (DAPC).

e Standard MARTINI force field for water (4 molecules per bead) [236]; not all papers specified the standard water force field, but none mentioned the inclusion of the BP_4_ anti-freeze water particle that is included with the 4 water molecules (P_4_) in the updated BP_4_-P_4_ water force field [222].

f Composition was not specified; possibly 1:1:1 CER NS C24:CHOL:FFA C24 with unknown amount of cholesterol sulfate (CholSO_4_). Sphingosine in CER NS was C16 instead of the usual C18.

g No information was provided.

h C18:1 fatty acid tail of CER NS C18:1 was modeled with the standard MARTINI mapping and parameters for the oleoyl tail of a phospholipid [222].

i Seven papers from the Shi group used the MARTINI_SC_mix_-Shi system in simulations with other chemicals including menthol ([214,216,217,219], borneol [215,217-220], osthole [215,216,218], 5-fluorouracil [217], ligustrazine [219,220], and other small molecules (baicalin, catechin, colchicine, emodin, ferulaic acid, gastrodin, imperatorin, and quercetin [220]). MARTINI force fields were developed for several of these other chemicals as part of this work. In some simulations propylene glycol replaced a portion of the water in contact with the top and bottom leaflets to match experimental conditions [216,217,219,220].

j Two beads were added to extend the fatty acid tail from Lopez et al. [221] (available on the MARTINI website with the name CER at http://cgmartini.nl/images/parameters/lipids/CER/DPCE/martini_v2.0_CER.itp) to C24.

k Original MARTINI force field for CHOL (http://cgmartini.nl/images/parameters/lipids/Sterols/CHOL/martini_v2.0_CHOL_01.itp) [222].

l Modification of the standard MARTINI force field for FFA C24 (http://cgmartini.nl/images/parameters/lipids/FA/XCA/martini_v2.0_XCA_01.itp) in which the head bead type is P3 (instead of P4), and the bond length and force constant between the head bead and the first bead of the fatty acid is 0.37 nm (instead of 0.47 nm) and 20,000 kJ mol^−1^ nm^−2^ (instead of 1250 kJ mol^−1^ nm^−2^), respectively.

m Seven papers from the Gupta group used the MARTINI_SC_mix_-Gupta system in simulations with other additives including gold nanoparticles [223-226,228], horseradish peroxidase protein [223,226], fullerene C_60_ [227], interferon-alpha protein [228], nanoparticles with hydrophilic/hydrophobic surfaces [223], and charged surfaces [225] and potential chemical permeation enhancers (dimethyl sulfoxide, oleic acid, palmitic acid, undecanoic acid, geraniol, geranic acid, glycerol monooleate, isopropyl palmitate, limonene, N-octyl pyrrolidone) [200]. In one study [200] ethanol replaced half of the water in contact with the top and bottom leaflets to match experimental conditions.

n Standard MARTINI force field for FFA C24 (http://cgmartini.nl/images/parameters/lipids/FA/XCA/martini_v2.0_XCA_01.itp) [222].

o Simulations were performed using both the original (http://cgmartini.nl/images/parameters/lipids/Sterols/CHOL/martini_v2.0_CHOL_01.itp) [222] and the new MARTINI force fields for CHOL (http://cgmartini.nl/images/parameters/lipids/Sterols/CHOL/martini_v2.0_CHOL_02.itp) [237].

p Sebum (contains squalene (SQL), palmitic acid, palmitoleic acid, tripalmitin, triolein, palmityl palmitate, oleyl oleate, cholesterol oleate, and cholesterol). MARTINI force fields were developed for Nile red, PLA, and SQL as part of this work.

q Model was not validated.

r New MARTINI force field for CHOL (http://cgmartini.nl/images/parameters/lipids/Sterols/CHOL/martini_v2.0_CHOL_02.itp) [237].

s Disordered lipids without water remained disorganized.

t Standard MARTINI force field for FFA C16 (http://cgmartini.nl/images/parameters/lipids/FA/PCA/martini_v2.0_PCA_01.itp) [222].

**Table 8:** Area per lipid (APL), bilayer thickness, volume per lipid (VPL) and tilt angle at 300–320 K for hydrated pure CER bilayers reported in published CG simulation studies.

CER	Author	Year	Ref	Force Field	Temp (K)	APL (Å^2^)	Bilayer Thickness (Å)	Thickness Calc Method^a^	VPL (nm^3^)	Tilt angle (Deg)	Bilayer Assembly^b^
NS C18	Ogushi	2011	[212]	MARTINI_CERNS-Ogushi	300^c^	63	39	Not specified			PA
NS C24	Sovova	2015	[155]	MARTINI_CERNS-Sovova	300	46	49	*d*_*HH,e*_	1.25	~0	PA
NS C16	Paloncyova^d^	2015	[152]	MARTINI_CERNS-Sovova	310	46	41	*d_REF_* (N in CER)	1.03		PA
NS C24	Paloncyova^d^	2015	[152]	MARTINI_CERNS-Sovova	310	46	49	*d_REF_* (N in CER)	1.25		PA
NS C16	Moore	2016	[151]	MSIBI_CERNS	305	42.0	44.51	*d*_*WI*,1/*e*_		6.3	PA
NS C24	Moore	2016	[151]	MSIBI_CERNS	305	39.7	52^f^	*d*_*WI*,1/*e*_		5.2–9.0	SA
				MSIBI_CERNS	305	42.0	56.6	*d*_*WI*,1/*e*_		7	PA
				MSIBI_CERNS-3bead^e^	305	46.2	61.5	*d*_*WI*,1/*e*_		9	PA
NS C16	Podewitz	2018	[145,146]	MARTINI_CERNS-Ogushi	320	46	38	*d_HH,e_*	1.11	0.12	PA
				MARTINI_CERNS-Lopez	320	48	34	*d_HH,e_*	0.94	1.16	PA
				MARTINI_CERNS-Sovova	320	46	37	*d_HH,e_*	1.01	0.30	PA
				MARTINI_CERNS-Wassenaar	320	47	34	*d_HH,e_*	0.92	0.09	PA
				MARTINI_CERNS-Podewitz	320	46	40	*d_HH,e_*	1.06	0.05	PA
NS C24	MacDermaid	2020	[159]	SDK-CERNS	303		43	*d_V_*			PA
EOS	MacDermaid	2020	[159]	SDK-EOS	303		71	*d_V_*			PA
AP C24	Badhe	2020	[233]	MARTINI_CERAP-Badhe	305	47.9	51.7	*d_V_*	1.24	9–13	SA

a Bilayer thickness calculation methods are denoted as follows (see Section 3.2.3): *d_V_*, the total lipid volume of the bilayer divided by the cross-sectional area of the simulation box; *d*_*HH,e*_, the distance between the headgroup peaks in the electron density profile; and *d*_*WI*,1/*e*_, the distance between the lipid-water interfaces on either side of the bilayer defined as the location at which the mass density falls to 1/*e*. Reference atoms used in the *d*_*REF*_ method are listed in parentheses.

b Bilayer was pre-assembled (PA) or self-assembled (SA).

c Y Sugita, personal communication, email, 2 October 2020.

d Results are only presented for CER NS C16 and C24 although Paloncyova et al. studied CER NS C2, C4, C8, C12, C16, C20 and C24 by including none or up to six CG tail beads [152].

e CER NS C24 with three-bead headgroup mapping in which, similar to the MARTINI models shown in Fig. 9, the individual hydroxyl groups are not treated separate from the backbone structure as in the four bead headgroup mapping for MS-IBI shown in Fig. 10 [151].

f T. Moore, personal communication, 2022.

**Table 9:** Summary of results reported (area per lipid (APL), bilayer thickness, interdigitation, tilt angle and phase) at 300–310 K for CG simulations of ternary mixtures of CER NS C24, CHOL and FFA C24 for compositions that have been examined in more than one study. The CG results are compared with results from the equivalent all atom simulations.

Mole Ratio			Force Fields									
												
CER NS C24: CHOL: FFA C24	Author Year [Ref]	Temp (K)	CER NS C24	FFA C24	CHOL	Bilayer Simulation^a^	APL (Å^2^)	Bilayer Thickness (Å)^m^	Thickness Calculation Method^b^	Interdigitation (Å)^c^	Tilt angle (Deg)	Phase^d^
1:1:1	Gupta Group 2016–2019 [200,223-228]^e^	310	MARTINI_CERNS-Sovova	MARTINI_FFAC24^f^	MARTINI_CHOL^g^	PA: HBL						
	Podewitz 2018 [145,146]	300	MARTINI_CERNS-Podewitz	MARTINI_FFAC24	MARTINI_CHOL	SA: HBL	33.1^h^	50.7	*d*_*WI*,1/2_	7.0 CER		G
					MARTINI_CHOL_new_^i^	SA: HBL	34.1^h^	49.5	*d*_*WI*,1/2_	7.4 CER		LO
	Shamaprasad 2022 [110]	305	MSIBI_CERNS	MSIBI_FFA	MSIBI_CHOL	SA: HBL	33.8	48.3	*d*_*HH,m*_	10.5 All (10.1 CER 0 CHOL 10.1 FFA)	10	
						SA: 6-HML inner bilayer	34.3	51.4	*d*_*HH,m*_	10.2 All (9.9 CER 0 CHOL 10.6 FFA)	9	
	Wang 2018 [148]	305	All atom (CHARMM36-Wang)			PA: HBL	32.6	49.7, 51.4, 44.7	*d*_*HH,e*_, *d*_*WI*,1/2_, *d_FWHM_*	6.3 CER 0.88 CHOL		G
	Moore 2018 [115]	305	All atom (CHARMM36-Guo)			PA: HBL	32.0	51.8	*d*_*WI*,1/*e*_	10.6 FFA (8.1 All 7.3 CER 0.1 CHOL 8.7 FFA)	9	
1:0.5:1	Podewitz 2018 [145,146]	300	MARTINI_CERNS-Podewitz	MARTINI_FFAC24	MARTINI_CHOL	SA: HBL	34.1^h^	53.4	*d*_*WI*,1/2_	6.0 CER		G
					MARTINI_CHOL_new_	SA: HBL	34.4^h^	53.0	*d*_*WI*,1/2_	6.2 CER		G
	Shamaprasad 2022 [110]	305	MSIBI_CERNS	MSIBI_FFA	MSIBI_CHOL	PA: HBL	33.1	50.7	*d*_*HH,m*_	8.9 All (7.3 CER 0 CHOL 7.3 FFA)	8	
						SA: HBL	33.3	50.8 (54.4)	*d*_*HH,m*_ (*d*_*WI*,1/2_)	10.7 All (10.2 CER 0 CHOL 11.2 FFA)	8	
						SA: 6-HML inner bilayer	33.1	54.4	*d*_*HH,m*_	9.4 All (9.0 CER 0 CHOL 9.7 FFA)	9	
	Shamaprasad 2022 [110]	305	All atom (CHARMM36-Guo)			PA: HBL	30.4	52.7 (57.0)	*d*_*HH,m*_ (*d*_*WI*,1/2_)		8	
1:1:0.5	Wan 2015 [214]	310	MARTINI_CERNS-Lopez^j^	MARTINI_FFAC24_mod_^k^	MARTINI_CHOL	PA: HBL	36.0	43.3^h^	*d_REF_*			G
	Dai 2016 [215]	310	MARTINI_CERNS-Lopez^j^	MARTINI_FFAC24_mod_^k^	MARTINI_CHOL	PA: HBL	35.2^h^	43.9^h^	*d*_*HH,?*_^l^			
	Podewitz 2018 [145,146]	300	MARTINI_CERNS-Podewitz	MARTINI_FFAC24	MARTINI_CHOL	SA: HBL	34.9^h^	48.7	*d*_*WI*,1/2_	7.3 CER		G
					MARTINI_CHOL_new_	SA: HBL	35.5^h^	49.1	*d*_*WI*,1/2_	7.2 CER		LO

a Pre-assembled (PA) or self-assembled (SA) hydrated bilayer (HBL) or inner (central) bilayer of a 6-leaflet hydrated multilayer stack (6-HML).

b Bilayer thickness calculations as designated in Section 3.2.3: the distance between the headgroup peaks in the electron density profiles (*d*_*HH,e*_), the mass density profile (*d*_*HH,m*_), or unspecified profile (*d*_*HH,?*_); the distance between the lipid-water interfaces on either side of the bilayer defined as the location at which the mass density falls to 1/*x*, where *x* is either *e* (*d*_*WI*,1/*e*_) or 2 (*d*_*WI*,1/2_) (Wang [148] used electron density instead); the distance between the designated reference group (*d_REF_*) and the distance between half of the maximum peak values in the lipid electron density profile (*d_FWHM_*).

c Interdigitation values listed for the indicated lipid component (CER, CHOL, or FFA) or for all lipid components combined (All) were calculated as described in Section 3.2.5. Interdigitation results from Shamaprasad 2022 and Moore 2018 that are listed in parentheses were not reported previously; these new interdigitation results were calculated using trajectories from the simulations presented in each of these papers. Interdigitation results attributed to Podewitz 2018 are corrections (provided in reference [146]) to those listed in Table S4 of the supporting information for Podewitz 2018.

d Gel (G) and liquid ordered (LO) phases were identified in Podewitz 2018 using 2-dimensional RDFs, in combination with CER tail order parameters, compressibility modulus, and lateral diffusivity of the lipids. Wan 2015 states that the bilayer formed a gel (G) phase without specifying how this was known. Dai 2016 did not specify the bilayer phase, but did report that a phase transition based on APL occurs between 333 and 380 K.

e See footnote m in Table 7 for a description of the studies described in the seven papers from the Gupta group. Except for tail order parameters, no properties were reported for the bilayer without any additives.

f Standard MARTINI force field for FFA C24 (http://cgmartini.nl/images/parameters/lipids/FA/XCA/martini_v2.0_XCA_01.itp) [222].

g Original MARTINI force field for CHOL (http://cgmartini.nl/images/parameters/lipids/Sterols/CHOL/martini_v2.0_CHOL_01.itp) [222].

h Digitized from figure in paper using Grapher 16.7 (Golden Software LLC, Golden, CO).

i New MARTINI force field for CHOL (http://cgmartini.nl/images/parameters/lipids/Sterols/CHOL/martini_v2.0_CHOL_02.itp) [237].

j Two beads were added to extend the fatty acid tail from Lopez et al. [221] (available on the MARTINI website with the name CER at http://cgmartini.nl/images/parameters/lipids/CER/DPCE/martini_v2.0_CER.itp) to C24.

k Modification of the standard MARTINI force field for FFA C24 (http://cgmartini.nl/images/parameters/lipids/FA/XCA/martini_v2.0_XCA_01.itp) in which the head bead type is P3 (instead of P4), and the bond length and force constant between the head bead and the first bead of the fatty acid is 0.37 nm (instead of 0.47 nm) and 20,000 kJ mol^−1^ nm^−2^ (instead of 1250 mol^−1^ nm^−2^), respectively.

l Dai 2016 did not specify the method for calculating bilayer thickness; the method is most likely the same as that used in Wan 2015, which is also from the Shi group (see Table 7). Wan 2015 defined bilayer thickness as the peak-to-peak distance of hydrophilic headgroups (specific CG beads are not identified) in an unspecified density profile, most probably mass.

m Results listed in parentheses were not reported previously; these are presented here for comparing with results from Podewitz 2018 [145,146].

## Footnotes

2 MacDermaid et al. incorrectly report 9.8 nm as the experimental repeat distance for pure CER EOS; 9.8 nm is the repeat distance for one of the two phases observed in the 33% CHOL mixture [159].

3 The 1-Å thick water layer that Badhe et al. [233] estimated for each leaflet (i.e., a 2-Å thick water layer between bilayer headgroups) appears to have been calculated by incorrectly using the number of water beads per leaflet (401) as the number of water molecules per leaflet (which is 4×401 = 1604).

## Abbreviations:

AA: all atom
APL: area per lipid
APT: area per tail
CER XY Cn: ceramide
X = A: alpha hydroxy fatty acid
B: beta hydroxy fatty acid
EA: alpha hydroxy fatty acid ester linked to sphingosine
EN: non-hydroxy fatty acid ester linked sphingosine
EO: esterified omega hydroxyl
N: non-hydoxy
O: omega hydroxy
Y = dS: dihydrosphingosine
H: 6-hydroxysphingosine
P: phytosphingosine
S: sphingosine
SD: 4,14-sphingadiene
T: dihydroxy dihydrosphingosine
n: number of carbons in fatty acid tail
CHOL: cholesterol
CholSO4: cholesterol sulfate
CG: coarse-grained
CPE: chemical penetration enhancer
*d_HH,x_*: bilayer thickness as distance between headgroup peaks of *x*
*e*: electron density profile
*m*: mass density profile
*n*: neutron scatter length density (NSLD) profile
*d_V_*: bilayer thickness as total lipid volume divided by area
*d*_*WI*,1/*x*_: bilayer thickness as the distance between the lipid-water interfaces on either side of the bilayer defined as the location at which the mass or electron density falls to 1/*x* where *x* is either *e* or 2
DL: dehydrated lipids in random configuration
DPPC: dipalmitoylphosphatidylcholine
DSC: Differential scanning calorimetry
DOPC: Dioleoylphosphatidylcholine
EX: CER in extended conformation
FFA: free fatty acid
FFA5 and FFA7: 5 and 7-component mixture of free fatty acids
FFA Cn: free fatty acid with n carbons
FTIR: Fourier transfer infrared spectroscopy
HBL: hydrated bilayer
n-HBL: n hydrated bilayer stack
HP: CER in hairpin conformation
IBI: Iterative Boltzmann inversion
LC/MS: Liquid chromatography/mass spectrometry
LPP: long periodicity phase
LWmix: lipids and water mixed in random configuration
MC: Monte Carlo; MD, molecular dynamics
MLP: molecular lipophilicity potential
MS-IBI: multi-state iterative Boltzmann inversion
MW: molecular weight
n-DML: n-leaflet dehydrated multilayer stack
n-HML: n-leaflet hydrated multilayer stack
NLA: normalized lipid area
NMR: Nuclear magnetic resonance
NPT: constant pressure, constant temperature ensemble
NVT: constant volume, constant temperature ensemble
NSLD: neutron scattering length density
PA: pre-assembled
PABA: p-amino-benzoic acid
RDF: radial distribution function
*S_CC_, S_CD,_S_CH,_S*_2_: order parameter: carbon-carbon, carbon-deuterium, carbon-hydrogen, nematic
SA: self-assembled
SAXD: Small angle X-ray diffraction
SC: stratum corneum
SCS: stratum corneum substitute
SDK: Shinoda-DeVane-Klein force field
SPP: short periodicity phase
TLC: Thin layer chromatography
UA: united atom
VPL: volume per lipid
WLW: randomly configured lipid layer between two water layers
