# Supplementary material for: Using molecular simulation to understand the skin barrier
Source: Prog Lipid Res. Author manuscript; Available in PMC 2023 Apr 20. (PMC10116345; doi:10.1016/j.plipres.2022.101184)
Supplement: Supplementary material [file NIHMS1890746-supplement-Supplementary_material.pdf]

Supplementary Information for the paper:

Using Molecular Simulation to Understand the Skin Barrier

Parashara Shamaprasad,[1,2] Chloe O. Frame,[1,2] Timothy C. Moore†,[1,2] Alexander Yang,[1,2] Christopher R. Iacovella,[1,2] Joke A. Bouwstra,[3] Annette L. Bunge,[4] and Clare McCabe[1,2,5]

[1]Department of Chemical and Biomolecular Engineering, Vanderbilt University, Nashville, TN, 37235-1604

[2]Multiscale Modeling and Simulation (MuMS) Center, Vanderbilt University, Nashville, TN, 37235-1604

[3]Division of BioTherapeutics, LACDR, Leiden University, 2333 CC Leiden, The Netherlands

[4]Department of Chemical and Biological Engineering, Colorado School of Mines, Golden, CO, 80401

[5]Department of Chemistry, Vanderbilt University, Nashville, TN, 37235-1604

†Current Address: Department of Chemical Engineering, University of Michigan, Ann Arbor, MI, 48109-1382

S1. Ceramide subclasses observed in human SC

Ceramides (CER) consist of a sphingoid base linked to a fatty acid by an amide bond. The various CER subclasses in SC are identified as CER $Z_{FA}Z_{SB}$, where Z_{FA} and Z_{SB} designate by one or two letters the constituent fatty acid and sphingoid base, respectively [1]. Five fatty acids containing three different head groups have been identified in human SC. Fatty acids with the non-hydroxy, alpha-hydroxy, or beta-hydroxy headgroup connected to a straight hydrocarbon chain, which is usually fully saturated, are identified as N, A and B, respectively. The omega hydroxy (designated as O) fatty acid has a non-hydroxy headgroup and a hydroxy group on the terminal carbon (i.e., the ω position) of a straight hydrocarbon chain. In the fifth fatty acid, identified as EO, the O fatty acid is ester linked at the ω position to linoleic acid [2,3]. The sphingoid base, with the Z_{SB} designations listed in the parentheses, can be sphingosine (S), phytosphingosine (P), 6-hydroxysphingosine (H), dihydrosphingosine (dS), or 4,14-sphingadiene (SD) [3]. Thus, CER NS denotes a non-hydroxy fatty acid linked to a sphingosine base. To date, only 21 of the 25 possible combinations of the five fatty acids and five sphingoid bases have been observed in human SC: only BS has been detected so far; BP; BH, BdS and BSD have not been identified [3]. Three other CERs have been detected, which brings the total to 24. One of these, CER NT, consists of sphingoid base, dihydroxy-dihydrosphingosine (T), containing four hydroxy groups (i.e., one more than the P or H sphingoid base) in a structure that is not exactly known at present. So far, it has only been identified in combination with the N fatty acid (NT) [4]. Two other CERs contain three tails, in which the non-hydroxy fatty acid is ester-linked to the primary hydroxyl of a sphingosine (S) base to make 1-O-acylsphingosine that is connected through the amide bond to either the non-hydroxy (N) or the alpha-hydroxy (A) fatty acid. The 1-O-acylsphingosine base was discovered and designated 1-OE by Rabionet et al. [5]; it was subsequently simplified to E_S [2], and the two CERs observed in human SC designated as CER ENS and CER EAS. In healthy SC, the fatty acid chains of the CERs are generally saturated and long, usually 16-32 carbon atoms with 24-28 being most common, except for the O fatty acid chains, which are longer (up to 38 carbon atoms) with 30-32 carbon atoms being most common [2,4,6-8]. Figure S1 shows the structures and nomenclature for the 24 CER subclasses that have been identified in human SC.

**Fatty Acid (Z_{FA})** / **Sphingoid Base (Z_{SB})**	Non-hydroxy (N) 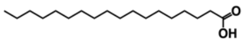	α-hydroxy(A) 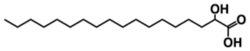	β-hydroxy (B) 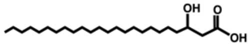
Sphingosine (S) 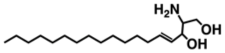	NS 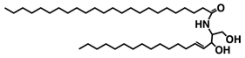	AS 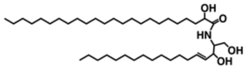	BS 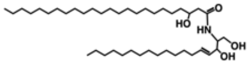
Phytosphingosine (P) 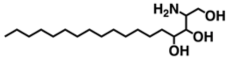	NP 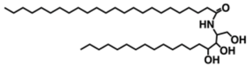	AP 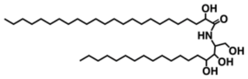	BP not observed
6-hydroxy sphingosine (H) 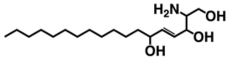	NH 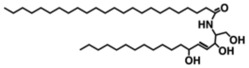	AH 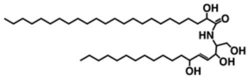	BH not observed
Dihydrosphingosine (dS) 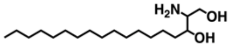	NdS 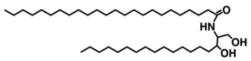	AdS 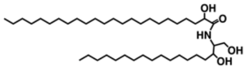	BdS not observed
4, 14-sphingadiene (SD) 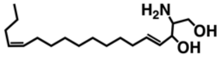	NSD 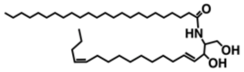	ASD 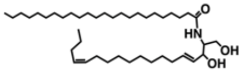	BSD not observed
Dihydroxysphinganine (T) 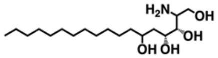	NT 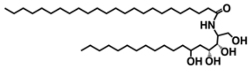	AT not observed	BT not observed
1-O-Acylsphingosine (E_S) 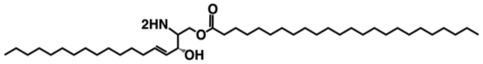	ENS 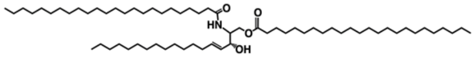	EAS 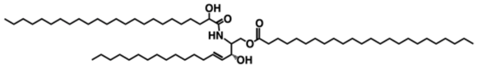	

Figure S1. Structure and nomenclature for the 24 CER subclasses identified in the unbound lipids from human SC. Two-tailed CERs are designated as CER $Z_{FA}Z_{SB}$ where Z_{FA} and Z_{SB} represent by one or two letters the fatty acid and sphingoid base, respectively. Three-tailed CERs are designated as CER E_S where E designates a non-hydroxy fatty acid ester-linked to the primary hydroxyl of the sphingosine base (S), which is connected by an amide bond to a second fatty acid, either the non-hydroxy (N) or alpha-hydroxy (A) fatty acid. The possible structure shown for dihydroxy sphinganine (T) is from Schmitt and Neubert [2].

**Fatty Acid (Z_{FA})** / **Sphingoid Base (Z_{SB})**	ω-hydroxy (O) 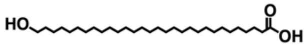	Esterified ω-hydroxy (EO) 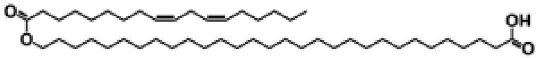
Sphingosine (S) 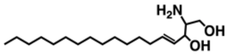	OS 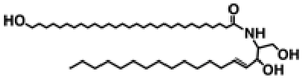	EOS 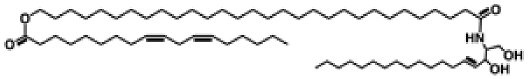
Phytosphingosine (P) 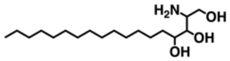	OP 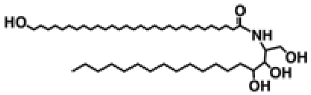	EOP 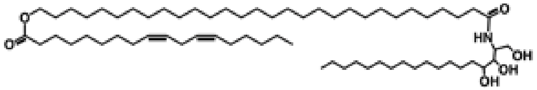
6-hydroxy sphingosine (H) 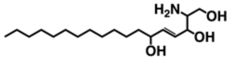	OH 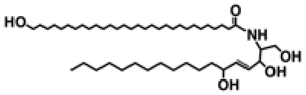	EOH 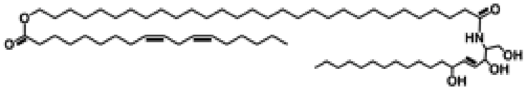
Dihydrosphingosine (dS) 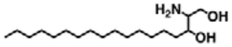	OdS 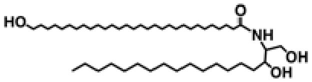	EOdS 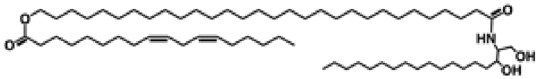
4, 14-sphingadiene (SD) 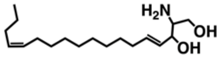	OSD 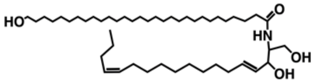	EOSD 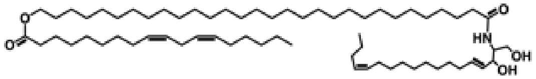

Figure S1 (continued). Structure and nomenclature for the 24 CER subclasses identified in the unbound lipids from human SC. Two-tailed CERs are designated as CER $Z_{FA}Z_{SB}$ where Z_{FA} and Z_{SB} represent by one or two letters the fatty acid and sphingoid base, respectively. Three-tailed CERs are designated as CER E_S where E designates a non-hydroxy fatty acid ester-linked to the primary hydroxyl of the sphingosine base (S), which is connected by an amide bond to a second fatty acid, either the non-hydroxy (N) or alpha-hydroxy (A) fatty acid. The possible structure shown for dihydroxy sphinganine (T) is from Schmitt and Neubert [2].

S2. Ceramide compositions in pig and human SC

Tables S1 – S5 provide the published compositions for CERs in pig and human SC determined using either thin layer chromatography (TLC) or liquid chromatography with mass spectrometry (LC/MS). Compositions in weight % are converted to mole % using the molecular weight (MW) values listed in Table S1, which are copied from Schmitt and Neubert [2].

Table S1. CER compositions reported for SC lipids extracted from pigs and molecular weight values used to calculate the listed mole % compositions

	Measured CER composition (weight %) [a]				Calculated CER composition (mole %)				MW [b]
First author	Wertz	Law [c]	Bouwstra	Caussin	Wertz	Law	Bouwstra	Caussin	Schmitt
Year	1983	1995	1996	2008	1983	1995	1996	2008	2018
Ref No.	[9]	[10]	[11]	[12]					[2]
Source in paper	Table 5	Table 1	Table 1	Section 2.2					Table 1
NS [d]	42.4	36.5	55.4	64	43.3	38.0	56.2	66.5	677.69
NP	10.2	11.8	17.6	8	10.1	12.0	17.4	8.1	695.7
NH									693.69
NdS									679.7
AS [e]	12.1	9.6	3.6	6	12.1	9.8	3.6	6.1	693.69
AS C16	10.5	8.4	9.9	4	13.1	10.7	12.3	5.1	553.69
AP	15.5	21.3	5.6	6	15.1	21.1	5.4	5.9	711.7
AH									709.69
AdS									677.69
EOS	9.4	12.4	7.8	12	6.3	8.5	5.2	8.2	1025.98
EOP									1043.99
EOH									1041.98
EOdS									1027.99
Total	100.1	100.0	99.9	100.0	100.0	100.1	100.1	99.9	
EO total	9.4	12.4	7.8	12.0	6.3	8.5	5.2	8.2	

[a] CER compositions were determined by thin layer chromatography (TLC).

[b] Molecular weight (MW) values used to convert compositions in weight % to mole % are from Schmitt and Neubert [2] except for AS C16. Values account for differences in the CER head groups while assuming an average 67 carbons for all EO-type CERs and 44 carbons for all non-EO CERs, except for AS C16, assumed to have 34 carbons. Thus, mole % compositions derived from weight % results will not reflect different chain lengths among the CERs; e.g., the CER AS fatty acid chain is typically shorter than fatty acid chains of other non-EO CERs [3].

[c] Compositions in weight % were calculated from data reported as μg/mg of dry SC (NS: 25.1, NP: 8.1, AS: 6.6, AP: 14.6, EOS: 8.5, AS C16: 5.8).
[d] NS includes NdS
[e] AS includes AdS

Table S2. CER compositions for lipids extracted from human SC that were reported as weight % [a]

Analysis method	TLC							LC/MS
Skin source [b]	Forearm [c]<br>n = 4	Surgical waste	Arm<br>n = 10	Breast (surgical waste)	Forearm scraping<br>n = 5	Breast (surgical waste)<br>n = 2	Surgical waste	Forearm tape strips<br>n = 7
First author	Lavrijsen	Vicanova	Bleck	Weerheim	Weerheim	Ponec	Caussin	Ishikawa
Year	1994	1998	1999	2001	2001	2003	2008	2010
Ref No.	[13]	[14]	[15]	[16]	[16]	[17]	[12]	[18]
Source in paper	Fig. 1 [d]	Table II	Table III (for NSK)	Table 2	Table 2	Fig. 1 [e]	Section 2.3	Fig. 1e [d]
NS [g]	24	22.7	21	25.1	22.9	20.6	28	7.3
NP	21	20.2	19	26.5	18.4	18.1	28	18.0
NH								19.6
NdS								5.8
AS [h]	20	21.5	17	24.2	21.3	19.8	18	5.7
AP	6	7.5	10	5.7	6.3	8.7	9	18.9
AH	12	14.7	14	9.8	15.6	13.0	5	16.7
AdS								1.2
EOS	10		10	5.8	8.6	8.4	5	4.5
EOP [i]						6.4	3	0.7
EOH	7	6	9	2.9	7.1	5.0	4	1.9
EOdS [j]								
Total	100	99.2	100	100.0	100.2	100.0	100	100.3
EO total	17	12.6	19	8.7	15.7	19.8	12	7.1

[a] CER compositions were determined by thin layer chromatography (TLC) or liquid chromatography/mass spectrometry (LC/MS).

[b] Body region of SC sampled; n = number of subjects included in the reported average when n was reported.

[c] Lipids were extracted into acetone/diethylether applied to the forearm

[d] As reported in Table 2 of Weerheim and Ponec [16].

[e] Reported compositions (NS: 20.5, NP: 18.0, AS: 19.7, AP: 8.6, AH: 12.9, EOS: 8.3, EOP: 6.4, EOH: 5.0) summed to 99.4%. Numbers listed here have been normalized to give 100.0%.

[f] Data reported in Fig. 3e of Ishikawa et al. [18] as ng/μg of protein are reported in Table 1 of Kovacik et al. [19] as weight %, which are the numbers listed here. Kawana et al. [3] repeated the numbers from Kovacik et al. [19] in Table S6 without specifying weight %.

[g] NS and NdS were not determined separately in the TLC analyses

[h] AS and AdS were not determined separately in the TLC analyses

[i] CER EOP was not identified in human SC until 2003 [17]

[j] CER EOdS was not identified in human SC until 2011 [20]

Table S3. CER compositions in mole % for lipids extracted from human SC that were reported as weight % [a]

Analysis method	TLC							LC/MS
Skin source [b]	Forearm n = 4	Surgical waste	Arm n = 10	Breast (surgical waste)	Forearm scraping n = 5	Breast (surgical waste) n = 2	Surgical waste	Forearm tape strips n = 7
First author	Lavrijsen	Vicanova	Bleck	Weerheim	Weerheim	Ponec	Caussin	Ishikawa
Year	1994	1998	1999	2001	2001	2003	2008	2010
NS [c]	26.0	24.5	23.0	26.4	24.7	22.6	29.8	7.7
NP	22.2	21.2	20.2	27.2	19.3	19.3	29.0	18.4
NH								20.1
NdS								6.1
AS [d]	21.2	22.6	18.2	24.9	22.4	21.2	18.7	5.9
AP	6.2	7.7	10.4	5.7	6.5	9.1	9.1	18.9
AH	12.4	15.1	14.6	9.8	16.0	13.6	5.1	16.8
AdS								1.3
EOS	7.2	4.7	7.2	4.0	6.1	6.1	3.5	3.1
EOP [e]						4.6	2.1	0.5
EOH	4.9	4.2	6.4	2.0	5.0	3.6	2.8	1.3
EOdS [f]								
Total	100.1	100.0	100.0	100.0	100.0	100.1	100.1	100.1
EO total	12.1	8.9	13.6	6.0	11.1	14.3	8.4	4.9

[a] Mole % numbers were calculated from weight % numbers listed in Table S2 using the molecular weight (MW) values from Table S1.

[b] Body region of SC sampled; n = number of subjects sampled if provided.

[c] NS and NdS were not determined separately in the TLC analyses

[d] AS and AdS were not determined separately in the TLC analyses

[e] CER EOP was not identified in human SC until 2003 [17]

[f] CER EOdS was not identified in human SC until 2011 [20]

Table S4. CER compositions from Masukawa 2009 [21] for lipids extracted from human SC [a]

Analysis method	TLC						LC/MS					
units	ng/μg protein		wt % [b]		wt % [c,d]	mol % [e]	ng/μg protein		wt % [b]		wt % [d,f]	mol % [e,f]
	A	B	A	B	Average	Average	A	B	A	B	Average	Average
NS	4.53	3.16	10.69	9.98	10.34	10.96	2.76	2.03	6.36	6.34	6.35	6.70
NP	13	5.77	30.69	18.22	24.46	25.26	11.5	5.16	26.49	16.12	21.30	21.91
NH	7.94	7.63	18.74	24.10	21.42	22.19	9.47	7.47	21.81	23.34	22.57	23.28
NdS [g]							3.02	1.58	6.96	4.94	5.95	6.26
AS	2.35	1.8	5.55	5.69	5.62	5.82	1.15	1.43	2.65	4.47	3.56	3.67
AP	5.46	5.44	12.89	17.18	15.04	15.18	5.9	5.96	13.59	18.62	16.10	16.19
AH	5.01	5.24	11.83	16.55	14.19	14.37	5.49	6.02	12.64	18.81	15.73	15.85
AdS [h]				0.00			0.38	0.24	0.88	0.75	0.81	0.86
EOS	1.57	1.62	3.71	5.12	4.41	3.09	1.79	1.42	4.12	4.44	4.28	2.98
EOP	0.8	0.13	1.89	0.41	1.15	0.79	0.62	0.09	1.43	0.28	0.85	0.59
EOH	1.7	0.87			3.38	2.33	1.34	0.61	3.09	1.91	2.50	1.71
EOdS [i]												
Total	42.36	31.66	100.00	100.00	100.00	99.99	43.42	32.01	100.00	100.00	100.0	100.00
EO total	4.07	2.62	9.61	8.28	8.94	6.21	3.75	2.12	8.64	6.62	7.63	5.28

[a] CER masses determined using thin layer chromatography (TLC) and liquid chromatography/mass spectrometry (LC/MS) of forearm SC collected on tape strips from subjects A and B are reported in Table 5 of Masukawa et al. 2009 as ng/μg protein.

[b] Weight % numbers are the ratio of CER mass per protein to the total CER mass per protein.

[c] These same calculated average weight % numbers are listed in Table 1 of Kovacik et al. [19] with minor round-off differences except for 10.0% for EOS; Kovacik incorrectly specifies that these results were determined by LC/MS. Kawana et al. [3] repeated the numbers from Kovacik et al. [19] in Table S6 without specifying weight % or mole %.

[d] The calculated average weight % numbers listed in Table 1 of van Smeden et al. [6] differ slightly from those listed here; the numbers listed in van Smeden et al. were calculated as the ratio of the average mass per protein from subjects A and B for each CER to the average mass per protein from subjects A and B for the CER total (e.g., the weight % of CER AP determined by TLC = 100 [(5.46 + 5.44)/2]/[(42.36 + 31.66)/2] = 14.7%).

[e] Mole % numbers were calculated from the weight % data using the molecular weight (MW) values listed in Table S1.

[f] These same calculated weight % numbers are listed in Table 1 of Schmitt and Neubert [2]; however, the mole % numbers listed in their Table 1 were calculated incorrectly.

[g] NS and NdS were not determined separately in the TLC analyses

[h] AS and AdS were not determined separately in the TLC analyses

[i] CER EOdS was not identified in human SC until 2011 [20]

Table S5. CER compositions reported in mole % for SC lipids extracted from humans and determined by LC/MS

Skin source [a]	Forearm tape strips n = 7	Forearm tape strips n = 15	Forearm tape strips		Forearm tape strips n = 19		Forearm tape strips n = 5	
First author	Janssens	Janssens	t'Kindt		Kawana		van Smeden	
Year	2011	2012	2012		2020		2020	
Ref No.	[22]	[23]	[4]		[3]		[24]	
Source in paper	Fig. 2a [b]	Supplementary Table III [c]	Fig. 4 [d]		Table S2		Fig. 6 [e]	
	As reported	As reported	As reported	Calculated for 12 CERs [f]	As reported	Calculated for 12 CERs [f]	As reported	Normalized to 100%
NS	7.8	6.88	7.44	7.68	5.16	5.27	5.2	5.2
NP	28.9	26.50	22.10	22.80	24.21	24.72	26.6	26.8
NH	13.9	14.01	14.51	14.97	23.74	24.24	16.3	16.4
NdS	9.0	9.48	9.83	10.14	6.17	6.30	11.4	11.5
AS	4.6	4.57	9.58	9.88	4.29	4.38	2.9	2.9
AP	15.7	14.79	8.78	9.06	9.16	9.35	13.3	13.4
AH	12.5	13.07	10.77	11.11	17.96	18.34	13.2	13.3
AdS	1.0	1.09	1.63	1.68	0.91	0.93	1.7	1.7
EOS	2.9	3.76	6.48	6.69	2.11	2.15	3.7	3.7
EOP	1.0	1.43	1.14	1.18	1.03	1.05	1.5	1.5
EOH	2.6	4.06	4.26	4.40	3.10	3.17	3.2	3.2
EOdS	0.2	0.37	0.40	0.41	0.10	0.09	0.4	0.4
NSD					0.13			
ASD					0.15			
EOSD					0.02			
OS			0.73		0.56			

OP			0.17		0.33			
OH			0.43		0.62			
OdS					0.07			
OSD					0.02			
BS					0.17			
NT			1.73					
Total	100.1	100.01	99.98	100.00	99.98	100.0	99.4	100.0
EO total	6.7	9.62	12.28	12.68	6.36	6.46	8.8	8.8

[a] Body region of SC sampled; n = number of subjects included in the reported average when n was reported.

[b] Results were reported as relative ceramide abundance (in percentage) of all CER subclasses measured (i.e., mol %). The numbers listed here are from Table 1 of Kovacik et al. [19] (reported incorrectly as weight %), which they determined by digitizing Fig. 2a (personal communication with K Vavrova by email 25 June 2020).

[c] Data from this study are also reported in papers from van Smeden et al. as % relative abundance (mole %) in Fig. 3b of reference [25] and as weight % in Table 1 of reference [6] (although reference [6] did not specify the units, and the amounts for AdS, and EOdS, and possibly EOP, are larger than expected, perhaps due to typographic or copying errors). The mole % numbers from van Smeden et al. [25] are also listed in Table S6 of Kawana et al. [3] without specifying mole %. Table 1 of Schmitt and Neubert [2] reports weight % numbers derived from the data listed in Table 1 of van Smeden et al. 2014[6], which they adjusted to 100% after excluding EOdS (1.3%). Schmitt and Neubert [2] calculated the mole % numbers they included in Table 1 of their paper using molecular weight (MW) values listed in their paper (and presented here in Table S1). We recommend using the mole % numbers presented here, which are from Janssens et al. [23]

[d] Although not stated in the paper, the results were reported as relative ceramide abundance (in percentage) of all CER subclasses measured (i.e., mol %); personal communication with the corresponding author K Sandra (email 02 September 2020). These same numbers are also listed in Table S6 of Kawana et al. [3] and in Table 1 of Schmitt and Neubert [2]. Kawana et al. did not specify mole % or weight %. Schmitt and Neubert incorrectly assumed weight % and therefore the mole % numbers listed in Table 1 of their paper are incorrect. Table 1 of Kovacik et al. [19] tabulates these same numbers for only the 12 most abundant CERs (i.e., without OS, OP, OH and NT), which sum to 96.9% (and not 100%); Kovacik et al. [19] incorrectly specify that the results are weight %.

[e] Results were reported as relative ceramide abundance (in percentage) of all CER subclasses measured (i.e., mol %); this totals to 99.4%.

[f] Calculated mole % of the 12 most abundant CERs.

S3. Comparison of simulations for bilayers with different SC lipid compositions

For systems with different lipid compositions, comparisons of area per lipid (APL) or normalized lipid area (NLA) should be performed using data from one study or between studies that used the same force field and computational protocol. Moore et al. [26] and Wang and Klauda [27-29] each generated simulation results, summarized in Table S6, of hydrated bilayers with other lipid compositions (i.e., CER NS C16, CER NS C24, and CER AP C24 alone or mixed with different amounts of CHOL and FFA C24) that can be compared. Table S7 compares APL and NLA for simulated bilayers of pure CER and equimolar mixtures of the same CER with CHOL and FFA C24. NLA values in these tables were calculated assuming the effective number of hydrocarbon tails per lipid is one for FFAs, two for CERs, and 1.9 for CHOL as proposed by Shamaprasad et al. [30].

Several observations can be made based on the results presented in Tables S6 and S7. First, adding CHOL to CER bilayers causes a minimal change in the APL; see results from Moore et al. [26] for CER NS. This is expected given that CER and CHOL have similar cross-sectional areas [31]. Second, as also expected, the addition of FFA, which has a single hydrocarbon tail, to either pure CER or CER-CHOL mixtures decreases the APL significantly with almost no effect on the NLA. Third, changes in the CER NS acyl tail length from C16 to C24 have no effect on the APL whether the bilayer consists of pure CER or a mixture of CER with CHOL or with both CHOL and FFA C24. Fourth, from the Wang and Klauda results listed in Table S7 [27-29], CER AP C24 has a larger APL compared with pure CER NS C24 (46.4 $Å^2$ and 42.8 $Å^2$), whereas the APL for equimolar mixtures of each with CHOL and FFA C24 are nearly identical (32.6 $Å^2$ and 32.8 $Å^2$, respectively), suggesting that the steric hindrance caused by the additional hydroxyls in the CER AP headgroup is mitigated by the presence of CHOL and FFA C24. Moreover, this effect is observed even when smaller amounts of equimolar CHOL and CER are added to the CER (Table S6); e.g., the APL values for AP and NS are identical for a CER:CHOL:FFA C24 mole ratio of 1:0.5:0.5 and are only slightly different for a mole ratio of 1:0.21:0.21 (37.4 $Å^2$ and 36.8 $Å^2$ for AP and NS, respectively). Consistent with the APL observations, the NLA values are essentially the same (~20 $Å^2$) for both CER AP C24 and CER NS C24 with mole ratios of 1:X:X CER:CHOL:FFA where X = 0.2, 0.5 and 1 (Table S6).

Table S6. Area per lipid (APL) and normalized lipid area (NLA) derived from atomistic simulations of hydrated bilayers described by Moore et al. and Wang and Klauda for CER NS and CER AP containing different amounts of CHOL and FFA C24 [a]

	NS C16	NS C24	AP C16	AP C24	CHOL	FFA C24	APL (Å^2)	NLA (Å^2)
Moore et al. [26]								
		1					39.0	19.5
		1			0.5		39.0	19.8
		1			1		39.0	20.0
		1			1	1	32.0	19.6
	0.25	0.75					39.0	19.5
	0.25	0.75			0.5		39.0	19.8
	0.25	0.75			1		39.0	20.0
	0.25	0.75			1	1	31.0	19.0
	0.5	0.5					38.0	19.0
	0.5	0.5			0.5		39.0	19.8
	0.5	0.5			1		39.0	20.0
	0.5	0.5			1	1	32.0	19.6
	0.75	0.25					40.0	20.0
	0.75	0.25			0.5		39.0	19.8
	0.75	0.25			1		39.0	20.0
	0.75	0.25			1	1	32.5	19.9
	1						40.0	20.0
	1				0.5		39.0	19.8
	1				1		39.0	20.0
	1				1	1	32.0	19.6
Wang and Klauda								
[28,29]	1						43.6	21.8
[28]	1				1	1	33.0	20.2
[28,29]		1					42.8	21.4
[27]		1			0.21	0.21	36.8	20.1
[27]		1			0.5	0.5	34.5	20.0
[27,28]		1			0.97	0.97	32.8	20.0
[27]		1			2	2	31.2	20.0
[29]				1			46.4	23.2
[27]				1	0.21	0.21	37.4	20.4
[27]				1	0.5	0.5	34.5	20.0
[27]				1	0.97	0.97	32.6	19.9
[27]				1	2	2	31.2	20.0
[29]			1				45.6	22.8

[a] Moore et al. used the CHARMM36-Guo force fields and Wang and Klauda used CHARMM36-Wang; all simulations were at 305 K

Table S7. Area per lipid (APL) and normalized lipid area (NLA) derived from atomistic simulations of hydrated bilayers described by Moore et al. and Wang and Klauda for CER NS and CER AP either alone or in equimolar mixtures with CHOL and FFA C24 [a]

System	CER	**Moore et al.** [26]		**Wang and Klauda** [27-29]	
		**APL (Å^2)**	**NLA (Å^2)**	**APL (Å^2)**	**NLA (Å^2)**
Pure CER	NS C24	39.0	19.5	42.8	21.4
	NS C16	40.0	20.0	43.6	21.8
	AP C24			46.4	23.2
	AP C16			45.6	22.8
Equimolar CER:CHOL:FFA C24	NS C24	32.0	19.6	32.8	20.0
	NS C16	32.0	19.6	33.0	20.2
	AP C24			32.6	19.9

[a] Moore et al. used the CHARMM36-Guo force field and Wang and Klauda used the CHARMM36-Wang force field; all simulations were performed at 305 K

.

S4. Analysis of SC permeation predictions from Gajula et al.

Gajula et al. [32] assumed, like many others before them, that permeation through the SC can be represented as Fickian diffusion across a brick-and-mortar structure in which the corneocytes are the bricks and the lipid layers surrounding the corneocytes are the mortar. Gajula et al. assumed further that the corneocytes were impermeable and, although not stated explicitly, diffusion in the lipid layers is isotropic [32]. Mathematically, this scenario is described by Eq. (S.1) for two dimensions

$$\frac{\partial C}{\partial t} = D_{lip}\left(\frac{\partial^2 C}{\partial x^2} + \frac{\partial^2 C}{\partial z^2}\right) \tag{S.1}$$

where C is the concentration and D_{lip} is the diffusion coefficient of the permeant in the lipids, t is time, and x and z are the coordinate directions parallel and perpendicular to the SC surface, respectively. In their calculations, Gajula et al. used the diffusion coefficient calculated from molecular simulations for D_{lip} [32].

Because the corneocytes have zero permeability, there is no flux in the direction normal to the surface of each corneocyte. Thus,

$$\frac{\partial C}{\partial \vec{n}} = 0 \tag{S.2}$$

at the surface of all corneocytes. As a result, a numerical solution such as the finite element method is required to solve Eq. (S.1).

However, Kushner et al. [33] showed that this two-dimensional description of diffusion in the SC lipids surrounding impermeable corneocytes could be represented by the following one-dimensional expression

$$\frac{\partial C}{\partial t} = \frac{D_{lip}}{\tau_{flux}\tau_{volume}}\frac{\partial^2 C}{\partial z^2} \tag{S.3}$$

in which τ_{volume} and τ_{flux} are tortuosity factors accounting for the total volume of the branched, parallel transport pathways in the lipid domain of the SC (τ_{volume}) and the longer diffusion pathway due to lateral diffusion around the corneocytes (τ_{flux}). Thus, according to Eq. (S.3), permeation through a SC with impermeable corneocytes is equivalent to permeation through a homogeneous membrane, but with an *effective* diffusion coefficient (D_{eff}) that is reduced compared with D_{lip} by these tortuosity factors as specified in Eq. (S.4)

$$D_{eff} = \frac{D_{lip}}{\tau_{flux}\tau_{volume}} \tag{S.4}$$

Kushner et al. [33] demonstrated for various brick-and-mortar geometric representations of the SC that the cumulative mass transfer calculated from finite element numerical solutions of Eq. (S.1) matched those calculated from the analytical solution of Eq. (S.3) for the same conditions; i.e., no permeant in the SC at t = 0, sink conditions on the inside surface of the SC, and constant permeant concentration on the SC surface for t > 0.

However, instead of using solutions to either Eq. (S.1) or Eq. (S.3), Gajula et al. used a finite element method to solve Eq. (S.5)[32]

$$\frac{\partial C}{\partial t} = \frac{D_{lip}}{\tau_{flux}\tau_{volume}}\left(\frac{\partial^2 C}{\partial x^2} + \frac{\partial^2 C}{\partial z^2}\right) \quad \text{(S.5)}$$

which combines Eqs. (S.1) and (S.3), for their assumed corneocyte-lipid geometry (see Figure S2 and Table S8). As a result, their calculations for transport across the SC have accounted for the impermeable corneocytes and extended lipid pathway twice. Figure S3 shows the cumulative mass transfer per area (Q) versus time they calculated compared with experiments for caffeine, fentanyl and naphthol (reproduced by digitizing the plots presented in their Figure 6 [32]).

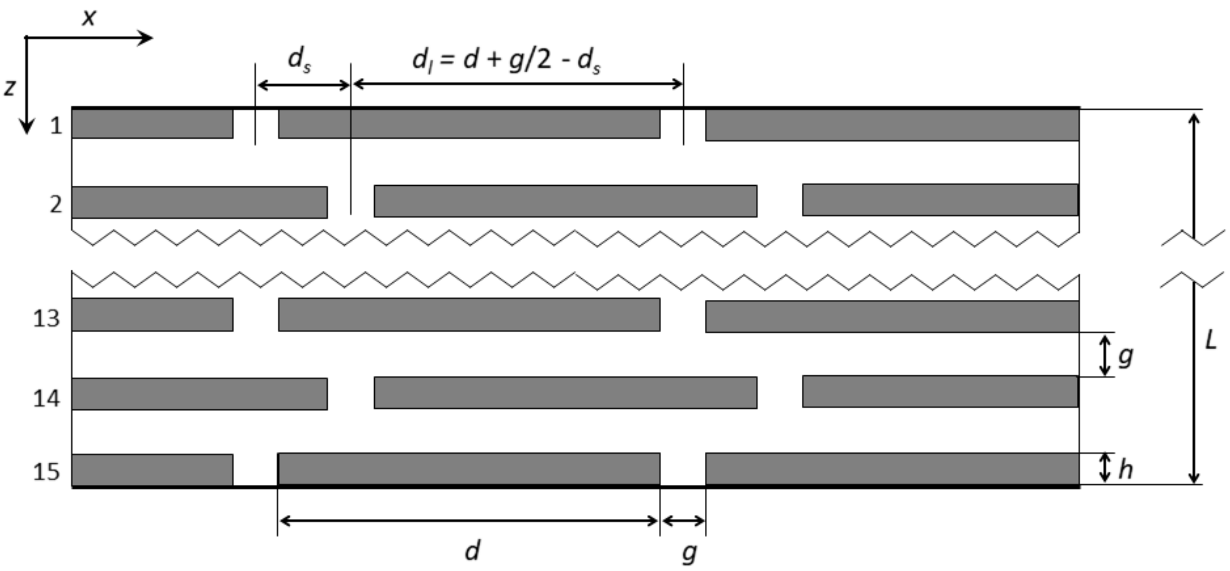

Figure S2. Schematic diagram of the brick-and-mortar configuration Gajula et al. [32] assumed in their calculations (see Table S8 for parameter descriptions and numerical values).

We determined the D_{eff} values in the Gajula et al. [32] calculations by fitting their Q versus t curves to the following expression

$$Q = J_{ss}\left(t - t_{lag}\right) - \frac{12 J_{ss}\, t_{lag}}{\pi^2}\sum_{n=1}^{\infty}\frac{(-1)^n \exp\left[-n^2\pi^2\, t/\left(6\, t_{lag}\right)\right]}{n^2} \quad \text{(S.6)}$$

which describes Q for a permeant that penetrates a homogeneous membrane with a steady-state flux (J_{ss}) and lag time (t_{lag}) where t_{lag} is related to D_{eff} and the SC thickness (L) as

$$t_{lag} = L^2/\left(6 D_{eff}\right) \quad \text{(S.7)}$$

Table S8. Descriptions, numerical values, and defining equations for the parameters used by Gajula et al. [32] in their calculations of *Q* versus *t* (see Figure S2 schematic)

Parameter	Description	Value used	Defining equation
d	corneocyte width (μm)	40	
d_s	shorter lateral diffusion path around a corneocyte	varied	
d_l	longer lateral diffusion path around a corneocyte	varied	
g	intercellular gap filled with lipids (μm)	0.075	
h	thickness of the corneocytes (μm)	0.8	
N	number of corneocyte layers	15	
ω	corneocyte offset; ratio of the long to short lateral diffusion paths around a corneocyte	different values used each permeant (see Table S9)	$\omega = d_l / d_s$ [a]
L	SC thickness	13.05	$L = Nh + (N-1)g$
τ_{volume}	tortuosity factor accounting for total volume of the branched, parallel pathways in the lipid domain	43.91	$\tau_{volume} = \frac{Nh + (N-1)(g+d)}{L}$
τ_{flux}	tortuosity factor accounting for the increased diffusion path length in circumnavigating the impermeable corneocytes	$1 + 42.91 \times \frac{\omega}{(1+\omega)^2}$	$\tau_{flux} = \frac{Nh}{L} + \frac{(N-1)}{L}\left(g + d\frac{\omega}{(1+\omega)^2}\right)$
ε	porosity	0.00187	$\varepsilon = d/(d+g)$

[a] ω = 1 when the offset is symmetrical (i.e., the diffusion path around the left and right sides of a corneocyte are equal) and $\omega \to \infty$ when corneocytes are completely aligned. In Figure S2, ω = 3.5.

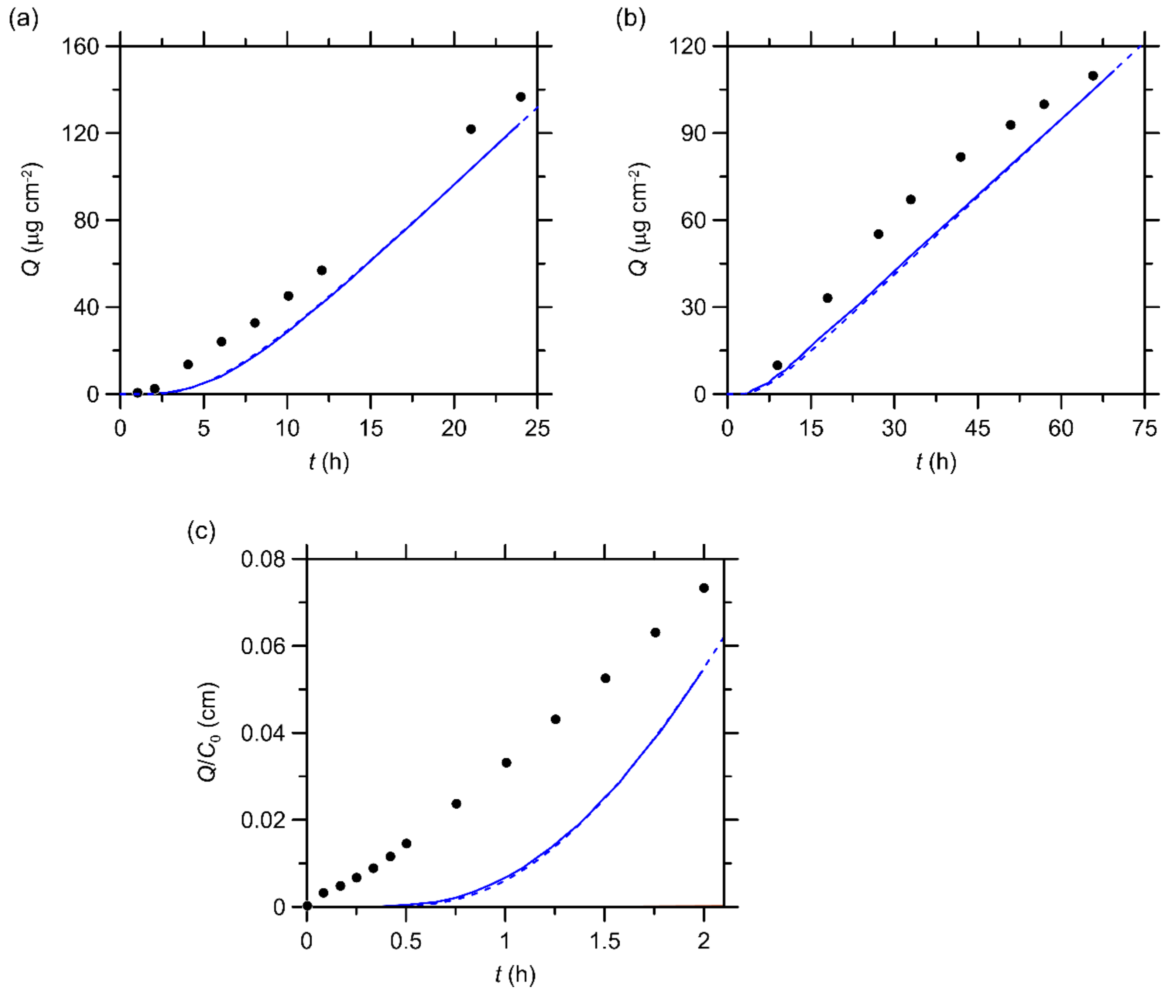

Figure S3. Cumulative mass of permeant released (Q) versus time calculated by Gajula et al. [32] (solid line) compared with experiments for (a) caffeine, (b) fentanyl, and (c) naphthol. Dashed lines represent calculations from Eq. (S.6) using t_{lag} and J_{ss} (or J_{ss}/C_o for naphthol) values listed in Table S9.

Table S9 lists the t_{lag} and J_{ss} (J_{ss}/C_0 for naphthol) values determined by fitting the Q versus t curves from Gajula et al. [32] presented in Figure S3. The Q versus t curves calculated using these t_{lag} and J_{ss} (or J_{ss}/C_0) values in Eq. (S.6) closely match the curves from Gajula et al. (see dashed lines compared with solid lines in Figure S3).

Table S9 also lists D_{eff} calculated from t_{lag} and the ratio of D_{eff} with the D_{lip} values from Gajula et al., [32] which range from about 50,000 to 320,000. These are significantly larger than the expected values of 230 to 515 calculated from the tortuosity parameters for the assumed SC brick-and-mortar configuration presented in Figure S2 (see Table S9). However, as shown in Table S9, the D_{eff}/D_{lip} values from Gajula et al. are within 25% of the square of the expected D_{eff}/D_{lip}, which is consistent with a calculation that adjusted D_{lip} for the SC configuration twice. Had D_{lip} been correctly adjusted for the SC configuration only once, the resulting D_{eff} (which range from 6.0 $\times$ 10^{-13} m^2 s^{-1} for caffeine to 9.2 $\times$ 10^{-13} m^2 s^{-1} for naphthol) would have been much too large to describe the experimental permeation data; i.e., t_{lag} is less than 1 min and J_{ss} is 200 to 600-fold larger than experimentally observed values (see the ‘expected’ values of t_{lag} and J_{ss} (or J_{ss}/C_0) in Table S9).

Table S9. D_{lip}/D_{eff} values calculated by Gajula et al. for caffeine, fentanyl and naphthol compared with D_{lip}/D_{eff} values expected for the SC geometry they used

	**Permeant**		
**Parameter**	**Caffeine**	**Fentanyl**	**Naphthol**
C_o (mg/mL) [a]	25.8	90	
t_{lag} (h) [b]	6.56	6.86	1.85
J_{ss} (μg cm^{-2} h^{-1}) [b]	7.15	1.78	
J_{ss} / C_o (cm h^{-1})	2.77×10^{-4}	1.98×10^{-5}	0.104 [b]
D_{eff} (m^2 s^{-1}) [c]	1.20×10^{-15}	1.15×10^{-15}	4.26×10^{-15}
D_{lip} (m^2 s^{-1}) [a]	2.37×10^{-10}	3.67×10^{-10}	2.12×10^{-10}
D_{lip}/D_{eff}	197×10^3	319×10^3	49.7×10^3
ω [a]	3	1	8
τ_{flux} [d]	9.05	11.73	5.24
τ_{volume} [d]	43.9	43.9	43.9
Expected value [e] for $D_{lip}/D_{eff} = \tau_{flux}\,\tau_{volume}$	397	515	230
Expected value [e] for $\left(D_{lip}/D_{eff}\right)^2 = \left(\tau_{flux}\,\tau_{volume}\right)^2$	158×10^3	265×10^3	52.9×10^3
Expected D_{eff} (m^2 s^{-1}) [f]	6.0×10^{-13}	7.1×10^{-13}	9.2×10^{-13}
Expected t_{lag} (s) [g]	48	40	31
Expected J_{ss} (μg cm^{-2} h^{-1}) [g]	1100	3550	
Expected J_{ss} / C_o (cm h^{-1}) [g]	0.14	0.012	22.4

[a] From Gajula et al.

[b] J_{ss} and t_{lag} calculated by fitting the Q versus t curves from Figure 6 in Gajula et al. (reproduced in Figure S3) to Eq. (S.6); for naphthol J_{ss}/C_0 was used instead because C_0 was not specified.

[c] Calculated from t_{lag} using Eq. (S.7)

[d] From Table S8

[e] From Eq. (S.4)

[f] Expected value of D_{eff} calculated from Eq. (S.4) for D_{lip} from Gajula et al.

[g] Value of t_{lag}, J_{ss}, and/or J_{ss}/C_0 for D_{eff} calculated from Eq. (S.4) for D_{lip} from Gajula et al. (i.e., the expected value of D_{eff}).

References

[1] Motta S, Monti M, Sesana S, Caputo R, Carelli S, Ghidoni R, Ceramide composition of the psoriatic scale, Biochim Biophys Acta 1182(2) (1993) 147-151.

[2] Schmitt T, Neubert RHH, State of the art in stratum corneum research: The biophysical properties of ceramides, Chem Phys Lipids (2018). https://doi.org/10.1016/j.chemphyslip.2018.09.017

[3] Kawana M, Miyamoto M, Ohno Y, Kihara A, Comparative profiling and comprehensive quantification of stratum corneum ceramides in humans and mice by LC/MS/MS, J Lipid Res 61(6) (2020) 884-895. https://doi.org/10.1194/jlr.RA120000671

[4] t'Kindt R, Jorge L, Dumont E, Couturon P, David F, Sandra P, Sandra K, Profiling and characterizing skin ceramides using reversed-phase liquid chromatography–quadrupole time-of-flight mass spectrometry, Anal Chem 84(1) (2012) 403-411. https://doi.org/10.1021/ac202646v

[5] Rabionet M, Bayerle A, Marsching C, Jennemann R, Gröne H-J, Yildiz Y, Wachten D, Shaw W, Shayman JA, Sandhoff R, 1-o-acylceramides are natural components of human and mouse epidermis, J Lipid Res 54(12) (2013) 3312-3321. https://doi.org/10.1194/jlr.M040097

[6] van Smeden J, Janssens M, Gooris GS, Bouwstra JA, The important role of stratum corneum lipids for the cutaneous barrier function, Biochim Biophys Acta-Lipids 1841 (2014) 295-313. https://doi.org/10.1016/j.bbalip.2013.11.006

[7] Vávrová K, Kováčik A, Opálka L, Ceramides in the skin barrier, 64(2) (2017) 28. https://doi.org/10.1515/afpuc-2017-0004

[8] Masukawa Y, Narita H, Shimizu E, Kondo N, Sugai Y, Oba T, Homma R, Ishikawa J, Takagi Y, Kitahara T, Takema Y, Kita K, Characterization of overall ceramide species in human stratum corneum, J Lipid Res 49(7) (2008) 1466-1476. https://doi.org/10.1194/jlr.M800014-JLR200

[9] Wertz PW, Downing DT, Ceramides of pig epidermis: Structure determination, J Lipid Res 24(6) (1983) 759-65.

[10] Law S, Wertz PW, Swartzendruber DC, Squier CA, Regional variation in content, composition and organization of porcine epithelial barrier lipids revealed by thin-layer chromatography and transmission electron microscopy, Arch Oral Biol 40 (1995) 1085-1091.

[11] Bouwstra JA, Gorris GS, Cheng K, Weerheim A, Bras W, Ponec M, Phase behavior of isolated skin lipids, J Lipid Res 37 (1996) 999-1011.

[12] Caussin J, Gooris GS, Janssens M, Bouwstra JA, Lipid organization in human and porcine stratum corneum differs widely, while lipid mixtures with porcine ceramides model human stratum corneum lipid organization very closely, Biochim Biophys Act- Biomembranes 1778(6) (2008) 1472-1482.

[13] Lavrijsen AP, Bouwstra JA, Gooris GS, Weerheim A, Bodde HE, Ponec M, Reduced skin barrier function parallels abnormal stratum corneum lipid organization in patients with lamellar ichthyosis, J Invest Dermatol 105(4) (1995) 619-624.

[14] Vičanová J, Weerheim AM, Ponec M, Boyce ST, Dana Harriger M, Bouwstra JA, Stratum corneum lipid composition and structure in cultured skin substitutes is restored to normal after grafting onto athymic mice, J Invest Derm Symp P 3(2) (1998) 114-120. https://doi.org/10.1038/jidsymp.1998.24

[15] Bleck O, Abeck D, Ring J, Hoppe U, Vietzke J-P, Wolber R, Brandt O, Schreiner V, Two ceramide subfractions detectable in Cer(AS) position by HPTLC in skin surface lipids of

non-lesional skin of atopic eczema, J Invest Dermatol 113(6) (1999) 894-900. https://doi.org/10.1046/j.1523-1747.1999.00809.x

[16] Weerheim A, Ponec M, Determination of stratum corneum lipid profile by tape stripping in combination with high-performance thin-layer chromatography, Arch Dermatol Res 293(4) (2001) 191-199. https://doi.org/10.1007/s004030100212

[17] Ponec M, Weerheim A, Lankhorst P, Wertz P, New acylceramide in native and reconstructed epidermis, J Invest Dermatol 120 (2003) 581-588. https://doi.org/10.1046/j.1523-1747.2003.12103.x

[18] Ishikawa J, Narita H, Kondo N, Hotta M, Takagi Y, Masukawa Y, Kitahara T, Takema Y, Koyano S, Yamazaki S, Hatamochi A, Changes in the ceramide profile of atopic dermatitis patients, J Invest Dermatol 130(10) (2010) 2511-2514. https://doi.org/10.1038/jid.2010.161

[19] Kováčik A, Roh J, Vávrová K, The chemistry and biology of 6-hydroxyceramide, the youngest member of the human sphingolipid family, ChemBioChem 15(11) (2014) 1555-1562. https://doi.org/10.1002/cbic.201402153

[20] van Smeden J, Hoppel L, van der Heijden R, Hankemeier T, Vreeken RJ, Bouwstra JA, LC/MS analysis of stratum corneum lipids: Ceramide profiling and discovery, J Lipid Res 52(6) (2011) 1211-1221. https://doi.org/10.1194/jlr.M014456

[21] Masukawa Y, Narita H, Sato H, Naoe A, Kondo N, Sugai Y, Oba T, Homma R, Ishikawa J, Takagi Y, Kitahara T, Comprehensive quantification of ceramide species in human stratum corneum, J Lipid Res 50(8) (2009) 1708-1719. https://doi.org/10.1194/jlr.D800055-JLR200

[22] Janssens M, van Smeden J, Gooris GS, Bras W, Portale G, Caspers PJ, Vreeken RJ, Kezic S, Lavrijsen APM, Bouwstra JA, Lamellar lipid organization and ceramide composition in the stratum corneum of patients with atopic eczema, J Invest Dermatol 131(10) (2011) 2136-2138.

[23] Janssens M, van Smeden J, Gooris GS, Bras W, Portale G, Caspers PJ, Vreeken RJ, Hankemeier T, Kezic S, Wolterbeek R, Lavrijsen AP, Bouwstra JA, Increase in short-chain ceramides correlates with an altered lipid organization and decreased barrier function in atopic eczema patients, J Lipid Res 53(12) (2012) 2755-2766. https://doi.org/10.1194/jlr.P030338

[24] van Smeden J, Al-Khakany H, Wang Y, Visscher D, Stephens N, Absalah S, Overkleeft HS, Aerts JMFG, Hovnanian A, Bouwstra JA, Skin barrier lipid enzyme activity in netherton patients is associated with protease activity and ceramide abnormalities, J Lipid Res 61(6) (2020) 859-869. https://doi.org/10.1194/jlr.RA120000639

[25] van Smeden J, Boiten WA, Hankemeier T, Rissmann R, Bouwstra JA, Vreeken RJ, Combined LC/MS-platform for analysis of all major stratum corneum lipids, and the profiling of skin substitutes, Biochim Biophys Acta-Lipids 1841(1) (2014) 70-79. https://doi.org/10.1016/j.bbalip.2013.10.002

[26] Moore TC, Hartkamp R, Iacovella CR, Bunge AL, McCabe C, Effect of ceramide tail length on the structure of model stratum corneum lipid bilayers, Biophys J 114(1) (2018) 113-125. https://doi.org/10.1016/j.bpj.2017.10.031

[27] Wang E, Klauda JB, Simulations of pure ceramide and ternary lipid mixtures as simple interior stratum corneum models, The Journal of Physical Chemistry B 122(10) (2018) 2757-2768. https://doi.org/10.1021/acs.jpcb.8b00348

[28] Wang E, Klauda JB, Models for the stratum corneum lipid matrix: Effects of ceramide concentration, ceramide hydroxylation, and free fatty acid protonation, The Journal of Physical Chemistry B (2018). https://doi.org/10.1021/acs.jpcb.8b06188

[29] Wang E, Klauda JB, Structure and permeability of ceramide bilayers and multilayers, J Phys Chem B 123(11) (2019) 2525-2535. https://doi.org/10.1021/acs.jpcb.9b00037

[30] Shamaprasad P, Moore TC, Xia D, Iacovella CR, Bunge AL, McCabe C, Multiscale simulation of ternary stratum corneum lipid mixtures: Effects of cholesterol composition, Langmuir (2022) submitted.

[31] Gunstone FD, Harwood JL, The lipid handbook with cd-rom, CRC Press, 2007.

[32] Gajula K, Gupta R, Sridhar DB, Rai B, In-silico skin model: A multiscale simulation study of drug transport, J. Chem Inf. Model. 57(8) (2017) 2027-2034. https://doi.org/10.1021/acs.jcim.7b00224

[33] Kushner J, Deen W, Blankschtein D, Langer R, First-principles, structure-based transdermal transport model to evaluate lipid partition and diffusion coefficients of hydrophobic permeants solely from stratum corneum permeation experiments, J Pharm Sci 96(12) (2007) 3236-3251. https://doi.org/10.1002/jps.20896
